# In-situ cryo-ET of mouse embryos reveals cytoplasmic lattices contain ubiquitin-charged E2-E3 ligase assemblies

**DOI:** 10.1038/s44318-026-00895-w

**Published:** 2026-08-10

**Authors:** Kashish Singh, Katarina Harasimov, Kathy K Niakan, Andrew P Carter

**Affiliations:** 1 MRC Laboratory of Molecular Biology, Cambridge, UK; 2 Loke Centre for Trophoblast Research, Department of Physiology, Development and Neuroscience, University of Cambridge, Cambridge, UK; 3 Sidney Sussex College, University of Cambridge, Cambridge, UK; 4 Wellcome-MRC Cambridge Stem Cell Institute, University of Cambridge, Cambridge, UK; 5 Epigenetics Programme, Babraham Institute, Cambridge, UK

**Keywords:** Development, Post-translational Modifications & Proteolysis, Structural Biology

## Abstract

Cytoplasmic lattices (CPLs) are filamentous assemblies essential for mammalian embryonic development. They are known to regulate organelle organization, spindle assembly, and protein homeostasis, but their molecular functions remain unclear. Here, we develop a strategy combining cryo-focused ion beam milling and cryo-electron tomography to resolve macromolecular complexes directly in mammalian embryos. Using this approach, we determine the in situ structure of cytoplasmic lattices within 6/8-cell mouse embryos at ~4.7 Å resolution. CPL filaments are built from multiple copies of at least fourteen proteins arranged into a ~4.5 MDa repeating unit. The repeat contains a central cavity that is open at the back and lined with multiple FBXW–SKP1 complexes and three modules, each containing the E2 ubiquitin-conjugating enzyme UBE2D and the E3 ligase UHRF1. We resolve two CPL states: one is consistent with a ubiquitin-charged UBE2D, where ubiquitin is held in an open, inactive conformation by binding the scaffold protein PADI6; the second lacks discernible ubiquitin density and shows structural changes compatible with ubiquitin becoming available for transfer. Our findings support a model in which CPLs function as large ubiquitin ligase assemblies during early embryonic development.

## Synopsis

Cytoplasmic lattices (CPLs), abundant filamentous assemblies within mammalian oocytes and early embryos, are essential for development, yet their composition and function remain unclear. Here, in-situ cryo-electron tomography solves the structure of CPLs within 6/8-cell mouse embryos and reveals their involvement in regulation of ubiquitination.

- In-situ structure of CPLs from mouse embryo blastomeres reveals a ~4.5 MDa repeating unit composed of at least 14 proteins.
- Within each repeat, PADI6 and subcortical maternal complex proteins form a scaffold that encloses a central cavity lined with FBXW-SKP1 complexes and modules of UHRF1, UBE2D, NLRP14 and αβ-tubulin.
- The central cavity exists either in a state with ubiquitin-charged UBE2D, where ubiquitin is bound to PADI6, or in one containing a central FBXW-SKP1 complex and lacking ubiquitin density.
- CPL filaments organize into lattices through interactions between a PADI6-bound FBXW-SKP1 complex on one filament and UHRF1-NLRP14 on the back face of another.

## Abstract (web summary)

Structures of cytoplasmic lattices from early mouse embryos reveal the presence of ubiquitin conjugation complexes in two conformational states.

## Graphical abstract

**Figure:**
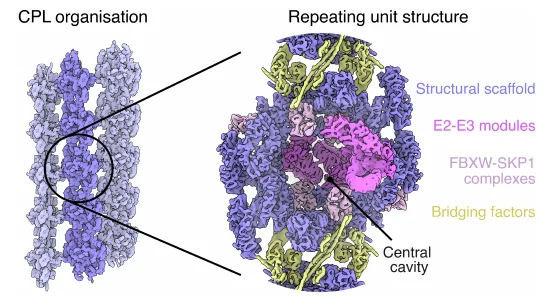

## Introduction

Early mammalian development is driven by maternally deposited factors, as the embryonic genome remains inactive for the first several days (Gerri et al, 2020). A critical set of maternal components is filamentous structures known as cytoplasmic lattices (CPLs) (Calarco and Brown, 1969; Enders and Schlafke, 1965; Gallicano et al, 1992; Schlafke and Enders, 1967). In mice, CPL loss results in developmental arrest shortly after fertilization (Li et al, 2008; Tashiro et al, 2010; Uemura et al, 2023; Yu et al, 2014; Yurttas et al, 2008; Zhang et al, 2023). In humans, mutations in CPL-associated proteins are linked to early embryonic arrest and infertility (Qian et al, 2018; Tannorella et al, 2022; Xu et al, 2016), as well as miscarriage (Demond et al, 2019; Qian et al, 2018; Rezaei et al, 2021) and multilocus imprinting syndromes (Begemann et al, 2018; Cubellis et al, 2020; Eggermann et al, 2021; Eggermann et al, 2022; Tannorella et al, 2022).

CPLs have been implicated in diverse cellular processes. Loss of CPL components disrupts organelle organization (Gao et al, 2018; Kan et al, 2011; Qin et al, 2019) and spindle assembly during oocyte meiosis and early embryonic divisions (Cao et al, 2019; Kan et al, 2011; Yu et al, 2014; Zheng and Dean, 2009). CPL disruption has also been shown to impair proteostasis, leading to widespread changes in the abundance of diverse proteins, including ribosomal subunits, mitochondrial proteins, metabolic enzymes, and cytoskeletal factors (Jentoft et al, 2023; PREPRINT: Williams et al, 2026). However, the molecular mechanisms through which CPLs affect these cellular processes remain unclear.

CPL filaments exhibit a highly regular ~38 nm repeating unit (Jentoft et al, 2023) and assemble into higher-order lattice-like arrays within the cytoplasm (Calarco and Brown, 1969; Enders and Schlafke, 1965; Schlafke and Enders, 1967). Genetic and light microscopy studies suggest that this repetitive structure is composed of the putative peptidylarginine deiminase PADI6 and the subcortical maternal complex (SCMC) (Jentoft et al, 2023; Tashiro et al, 2010; Yurttas et al, 2008). The SCMC has been reported to contain an NLRP5–TLE6–OOEP complex, KHDC3 and ZBED3 proteins, and multiple NLRP family proteins, including NLRP2, NLRP4B, NLRP4F, and NLRP9 (Chi et al, 2024; Gao et al, 2018; Han et al, 2023; Kanzaki et al, 2020; Li et al, 2008; Mahadevan et al, 2017; Nic Aodha et al, 2025; Ohsugi et al, 2008; Ou et al, 2025; Tannorella et al, 2022). In addition, more than 200 proteins whose abundance decreases upon CPL disruption have been proposed to be sequestered and stored within CPLs, where they are protected from degradation (Jentoft et al, 2023; PREPRINT: Williams et al, 2026). Interestingly, some of these proposed stored factors, such as NLRP14 and the E3 ubiquitin ligase UHRF1, are themselves required for CPL formation (Uemura et al, 2023; Zhang et al, 2023). These observations raise key questions about which proteins are incorporated into CPL filaments, how they are organized within the repeating unit, and how this organization relates to CPL function.

Determining the molecular architecture of CPL filaments in their native environment has proven challenging. The scarcity of mammalian oocytes and embryos and the large size of these cells (~80 µm in mice and ~125 µm in humans) complicates in situ structural analysis by cryo-electron tomography (cryo-ET), as preparing lamellae by cryo-focused ion beam (cryo-FIB) milling must typically be performed manually, limiting throughput. Consequently, the only reported in situ reconstruction of CPLs, obtained from oocytes (Jentoft et al, 2023), was limited to ~30 Å resolution, insufficient for unambiguous protein identification.

Here, we develop a workflow enabling high-throughput cryo-FIB and cryo-ET for structural analysis of early mammalian embryos. Using this approach, we determine the molecular architecture of CPLs within the native embryonic cytoplasm. The structure reveals that the CPL repeating unit forms a ~4.5 MDa assembly containing at least 14 different proteins, including αβ-tubulin and multiple proteins involved in ubiquitination. The arrangement of these components creates a large central cavity that contains three E2-E3 ubiquitin ligase complexes. We resolve two structural states of this central cavity, including one in which we observe density consistent with the E2 being charged with ubiquitin. Overall, our structure provides the molecular framework for understanding CPL function and suggests that they play a role in ubiquitin-dependent regulation during early development.

## Results

### In situ cryo-ET of early mouse embryos

**Figure 1:**
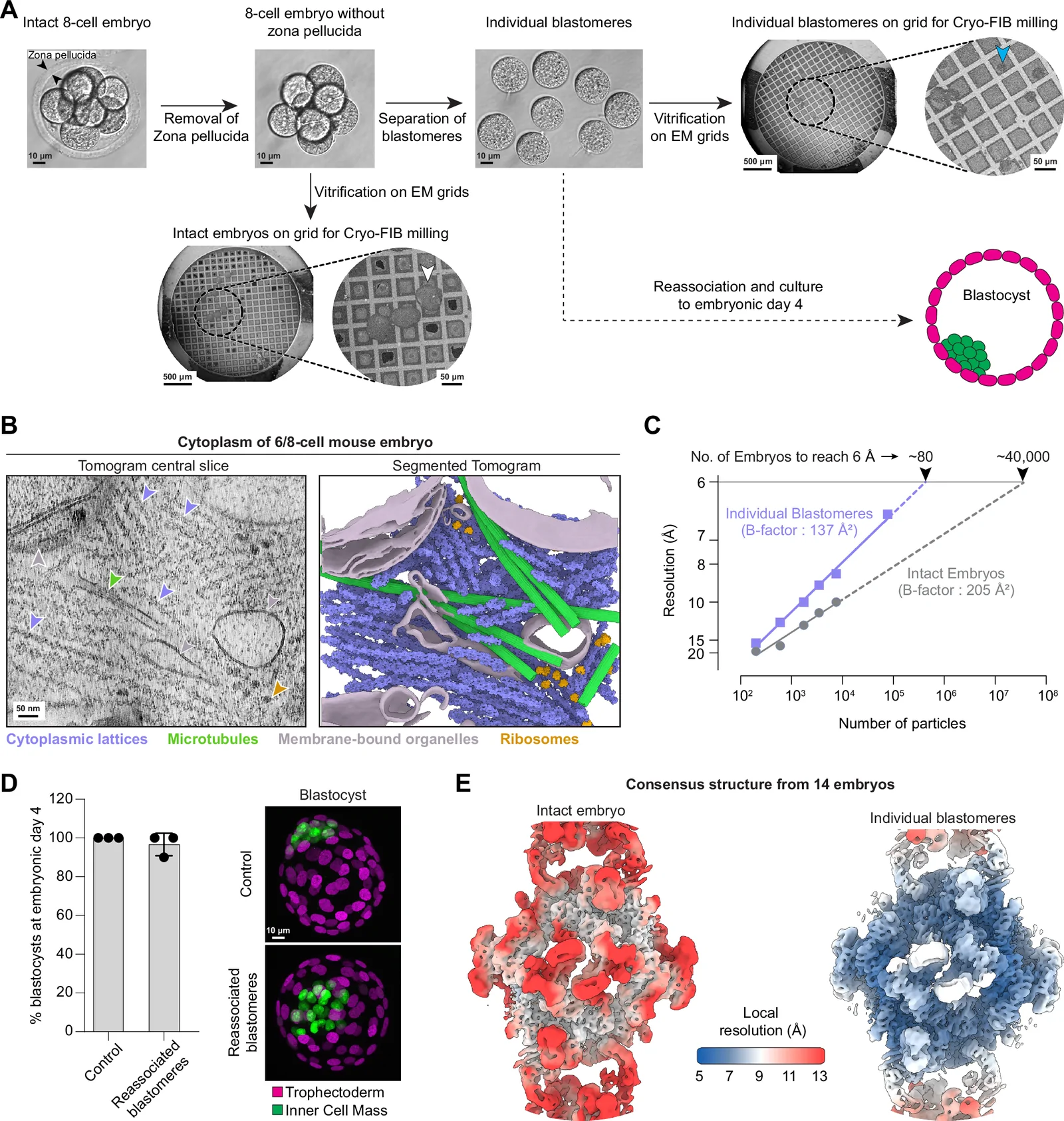
In situ structural investigation of CPLs from early mouse embryos. (**A**) Schematic for preparation of 6/8-cell stage mouse embryos for cryo-FIB milling directly or after separation into individual blastomeres. The white and green arrowheads highlight an example of an intact embryo and an individual blastomere vitrified on an EM grid, respectively. (**B**) Central slice of a denoised tomogram depicting the cytoplasm within a blastomere of a 6/8-cell stage mouse embryo (left) with colored arrowheads pointing to features in the corresponding segmentation of the tomogram (right). (**C**) Rosenthal–Henderson plot comparing datasets from intact embryos and individual blastomeres, demonstrating that data from individual blastomeres require fewer particles to achieve equivalent resolution. The estimated number of embryos required to reach 6 Å resolution was calculated from the average number of particles obtained per embryo used. (**D**) Efficiency of blastocyst formation upon blastomere reassociation, as shown in (**A**), compared to control samples where only the zona pellucida was removed (left). Immunofluorescence images of embryos at the blastocyst stage from the two groups (right). Trophectoderm is marked by GATA3 (magenta), and inner cell mass by SOX2 (green). Three independent replicates are shown as dots; mean ± s.d. are indicated. A minimum of five embryos were analyzed per group in each replicate. (**E**) Subtomogram averaging density map of CPL repeat unit solved from intact embryos (left) and the corresponding map from individual blastomeres (right) colored based on their local resolution. Source data are available online for this figure.

**Figure EV1:**
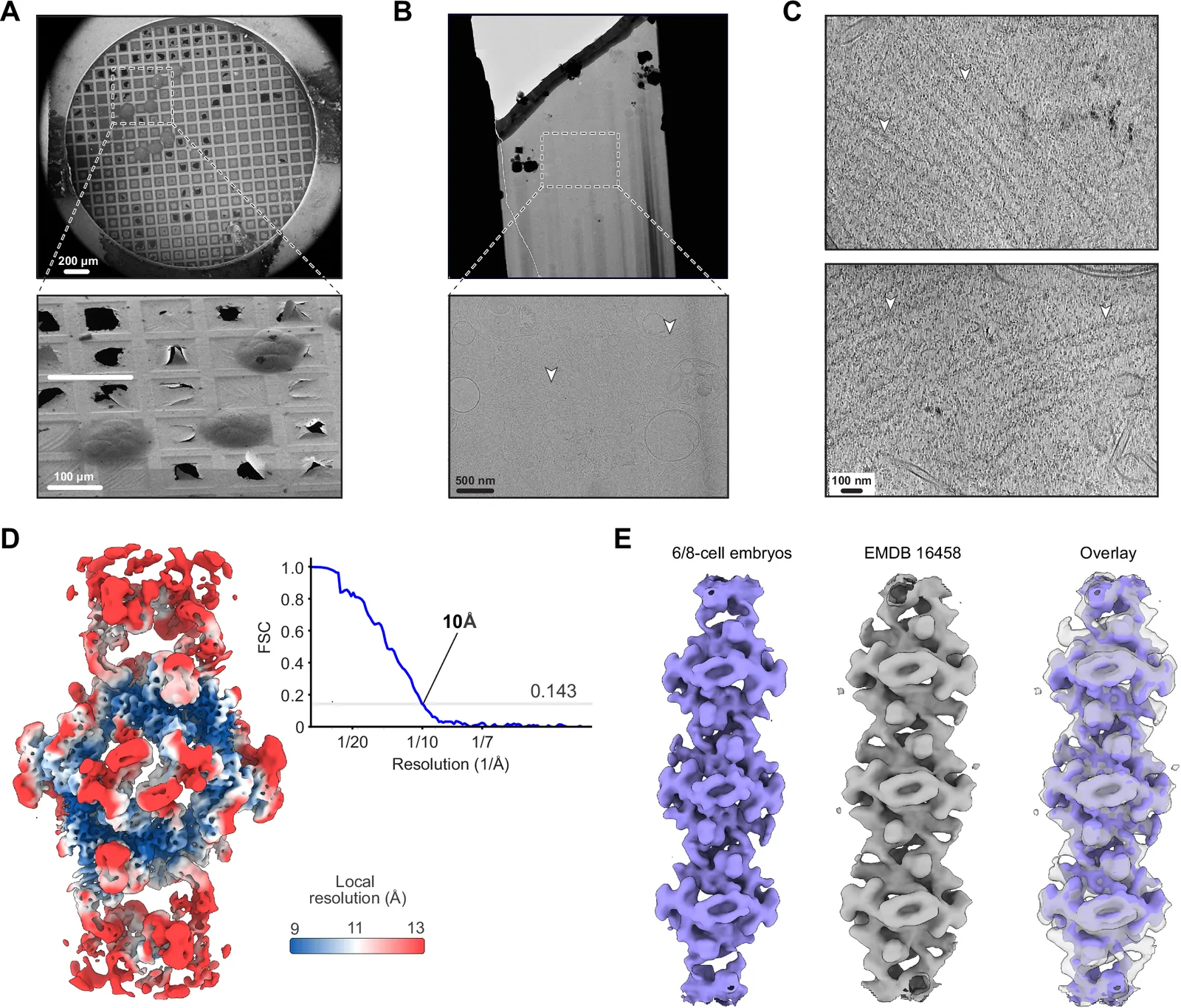
Structure of CPL filaments in 6/8-cell mouse embryos. (**A**) Scanning electron microscope (SEM) image of vitrified 6/8-cell mouse embryos on EM grids (top), and a FIB image showing three embryos on the grid (bottom). (**B**) Low-magnification transmission electron microscope (TEM) image of a representative lamella (top) and a zoomed-in view highlighting a few examples of CPLs with white arrowheads (bottom). (**C**) Representative denoised tomographic slices showing CPLs with a few examples highlighted with white arrowheads. (**D**) Subtomogram averaging map of the CPL repeat unit from cryo-ET data acquired on a lamella of intact mouse embryos, colored by local resolution. The corresponding Fourier shell correlation (FSC) curve is shown. (**E**) Comparison of CPL filament structure obtained using subtomogram averaging in early mouse embryos (left; this study) and from oocytes (center; EMDB-16458 (Jentoft et al, 2023)). The two maps are overlaid (right), with EMDB-16458 rendered semi-transparent.

We initially vitrified 6/8-cell stage mouse embryos directly on electron microscopy grids using a published cryopreservation protocol (Jentoft et al, 2023) that utilizes 7.5% dimethyl sulfoxide and 7.5% ethylene glycol as cryoprotectants (Figs. 1A and EV1A). We prepared lamellae from intact embryos by manual cryo-FIB milling and used cryo-ET to visualize macromolecular structures within the embryonic cytoplasm (Figs. 1B and EV1B,C). We collected 36 tomograms from 14 embryos and reconstructed CPL filaments at 10 Å resolution by subtomogram averaging (Fig. EV1D). The resulting map closely resembled the previously reported 30 Å reconstruction from mouse oocytes (Jentoft et al, 2023) (Fig. EV1E), indicating that CPL filament architecture is conserved between oocytes and cleavage-stage embryos.

**Figure EV2:**
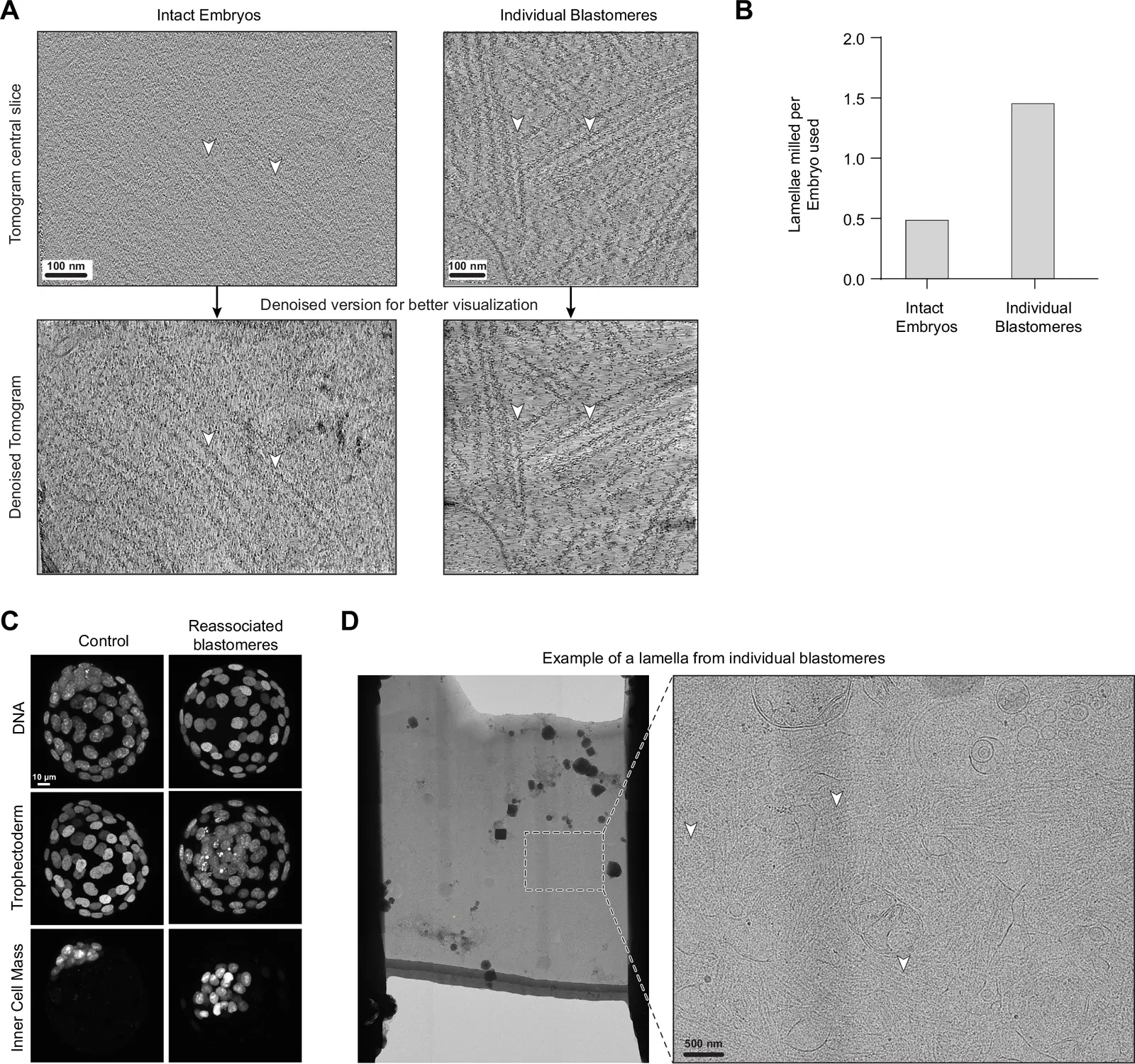
Cryo-ET of 6/8-cell embryo blastomeres. (**A**) Central slices of tomograms without and with denoising showing the improvement in contrast in data collected from individual blastomeres (right) as compared to that from intact embryos (left). Both tomograms were collected at a defocus of 4 µm and a lamella thickness of 130–140 nm. (**B**) Graphs comparing the total number of lamellae obtained relative to the number of embryos used for vitrification on EM grids, for intact embryos (left) and embryos separated into individual blastomeres (right). Not all vitrified embryos/blastomeres yielded lamellae, as a fraction were lost during vitrification or located in regions of the grid unsuitable for cryo-FIB milling. (**C**) Immunofluorescence images showing the individual channels corresponding to the merged image in Fig. 1D. (**D**) Low-magnification TEM image of a representative lamella prepared from a blastomere (left) and a zoomed-in view highlighting few examples of CPLs with white arrowheads (right).

However, the cryoprotectant concentrations required for vitrification of intact embryos reduced image contrast (Fig. EV2A), and the requirement for manual cryo-FIB milling limited the number of lamellae that could be prepared (Fig. EV2B). A Rosenthal–Henderson plot (Rosenthal and Henderson, 2003) of resolution versus particle number shows that achieving ~6 Å resolution, sufficient to resolve secondary structure features such as α-helices, would require an unfeasibly large amount of material (~40,000 embryos) (Fig. 1C).

To overcome these limitations, we developed a workflow in which embryos were gently separated into individual blastomeres immediately prior to vitrification (Fig. 1A). Isolated blastomeres remained morphologically intact and formed blastocysts upon reassociation, showing that separation preserved cellular integrity and developmental potential (Figs. 1A,D and EV2C). Blastomere separation is routinely used for single-cell RNA sequencing (Simon et al, 2025), and similar strategies have also recently enabled in situ structural analysis of cells isolated from complex tissues such as Drosophila ovaries (Sachweh et al, 2025). Importantly, blastomere separation enabled high-throughput automated cryo-FIB milling (Dutka and Prokhodtseva, 2019), increased the number of lamellae obtained per embryo (Fig. EV2B,D), and led to an approximately threefold reduction in the required cryoprotectant concentration, which improved image contrast (Fig. EV2A). Using blastomeres from only 14 embryos, we resolved CPL filaments at 6.6 Å resolution using 180 tomograms (Fig. 1E). Importantly, the CPL subtomogram density map obtained from individual blastomeres remained consistent with that resolved from intact embryos (Fig. 1E), showing that the structure is not affected by blastomere separation. Moreover, the improved data quality reduced material requirements for high-resolution analysis, such that we estimated ~80 embryos would be sufficient to achieve sub-6 Å reconstructions (Fig. 1C).

**Figure EV3:**
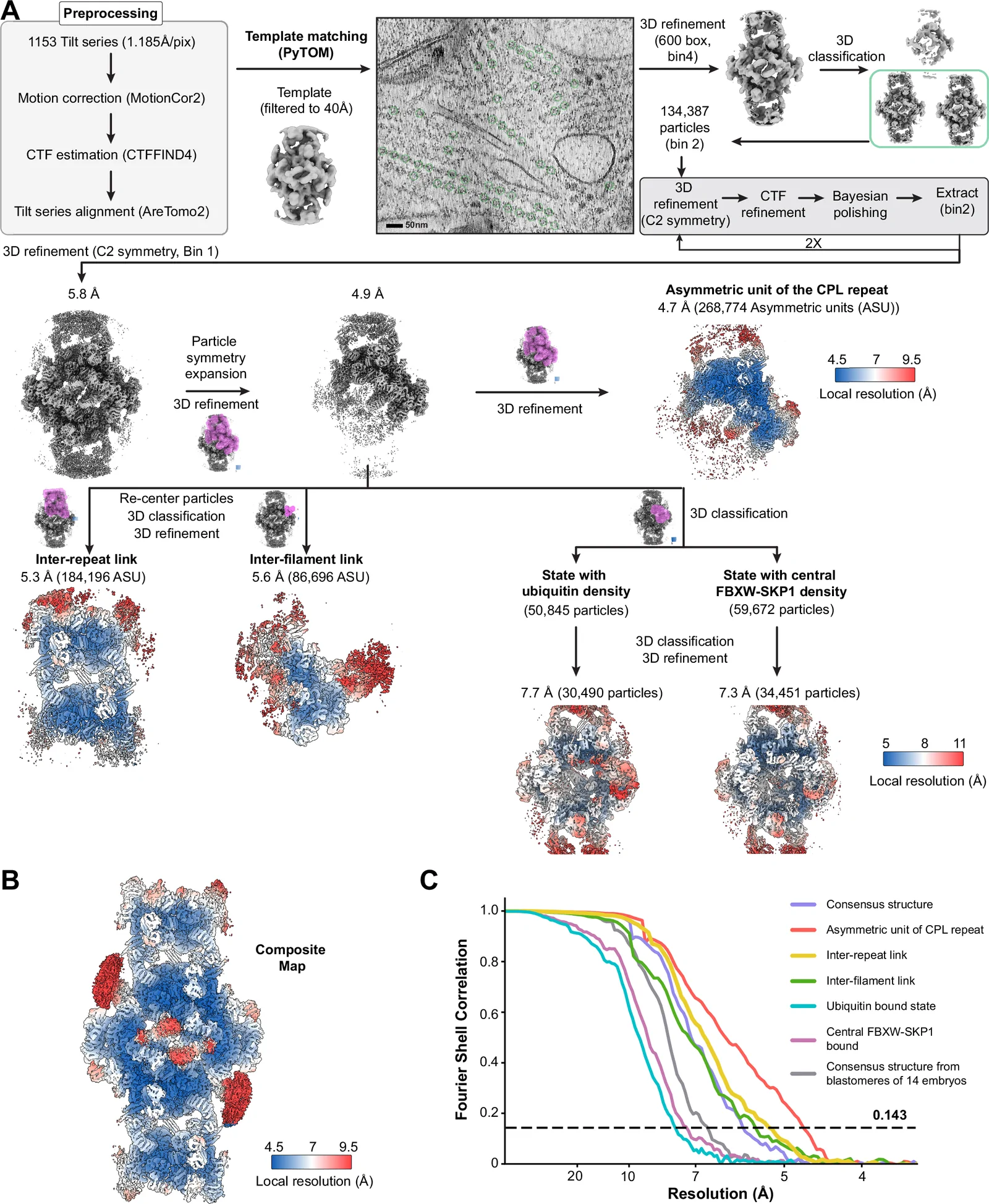
Subtomogram averaging processing pipeline. (**A**) The workflow for resolving the structure of the CPL repeat is shown. (**B**) The composite map generated by combining maps obtained after focused 3D refinements is depicted and colored based on local resolution. (**C**) Plot shows the gold-standard Fourier Shell Correlation of the different subtomogram averaging maps shown in (**A**). The dotted horizontal line shows the 0.143 cut-off.

**Figure 2:**
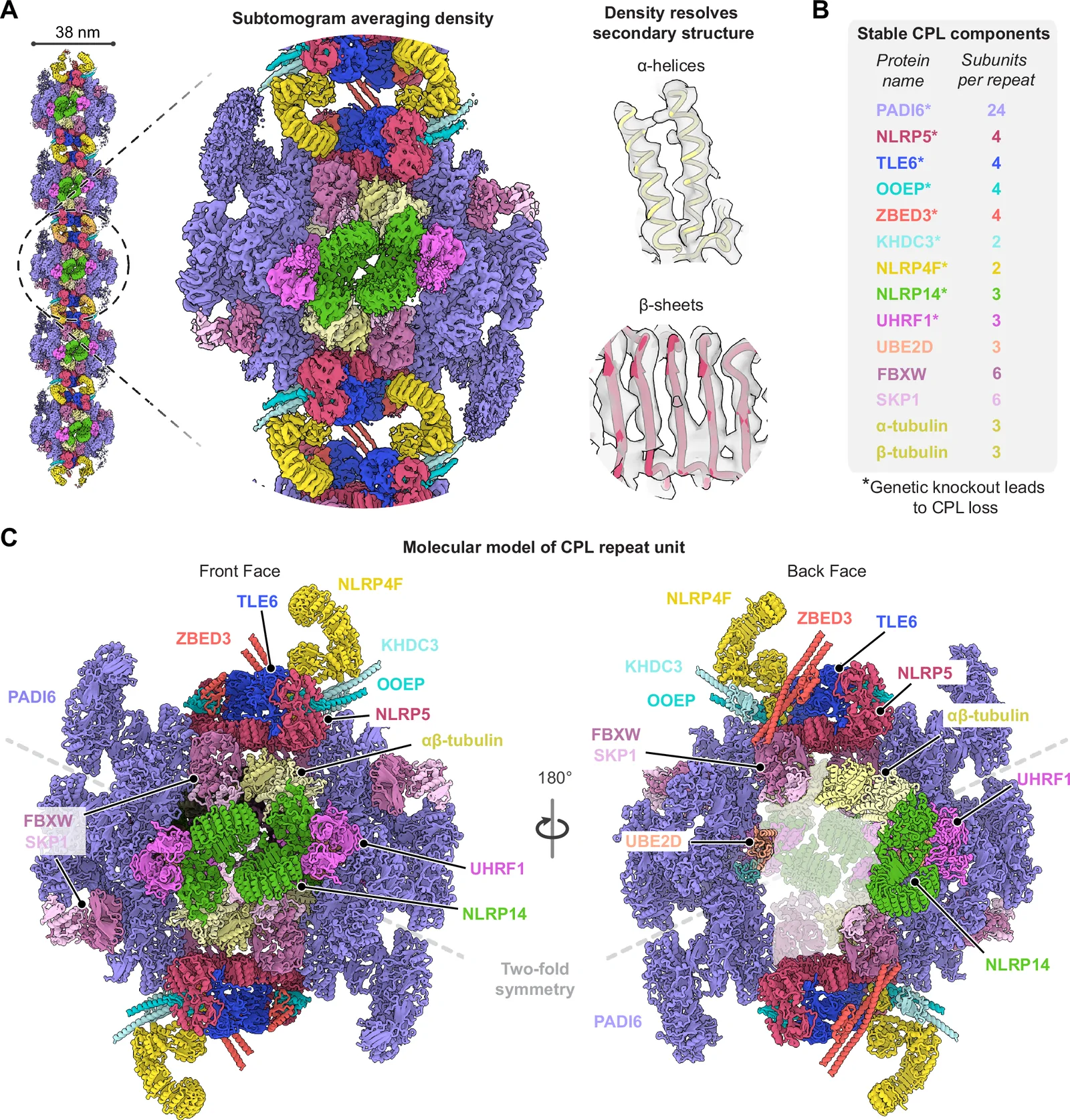
Structure of the CPL filaments. (**A**) Subtomogram averaging composite map of the CPL repeat unit. Examples of structural features resolved in the map are shown on the right. (**B**) Stable components identified within CPL filaments and their copy number per repeat. (**C**) Atomic model of the CPL repeat unit viewed from the front (left) and back (right), showing its overall organization. The overall protein arrangement exhibits a pseudo twofold symmetry, with the two symmetric halves separated by the gray dotted line.

To obtain a higher resolution structure of CPLs, we collected a large dataset of 1153 tomograms from blastomeres derived from 66 embryos, yielding a consensus subtomogram average at 5.8 Å resolution (Fig. EV3). To account for conformational heterogeneity and flexibility between different regions of the filament, we performed particle symmetry expansion along with focused 3D classification and refinement, which further improved map quality to ~4.7 Å resolution (Fig. EV3A; Appendix Table S1). We combined the resulting locally refined maps into a composite reconstruction (Fig. 2A; Movie EV1) with local resolution predominantly ranging from 4.5 to 6.0 Å (Fig. EV3B,C). Overall, the data demonstrate that our workflow enables robust in situ structure determination within mammalian embryo blastomeres while preserving the native architecture of macromolecular complexes.

### Molecular composition of CPL filaments in early mouse embryos

**Figure EV4:**
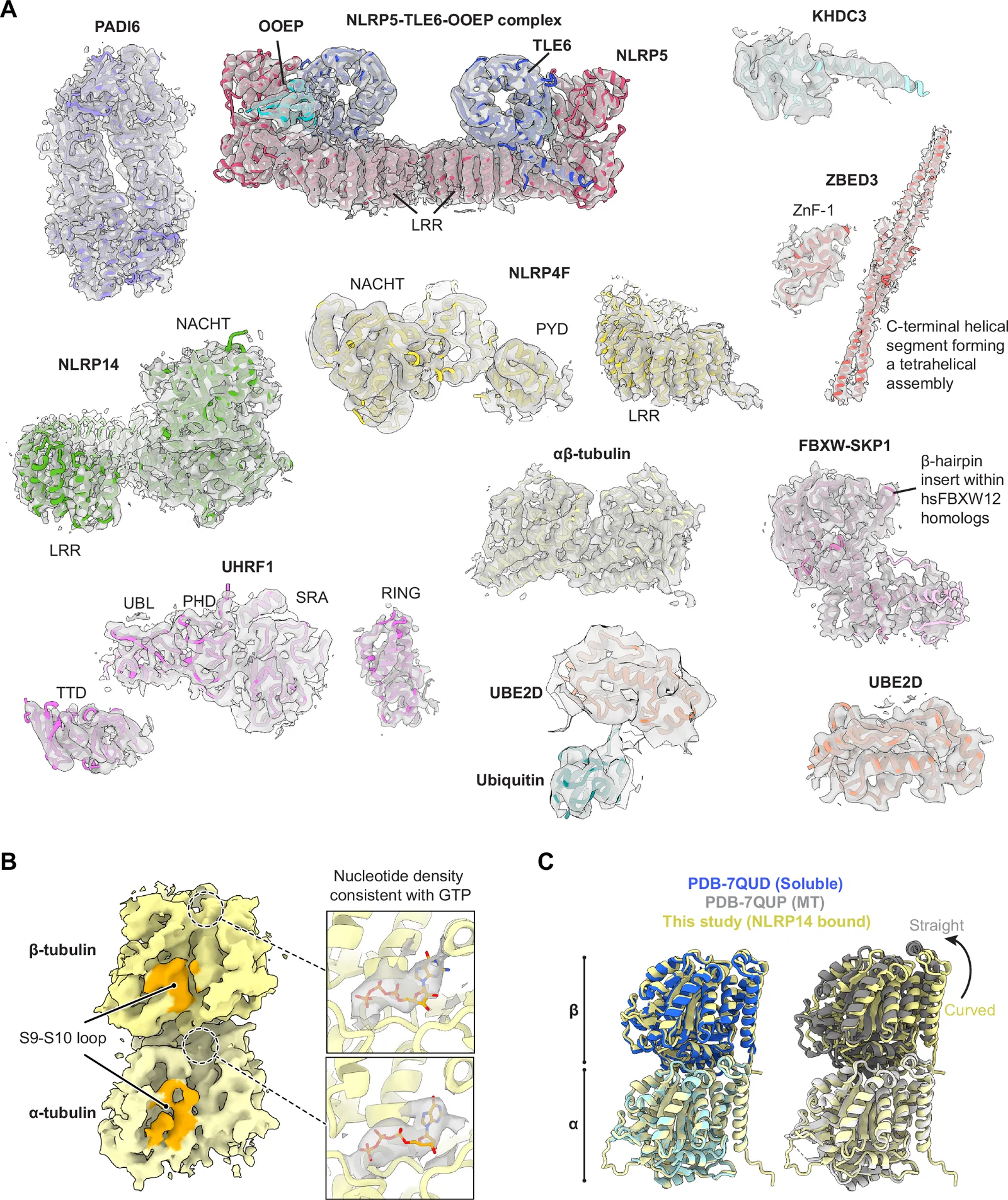
Densities of proteins identified within CPLs. (**A**) Gallery showing individual proteins with their atomic models fitted into the corresponding subtomogram averaging density maps. (**B**) Density of the αβ-tubulin showing the S9–S10 loop in orange. The loop is shorter in the density assigned to β-tubulin, as expected. Insets show the modeled GTP in the corresponding density at the N-site (α/β intradimer interface; bottom) and the E-site (β-tubulin; top). (**C**) Structural comparison of αβ-tubulin from the CPL repeat with soluble (PDB 7QUD (Wagstaff et al, 2023)) and microtubule-incorporated tubulin dimers (PDB 7QUP (Wagstaff et al, 2023)), showing that CPL-bound αβ-tubulin adopts a conformation resembling the soluble form.

Our composite subtomogram averaging reconstruction displays well-resolved secondary structure features, including clear densities corresponding to α-helices and β-sheets (Figs. 2A and EV4A). This structural information was sufficient to assign all densities within the CPL filament repeat unit and build its molecular model. Each 38 nm repeat contains at least 14 distinct proteins present in multiple copies, resulting in a total of 71 subunits. These include PADI6, components of the SCMC, proteins involved in ubiquitination, NLRP14, and αβ-tubulin heterodimers (Fig. 2B). Subunits on the front face are arranged with twofold symmetry, whereas the symmetry on the back face is incomplete (Fig. 2C). The combined molecular mass of each repeat unit is ~4.5 MDa, placing CPLs among the largest known repeating units of a biological filament and, to our knowledge, second only to the axonemal 96 nm repeat (Walton et al, 2023).

The proteins within the CPL repeat were identified through a candidate-based approach focusing on proteins implicated with CPLs. The rationale for each assignment is summarized in “Methods” section. We began with factors directly implicated in CPL formation, namely PADI6 and the SCMC (Jentoft et al, 2023; Tashiro et al, 2010; Yurttas et al, 2008), reasoning that these were most likely to be a part of the filaments. We identified 24 copies of PADI6 per repeat unit, making it the most abundant component of the CPL filament (Fig. 2B,C). We further found only a subset of SCMC proteins, specifically the NLRP5–TLE6–OOEP complex, along with KHDC3, ZBED3, and NLRP4F (Fig. 2B,C). Other proteins considered a part of the SCMC, such as NLRP2 (Mahadevan et al, 2017; Ou et al, 2025), NLRP4B (Nic Aodha et al, 2025), and NLRP9 (Kanzaki et al, 2020), were not resolved in our subtomogram average density, suggesting that they may perform functions outside the assembled CPL filaments.

UHRF1 and NLRP14 are also known to be important for CPL formation (Uemura et al, 2023; Zhang et al, 2023). UHRF1 is an E3 ubiquitin ligase best known for its nuclear role in maintenance of DNA methylation. It recognizes hemi-methylated DNA and ubiquitinates histone H3 and PCNA-associated factor 15, to promote DNA methyltransferase 1 recruitment to newly replicated DNA (Bostick et al, 2007; Nishiyama et al, 2020; Sharif et al, 2007). However, in oocytes and early embryos, it is predominantly cytoplasmic due to its association with CPLs (Maenohara et al, 2017; Uemura et al, 2023). NLRP14 has been implicated in stabilizing UHRF1 in the cytoplasm by protecting it from degradation (Zhang et al, 2023). Although both proteins were proposed to be stored on CPLs, we found that UHRF1 and NLRP14 are integral components of the CPL repeat unit (Fig. 2B, C), explaining why their loss leads to the disappearance of CPLs (Uemura et al, 2023; Zhang et al, 2023).

The remaining unassigned densities included additional components of the ubiquitination machinery. Density next to UHRF1 fit with a member of the E2 ubiquitin-conjugating enzyme UBE2D-family (DaRosa et al, 2018; Li et al, 2026a). We further identified six copies of a complex consisting of a WD40-containing F-box protein and SKP1. F-box proteins mediate substrate recognition within SKP1-CUL1-F-box (SCF) Cullin-RING ligases (Harper and Schulman, 2021; Rusnac and Zheng, 2020; Skaar et al, 2013), whereas SKP1 links the F-box proteins to the Cullin scaffold (Rusnac and Zheng, 2020). Our density shows a beta-hairpin insert into the WD40 domain that is consistent with the family of proteins homologous to the human FBXW12 protein (Fig. EV4A). In mice, there are multiple proteins in this family, including FBXW15, whose levels decrease upon knockout of PADI6 (Jentoft et al, 2023). However, at our obtained resolution, we cannot discriminate between them and so refer to these components as FBXW.

We also identified an αβ-tubulin heterodimer as a structural element of the CPL repeat unit (Fig. EV4B), representing, to our knowledge, the only assembly outside microtubules in which αβ-tubulin serves as a stable architectural component. The CPL-bound αβ-tubulin adopts a conformation resembling soluble rather than microtubule-bound tubulin (Fig. EV4C). At the resolution of the density map, we could not determine whether it corresponds to a single αβ-tubulin isoform pair or a mixture of different α- and β-tubulin isoforms. Our density is consistent with GTP bound at both canonical nucleotide-binding sites: the non-exchangeable N-site in α-tubulin, located at the α/β intradimer interface, and the exchangeable E-site in β-tubulin (Fig. EV4B). Overall, CPLs incorporate a soluble-like GTP-bound αβ-tubulin heterodimer as a stable architectural component of the repeat.

Notably, of these proteins, all except ZBED3, NLRP4F, and FBXW are known to be enriched on CPLs by immunofluorescence (Jentoft et al, 2023), supporting their localization to this structure. The assignments of the remaining three are supported by previously reported interactions of ZBED3 and NLRP4F with the NLRP5–TLE6–OOEP complex (Ou et al, 2025; Qin et al, 2019), and of FBXW proteins with SKP1 (Rusnac and Zheng, 2020). Importantly, ZBED3 and NLRP4F knockout mice are also known to lack CPLs in oocytes (Gao et al, 2018; Qin et al, 2019).

Overall, our structure shows that CPL filaments are made of at least fourteen proteins and defines their architecture.

### CPL repeats are built around PADI6 and NLRP5–TLE6–OOEP scaffold

**Figure 3:**
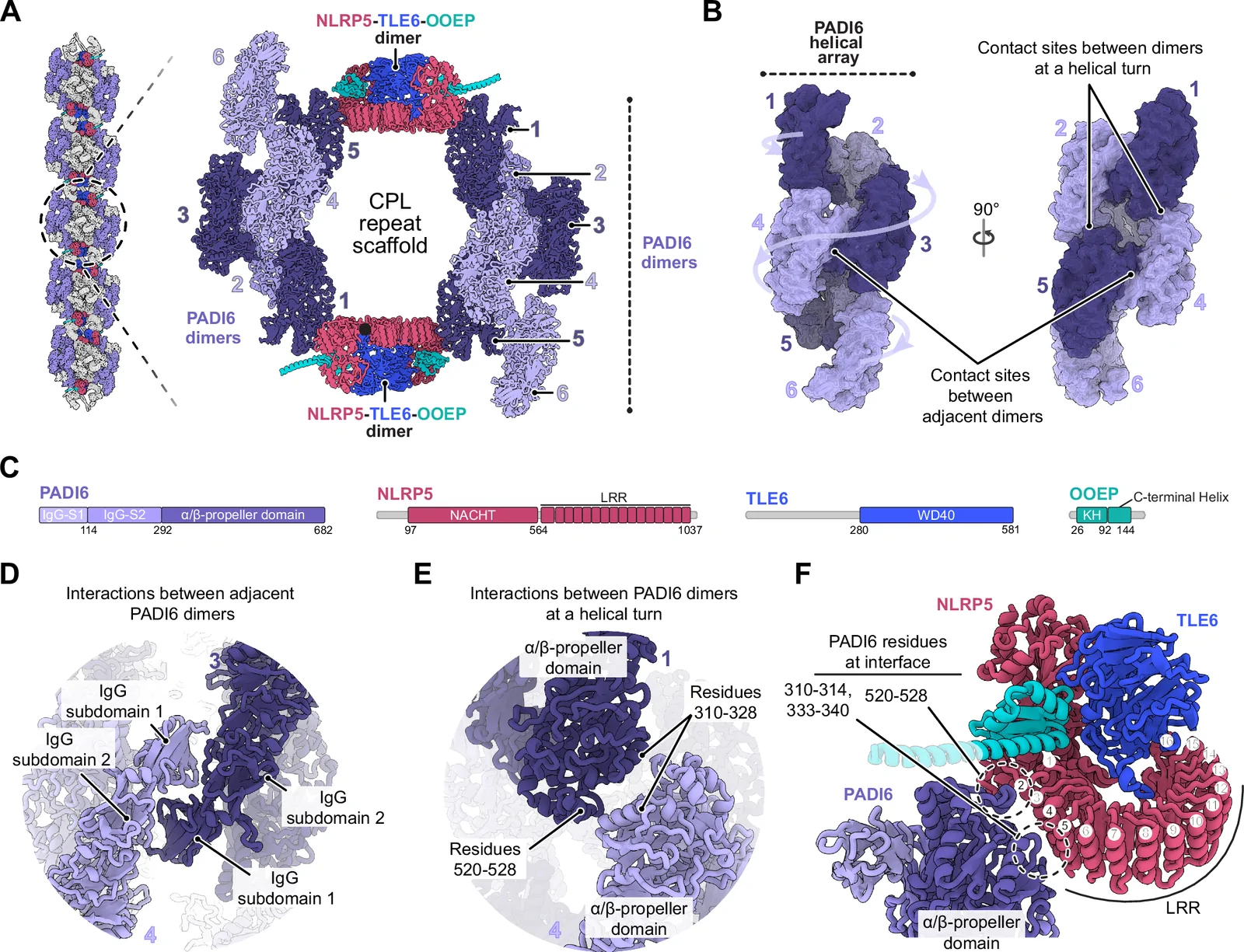
Organization of the proteins that scaffold CPL repeat units. (**A**) Arrangement of PADI6, NLRP5, TLE6, and OOEP within a single CPL repeat unit. The six PADI6 dimers are labeled 1–6 and shaded in two shades of lavender to distinguish them. Proteins in the core are hidden for clarity. (**B**) Helical array formed by PADI6 dimers, highlighting the two types of interdimer contacts. (**C**) Domain architecture of the CPL scaffold proteins. IgG-S immunoglobulin subdomain, LRR leucine-rich repeat, WD40 WD-repeat domain. KH K-homology. (**D**) Close-up view of the IgG subdomains at the interface between adjacent PADI6 dimers. (**E**) PADI6 α/β-propeller domains at positions where the helix completes a turn (i.e., between dimers 1–4 and 2–5). (**F**) Interaction sites between NLRP5–TLE6–OOEP complex and PADI6.

Each CPL repeat unit is organized around a scaffold composed of PADI6 and the NLRP5–TLE6–OOEP complex (Fig. 3A). All remaining CPL components assemble onto this scaffold.

**Figure EV5:**
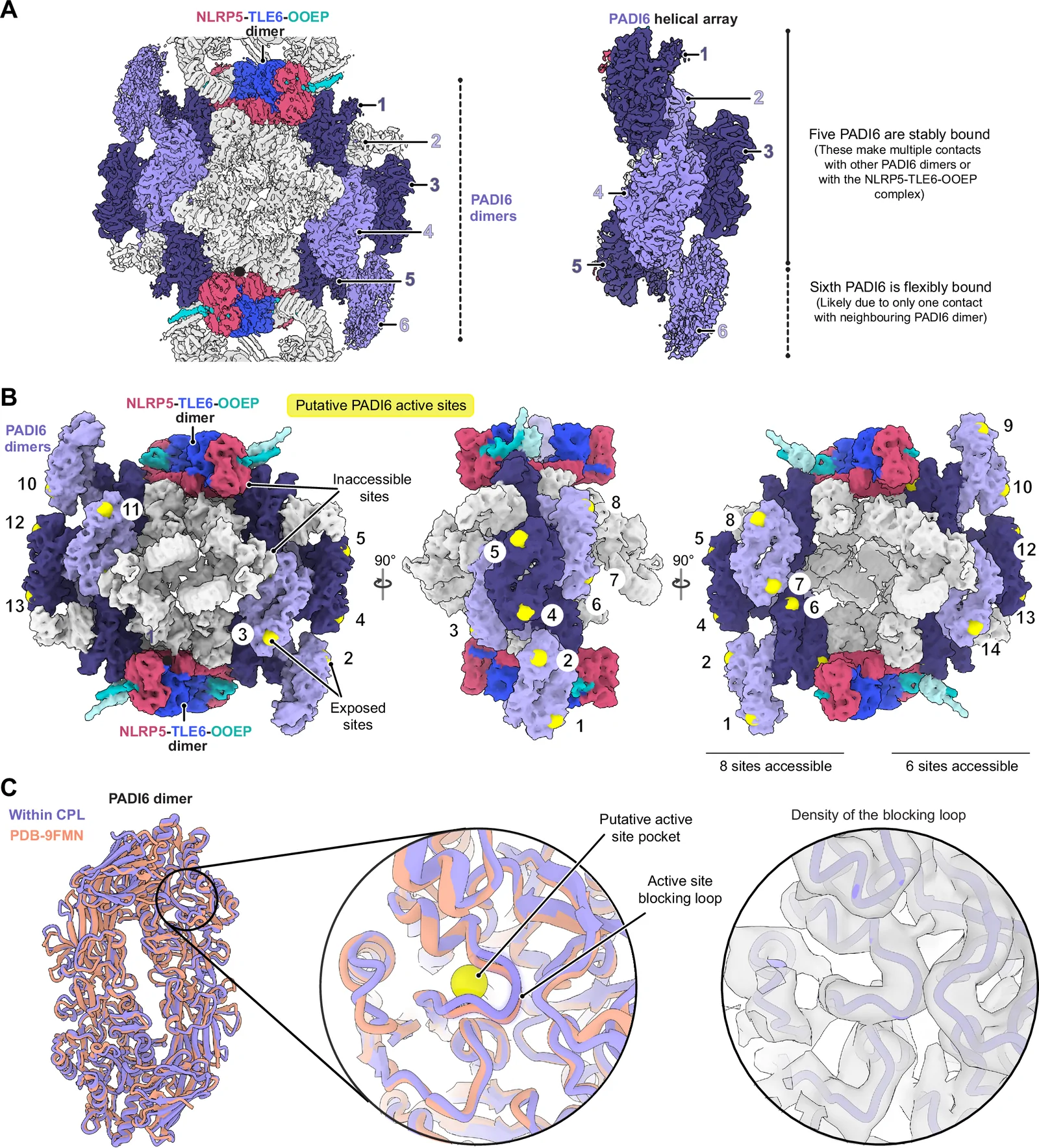
Arrangement of putative catalytic sites of PADI6 within CPLs. (**A**) Subtomogram averaging density of the PADI6 helical array is shown. Five PADI6 dimers (1–5) are stably bound, whereas the sixth PADI6 dimer is more flexibly attached as its corresponding density is resolved at a lower resolution. (**B**) Putative catalytic sites of PADI6 within a CPL repeat unit are depicted as yellow spheres. A total of 14 sites are exposed, whereas 10 sites are occluded by the interactions of PADI6 with the NLRP5–TLE6–OOEP complex or with the proteins bound to the central scaffold (shown in light gray). (**C**) The PADI6 dimer within the CPL helical array is compared with the structure of the isolated PADI6 dimer (PDB-9FMN) (Williams et al, 2024). In both cases, the putative catalytic pocket is occluded. The subtomogram averaging density corresponding to the blocking loop is shown on the right.

PADI6 is a member of the peptidylarginine deiminase (PADI) enzyme family, which catalyzes protein citrullination, and, like other PADI enzymes, forms homodimers (Ranaivoson et al, 2024; Williams et al, 2024; Williams and Walport, 2023). In our structure, PADI6 dimers assemble into higher-order helical arrays (Fig. 3B) that have not previously been described for proteins of the PADI family. Each helical array comprises six PADI6 dimers (Fig. 3A,B), five of which have well-defined density, whereas one is more flexible (Fig. EV5A). Two such arrays form the lateral sides of the CPL scaffold (Fig. 3A,B). PADI6 comprises two N-terminal immunoglobulin-like (Ig-like) subdomains and a C-terminal α/β-propeller domain (Fig. 3C), both of which mediate interactions that stabilize the helical assembly (Fig. 3D,E). The catalytic sites of PADI6, which face the cytoplasm in each CPL repeat, are occluded by neighboring loop segments (Fig. EV5B,C). This conformation is consistent with structures of isolated PADI6 dimers (Fig. EV5C) and biochemical data, which showed no detectable activity against canonical PADI substrates in vitro (Ranaivoson et al, 2024; Williams et al, 2024). Therefore, our data suggest that PADI6 adopts a conformation incompatible with citrullination in situ and instead serves a structural role within CPLs.

The NLRP5–TLE6–OOEP complex is incorporated as a dimer within the CPL repeat. Two such dimers are present per repeat unit, with each bridging the two lateral PADI6 arrays (Fig. 3A). Although previous in vitro studies detected both monomeric and dimeric forms (Chi et al, 2024), we observe only the dimeric complex, indicating that this is the functionally relevant state within CPLs. NLRP5 comprises an N-terminal NACHT domain and a C-terminal leucine-rich repeat (LRR) domain (Fig. 3C). The NACHT region engages TLE6 and OOEP, whereas the LRR domain mediates direct interactions with PADI6 (Fig. 3F). This arrangement establishes a direct structural link between PADI6 and the NLRP5–TLE6–OOEP complex. In contrast to previous proposals that these factors act as independent modules (Giaccari et al, 2024; Jentoft et al, 2023), our findings provide a structural explanation for the reported interactions between them and show that they assemble into a unified scaffold.

### SCMC proteins link CPL repeat units to form filaments

**Figure 4:**
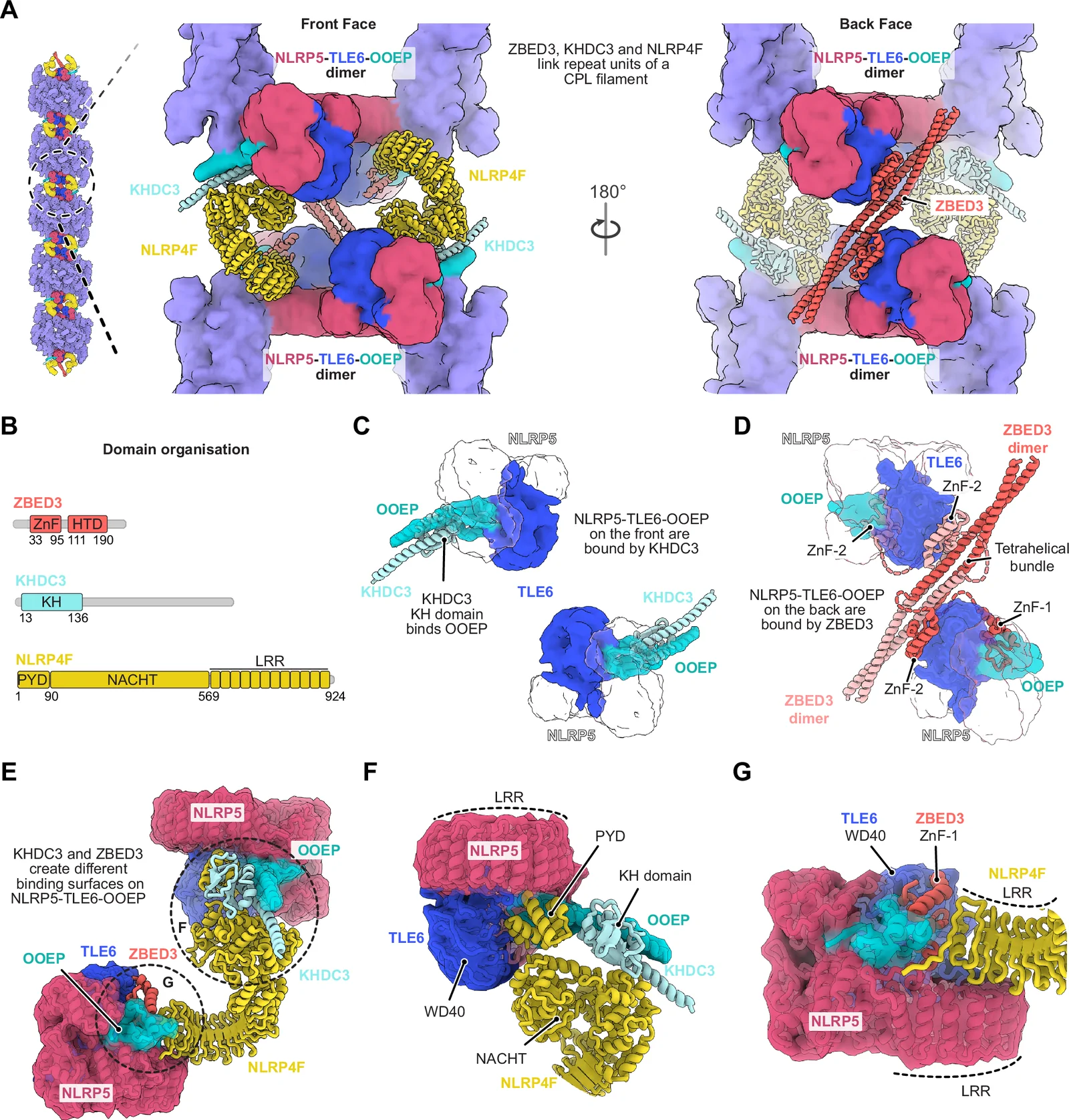
Molecular basis for the formation of CPL filaments. (**A**) Front (left) and back (right) faces of the segment between two CPL repeat units. KHDC3, ZBED3, and NLRP4F cooperate to link adjacent repeats. (**B**) Domain architecture of KHDC3, ZBED3, and NLRP4F. KH K-homology, ZnF BED type Zinc-finger, HTD helical tetramerization domain, PYD Pyrin domain, LRR leucine-rich repeat. (**C**, **D**) The interactions of KHDC3 (**C**) and ZBED3 (**D**) with the NLRP5–TLE6–OOEP complexes. The ZnF domain of ZBED3 that contacts both the KH domain of OOEP and the WD40 domain of TLE6 (labeled as ZnF-1) is stably bound, whereas the ZnF domain that interacts only with the TLE6 WD40 domain (labeled as ZnF-2 in (**D**)) is more flexibly associated (Fig. EV6). ZBED3 from adjacent repeats forms a four-helix bundle that links adjacent repeats. (**E**) Contacts made by NLRP4F while linking two adjacent repeat units. (**F**) Interaction of the N-terminal PYD and NACHT domain of NLRP4F with KHDC3. (**G**) Interaction of C-terminal LRR of NLRP4F with ZBED3-bound NLRP5–TLE6–OOEP complex.

**Figure EV6:**
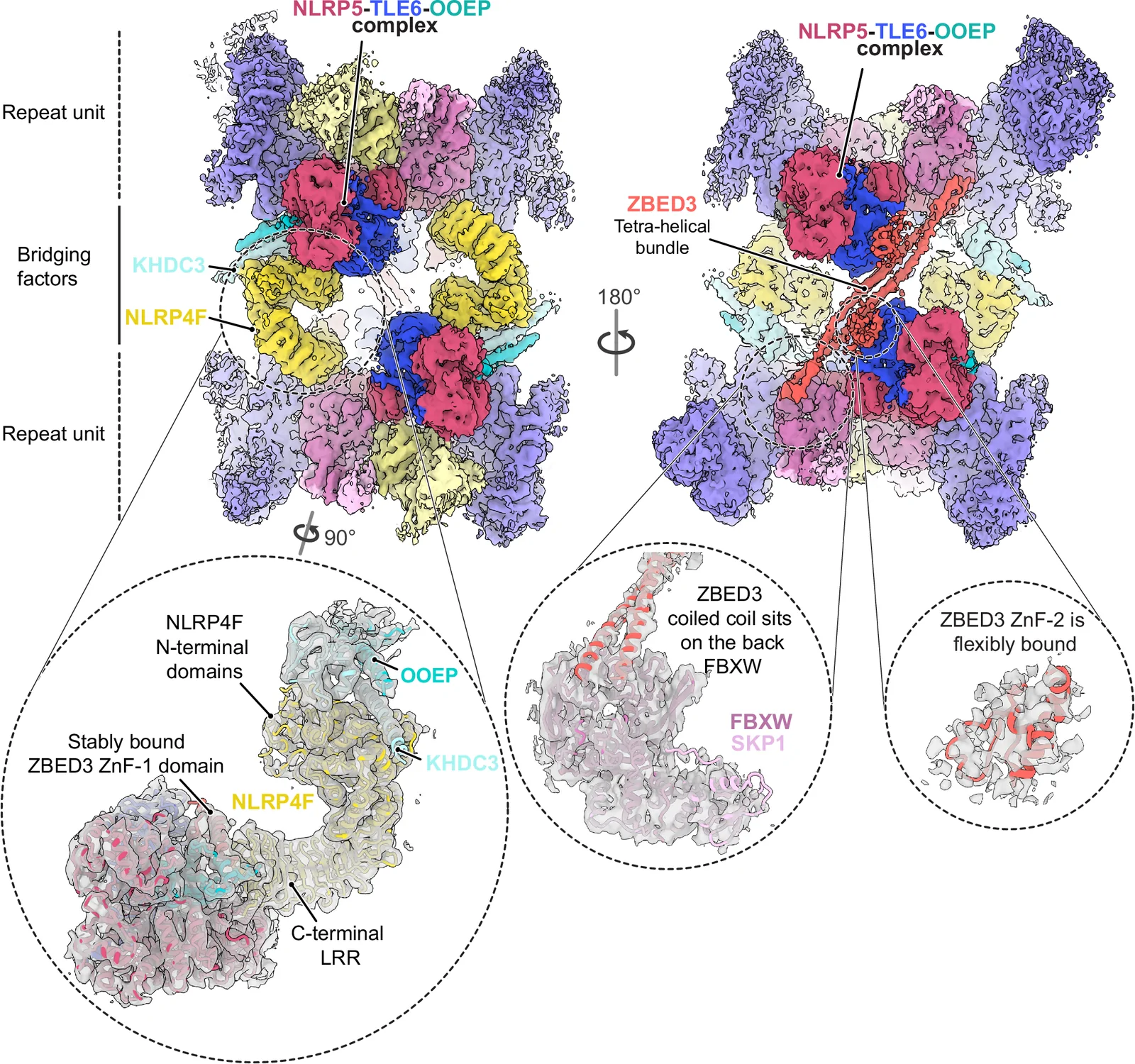
Subtomogram averaging density linking two CPL repeat units. Proteins mediating inter-repeat connections are labeled. Insets highlight NLRP4F, the interaction of the ZBED3 coiled-coil with a corner FBXW–SKP1 complex on the back face of the filament, and density corresponding to the flexibly attached ZBED3 zinc-finger (ZnF-2) domain bound to TLE6.

Adjacent CPL repeat units are linked by three bridging factors, KHDC3, ZBED3, and NLRP4F, which connect neighboring repeats through interactions with the NLRP5–TLE6–OOEP complexes (Figs. 4A and EV6). KHDC3 binds the front-facing side of each NLRP5–TLE6–OOEP dimer (Fig. 4B,C), whereas ZBED3 engages the opposite side on the back face of the filament (Fig. 4D). ZBED3 contains an N-terminal zinc-finger (ZnF) domain and a C-terminal helical region (Fig. 4B). The ZnF domains of two ZBED3 molecules bind TLE6. The C-terminal helical regions of two pairs of ZBED3 molecules form a tetrameric helical bundle that directly links neighboring repeats (Fig. 4D). Together, KHDC3 and ZBED3 create two distinct binding surfaces on the NLRP5–TLE6–OOEP dimer (Fig. 4E). The KHDC3-associated side contacts the N-terminal domains of NLRP4F (Fig. 4F), whereas the ZBED3-associated side binds the C-terminal LRR domain of NLRP4F (Fig. 4G). This leads to two NLRP4F molecules spanning neighboring repeat units in an antiparallel orientation (Fig. 4A).

Genetic data show that deletion of any of the three bridging factors results in the loss of CPLs (Gao et al, 2018; Qin et al, 2019; Zheng and Dean, 2009). The effect of *Khdc3* and *Zbed3* mutants is explained by their disruption of the binding sites for NLRP4F on the NLRP5–TLE6–OOEP dimers. The loss of CPLs observed in *Nlrp4f* mutants suggests that the connection mediated by ZBED3 alone can’t compensate for NLRP4F. Thus, all three bridging factors cooperate to link neighboring repeat units and promote CPL filament formation.

Overall, we show how different components of the SCMC interact within CPLs and define their stoichiometry. The SCMC was originally described based on enrichment of its components beneath the oocyte cortex (Li et al, 2008), in contrast to CPLs, which are distributed throughout the cytoplasm (Calarco and Brown, 1969; Enders and Schlafke, 1965; Schlafke and Enders, 1967). However, contrary observations (Jentoft et al, 2023; Tashiro et al, 2010) showing a uniform cytoplasmic distribution of SCMC components and their co-localization with CPLs have raised questions about the relationship between these two assemblies. Our data now provides direct evidence that the key components of the SCMC are part of the CPL filament.

### CPLs stably incorporate ubiquitination proteins and tubulin

**Figure 5:**
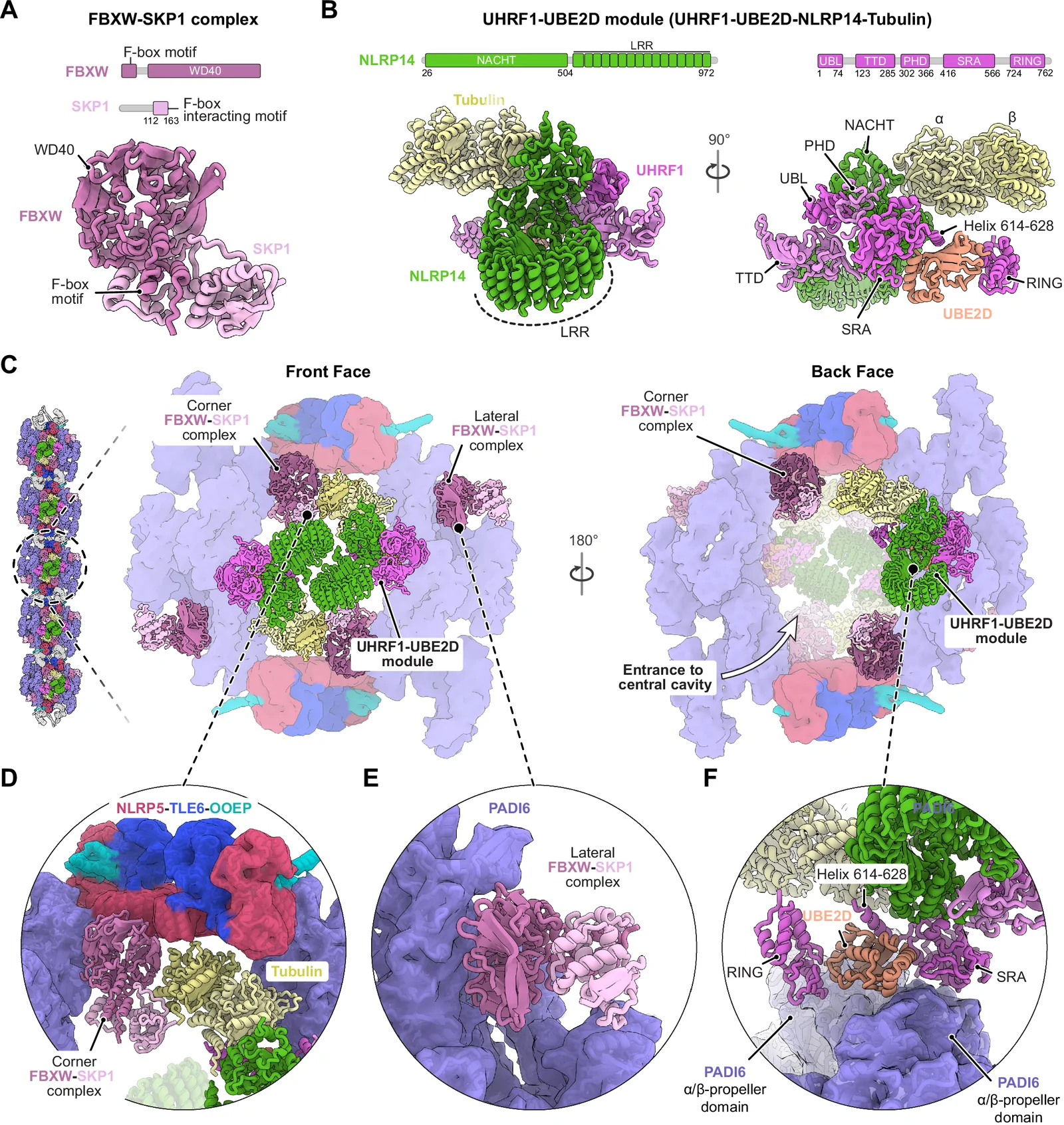
Organization of the proteins from the ubiquitination pathway within CPL repeat units. (**A**) Domain organization and structure of the FBXW–SKP1 complexes. (**B**) Domain organization and architecture of the UHRF1-UBE2D module composed of UHRF1-UBE2D-NLRP14-αβ-tubulin. (**C**) Arrangement of FBXW–SKP1 and UHRF1-UBE2D modules within a single CPL repeat unit, shown are front and back faces of the filament to highlight differences in protein composition and placement. (**D**–**F**) Zoom-in showing the interaction of the corner FBXW–SKP1 complex with tubulin of front UHRF1-UBE2D modules (**D**), binding of the lateral FBXW–SKP1 to the PADI6 helical array (**E**), and interaction of UHRF1 and UBE2D with PADI6 (**F**).

Our structure reveals that CPLs contain multiple FBXW–SKP1 complexes (Fig. 5A) together with three copies of a distinct assembly composed of UHRF1, UBE2D, NLRP14, and an αβ-tubulin heterodimer (Fig. 5B). These are positioned within the central region of the CPL repeat and complete the architecture of the repeat unit (Fig. 5C).

**Figure EV7:**
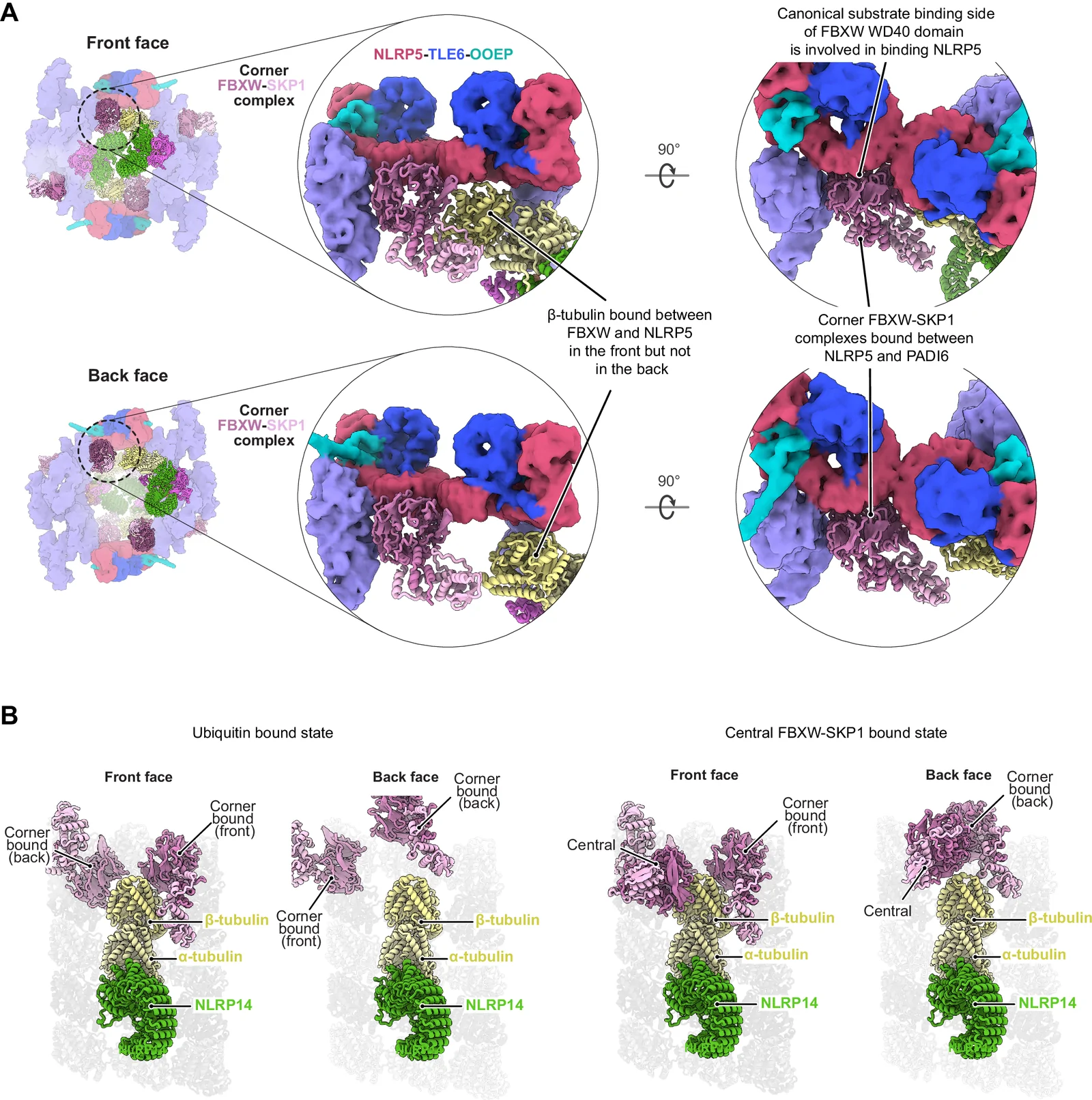
Interaction of FBXW–SKP1 complexes with UHRF1-UBE2D modules within the CPL repeat. (**A**) Models showing the interaction of corner FBXW–SKP1 complexes with the UHRF1-UBE2D modules on the front and back faces of the CPL repeat. On the front face, β-tubulin is positioned between FBXW–SKP1 and NLRP5, forming a compact interface that stabilizes the interaction with the UHRF1-UBE2D module. In contrast, on the back face, the interface is more open, and β-tubulin does not contact either component. Notably, the WD40 surface of FBXW–SKP1 that mediates substrate recognition in other F-box proteins is occluded through its interaction with NLRP5. (**B**) Interactions between αβ-tubulin-NLRP14 complexes on the front and back faces of the CPL repeat unit and FBXW–SKP1 complexes. The ubiquitin-bound state is shown on the left, and the state containing the central FBXW–SKP1 complex is shown on the right. To place these interactions in the structural context of a microtubule, the tubulin heterodimer is overlaid onto a microtubule lattice.

FBXW interacts with SKP1 via its F-box motif (Fig. 5A), consistent with other F-box-SKP1 assemblies (Rusnac and Zheng, 2020). Six of these complexes are anchored directly to the PADI6 and NLRP5–TLE6–OOEP scaffold (Fig. 5C). Four occupy corner positions between NLRP5 and PADI6 (Fig. 5D), while two bind along the outer surface of the PADI6 arrays (Fig. 5E). These interactions involve three distinct types of interfaces: the front-facing corner complexes contact β-tubulin (Figs. 5D and EV7A,B), the back-facing corner complexes contact ZBED3 (Fig. EV6), and the lateral complexes mediate the inter-filament interactions described later. Notably, all contacts (Fig. EV7A) involve the face of the WD40, which is used for substrate binding by other F-box proteins (Rusnac and Zheng, 2020).

Although FBXW–SKP1 complexes are substrate-receptor modules of SCF Cullin-RING ligases, their binding modes in CPLs are incompatible with this function. In addition to the canonical substrate-binding face of the F-box proteins binding the CPL, the CUL1-binding surface of SKP1 (Rusnac and Zheng, 2020) is either occluded or positioned such that CUL1 binding would clash with the surrounding CPL scaffold (Appendix Fig. S1A). Consistent with this, we do not observe density corresponding to CUL1. Thus, CPL-scaffold-associated FBXW–SKP1 complexes are unlikely to recruit CUL1-containing Cullin-RING ligases and instead appear to mediate interactions with other CPL components and contribute to the higher-order assembly of CPL filaments.

The UHRF1-containing assembly, hereafter referred to as the UHRF1-UBE2D module, is organized around NLRP14 (Fig. 5B). Within this module, NLRP14 simultaneously binds α-tubulin and engages multiple N-terminal domains of UHRF1, namely the ubiquitin-like (UBL), tandem Tudor (TTD), plant homeodomain (PHD), and SET- and RING-associated (SRA) domains. UBE2D, in turn, interacts with three UHRF1 regions: the SRA domain, a helix comprising residues 614-628 that contacts the backside of UBE2D, and the C-terminal RING domain (Fig. 5B). This differs from a recent structure of a reconstituted human PADI6-UHRF1-UBE2D3 complex (Li et al, 2026a), in which UBE2D was bound only by the SRA domain and residues 614-628, with no RING domain interaction observed. Within the CPL repeat, we observe three UHRF1-UBE2D modules. Two are positioned symmetrically on the front face. These are stabilized by contacts between the UHRF1 SRA domain and PADI6 (Fig. 5F), and by interactions between the corner FBXW–SKP1 complexes and β-tubulin (Fig. 5D). The third module occupies the back face, where it is anchored primarily through contacts between the UHRF1 SRA domain and PADI6 (Fig. 5F).

**Figure 6:**
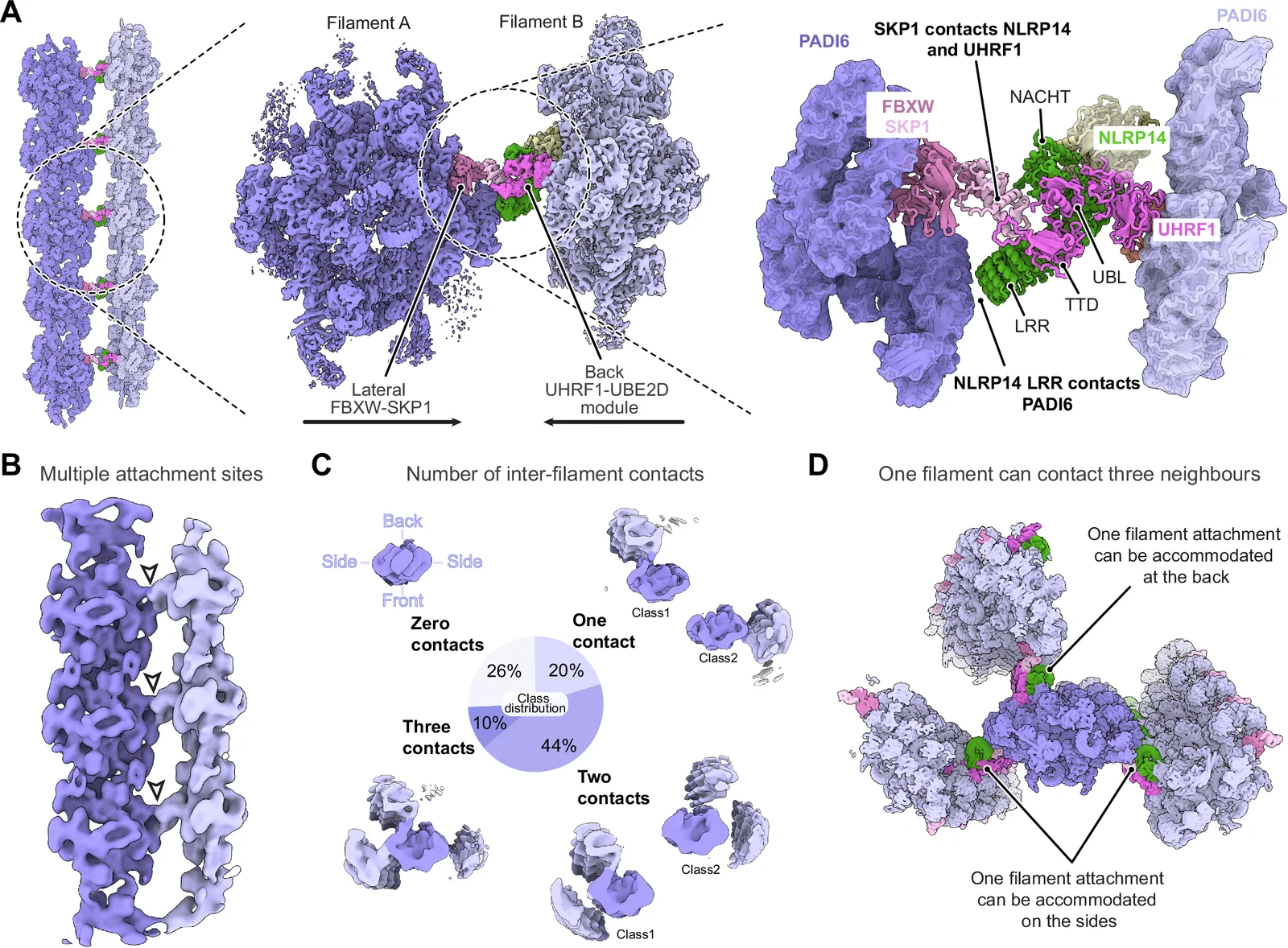
Molecular basis for organization of CPL filaments into lattices. (**A**) Inter-filament linkages mediated by the FBXW–SKP1 and PADI6 on the side of one filament contacting the UHRF1-UBE2D modules at the back face of an adjacent filament. (**B**) Multiple adjacent inter-filament attachment points are shown along the inter-filament interface. (**C**) Distribution of 3D classes showing the number of stable inter-filament contacts observed within CPLs. Top views of the representative classes are displayed. (**D**) The three sites that can be employed for inter-filament contacts are depicted.

**Figure EV8:**
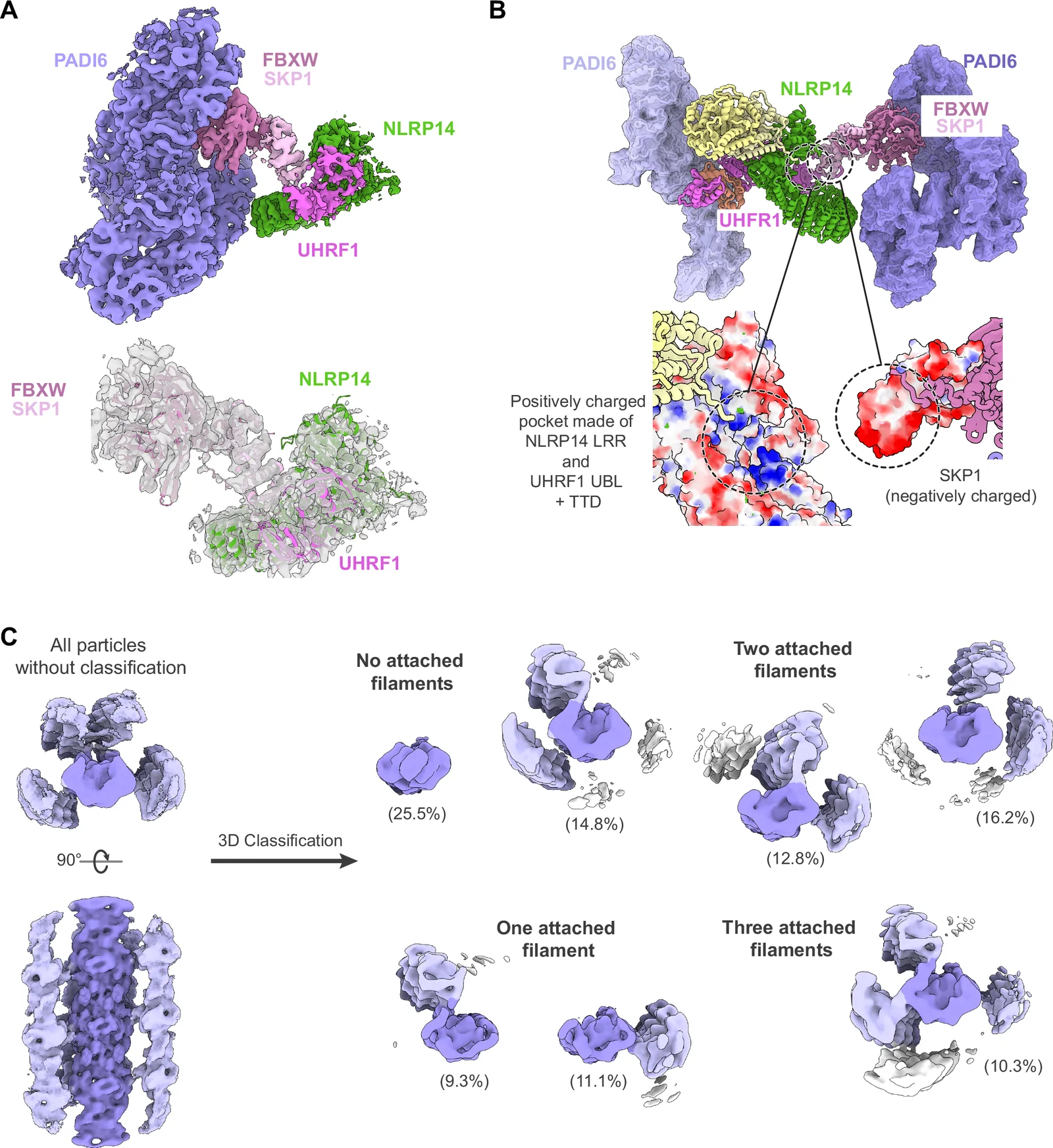
Inter-filament contacts within CPLs. (**A**) Subtomogram averaging density of the proteins linking two CPL filaments (top) with the corresponding atomic model of FBXW–SKP1, NLRP14, and UHRF1 fitted into the density (bottom). (**B**) View rotated 180° relative to Fig. 6A showing that SKP1 engages the neighboring CPL filament through a negatively charged patch (red) that binds a positively charged pocket (blue) formed by the NLRP14 LRR and the UHRF1 UBL and TTD domains. White color denotes neutral charge. (**C**) Reconstruction of all particles corresponding to CPL repeats at a box size of 1528 Å prior to 3D classification (left) and the resulting 3D classes after classification, with the percentage of particles assigned to each class indicated in brackets.

The FBXW–SKP1 complexes and UHRF1-UBE2D modules not only form integral components of the repeat unit but also mediate higher-order organization of filaments into lattices. A lateral FBXW–SKP1 and a nearby PADI6 on one filament engage NLRP14 and UHRF1 on the back face of a neighboring filament (Figs. 6A and EV8A,B). Multiple copies of these interactions cooperate to generate stable inter-filament linkages (Fig. 6B). To define how these contacts give rise to lattice organization, we performed 3D classification using a larger particle box that included neighboring filaments (Fig. EV8C). This analysis showed that lattice assembly is heterogeneous, spanning a range of attachment patterns from filaments with no stably attached neighbors to filaments engaged with up to three neighbors (Figs. 6C and EV8C). These contacts occur at defined attachment points, with each filament forming up to two lateral interactions and one back-face interaction (Fig. 6D).

### CPL repeats contain density consistent with ubiquitin charged UBE2D

**Figure EV9:**
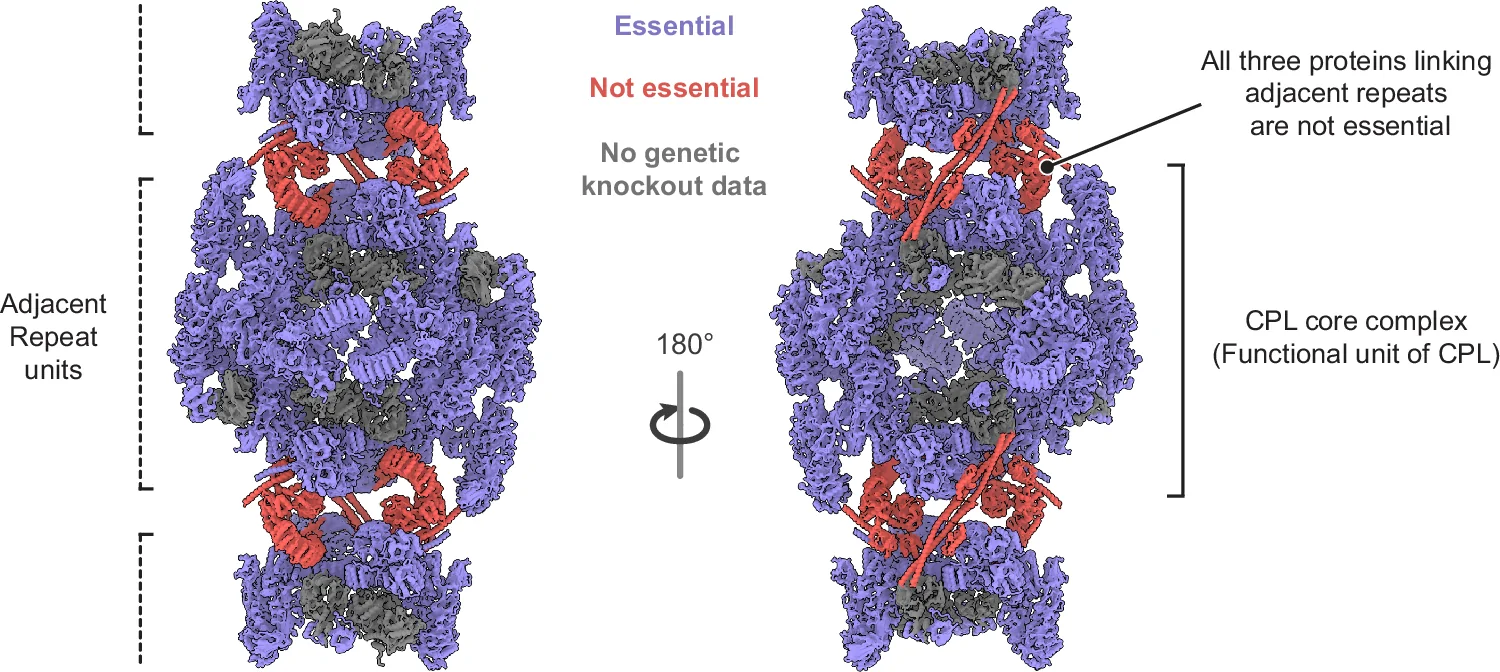
Mapping of genetic loss-of-function phenotypes onto the CPL structure. Proteins identified in the CPL repeat are colored according to the developmental phenotypes reported upon gene disruption in mice. Proteins whose loss results in embryonic lethality or infertility are shown in lavender, proteins whose deletion permits viable progeny are shown in orange, and proteins for which genetic data are currently unavailable are shown in gray. This mapping highlights that factors essential for development form the CPL scaffold or reside within the CPL core complex, whereas proteins required for higher-order filament assembly are dispensable for viability.

The importance of the CPL constituents identified in the structure and their higher-order assembly is indicated by genetic loss-of-function studies (Gao et al, 2018; Li et al, 2008; Qin et al, 2019; Tashiro et al, 2010; Uemura et al, 2023; Yu et al, 2014; Yurttas et al, 2008; Zhang et al, 2023; Zheng and Dean, 2009). We find proteins whose absence results in embryonic lethality or infertility comprise the CPL scaffold (PADI6 and NLRP5–TLE6–OOEP) or reside within it (UHRF1, UBE2D, NLRP14, and SKP1). In contrast, deletion of bridging factors required for filament assembly (KHDC3, ZBED3, and NLRP4F) permits viable progeny despite CPL loss. Together, these findings demonstrate that filament formation itself is not essential for development. Instead, it is the scaffold and assemblies that bind it that form the functional unit of CPLs required for developmental success. We therefore refer to this functional unit as the CPL core complex (Fig. EV9).

**Figure 7:**
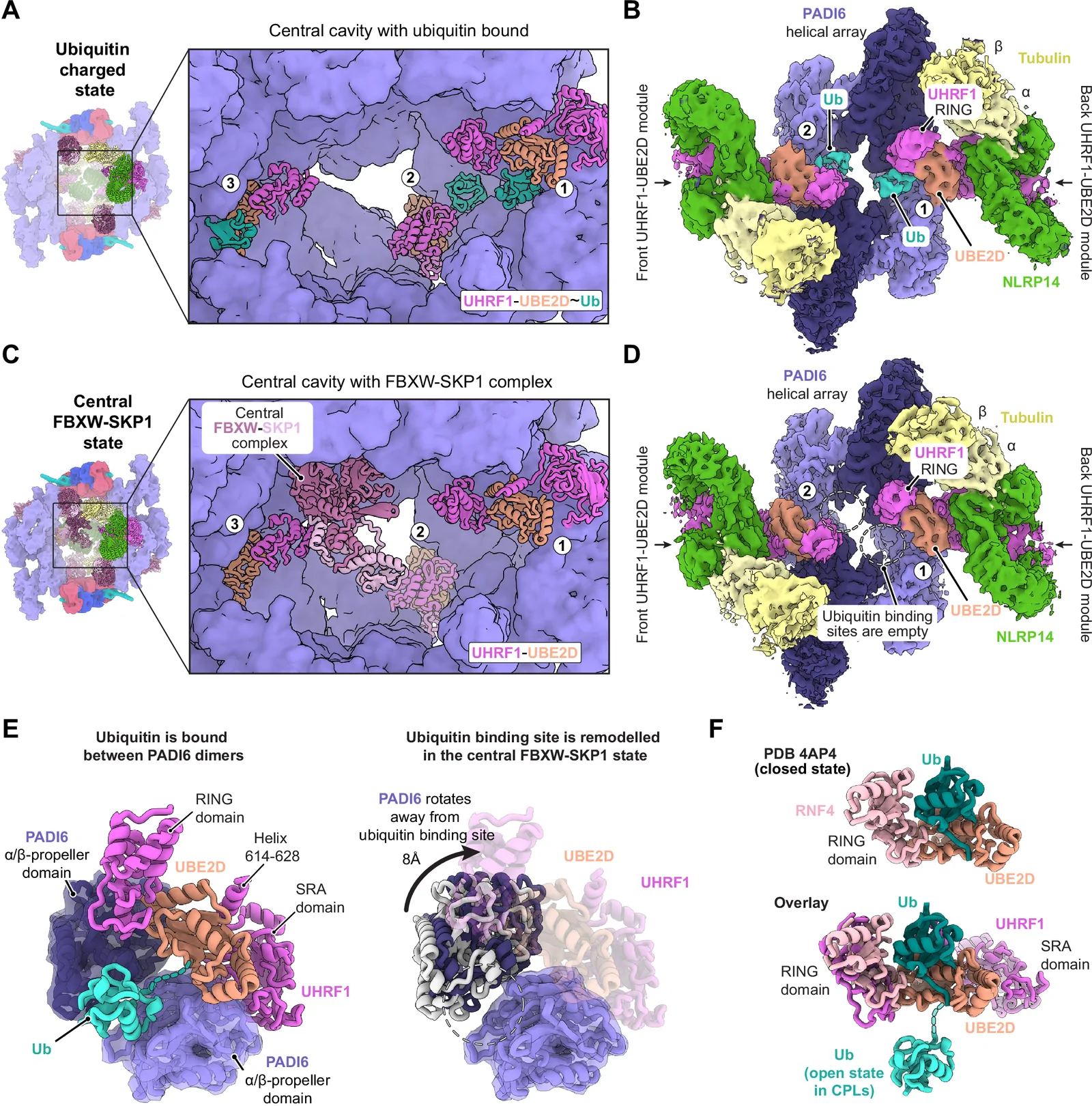
The central cavity within CPL repeats contains ubiquitin-charged UBE2D. (**A**) Central cavity within a CPL repeat unit viewed from the back face, showing the arrangement of the three UHRF1-UBE2D (RING-E2) modules and bound ubiquitin moieties. (**B**) Subtomogram averaging density corresponding to the UHRF1-UBE2D modules labeled 1 and 2 in (**A**). (**C**) Central cavity within a CPL repeat unit in the state containing a central FBXW–SKP1 complex. (**D**) Subtomogram averaging density corresponding to the UHRF1-UBE2D modules labeled 1 and 2 in (**C**) in the presence of the additional FBXW–SKP1 complex within the cavity. (**E**) Comparison of the ubiquitin-bound configuration and the central FBXW–SKP1 state. In the ubiquitin-charged state, ubiquitin is stabilized between the α/β-propeller domains of two PADI6 dimers. In the FBXW–SKP1 state, rearrangements of the PADI6 helical array open the ubiquitin-binding pocket. (**F**) Structure of UBE2D bound to a RING domain with ubiquitin in the closed conformation (PDB-4AP4 (Plechanovova et al, 2012)). In this configuration, ubiquitin is positioned between the RING domain and the E2 enzyme, representing the catalytically competent state. Overlay with the UHRF1-UBE2D-ubiquitin configuration observed in CPLs shows that ubiquitin adopts an open conformation relative to the closed active state.

A distinctive feature of the CPL core complex is the arrangement of UHRF1-UBE2D modules, which generates a large central cavity (~2000 nm³) within each repeat (Fig. 5C). This cavity contains all three UBE2D molecules (Fig. 7A) and remains accessible to cytoplasmic proteins through an ~15 nm opening on the back face of the filament.

**Figure EV10:**
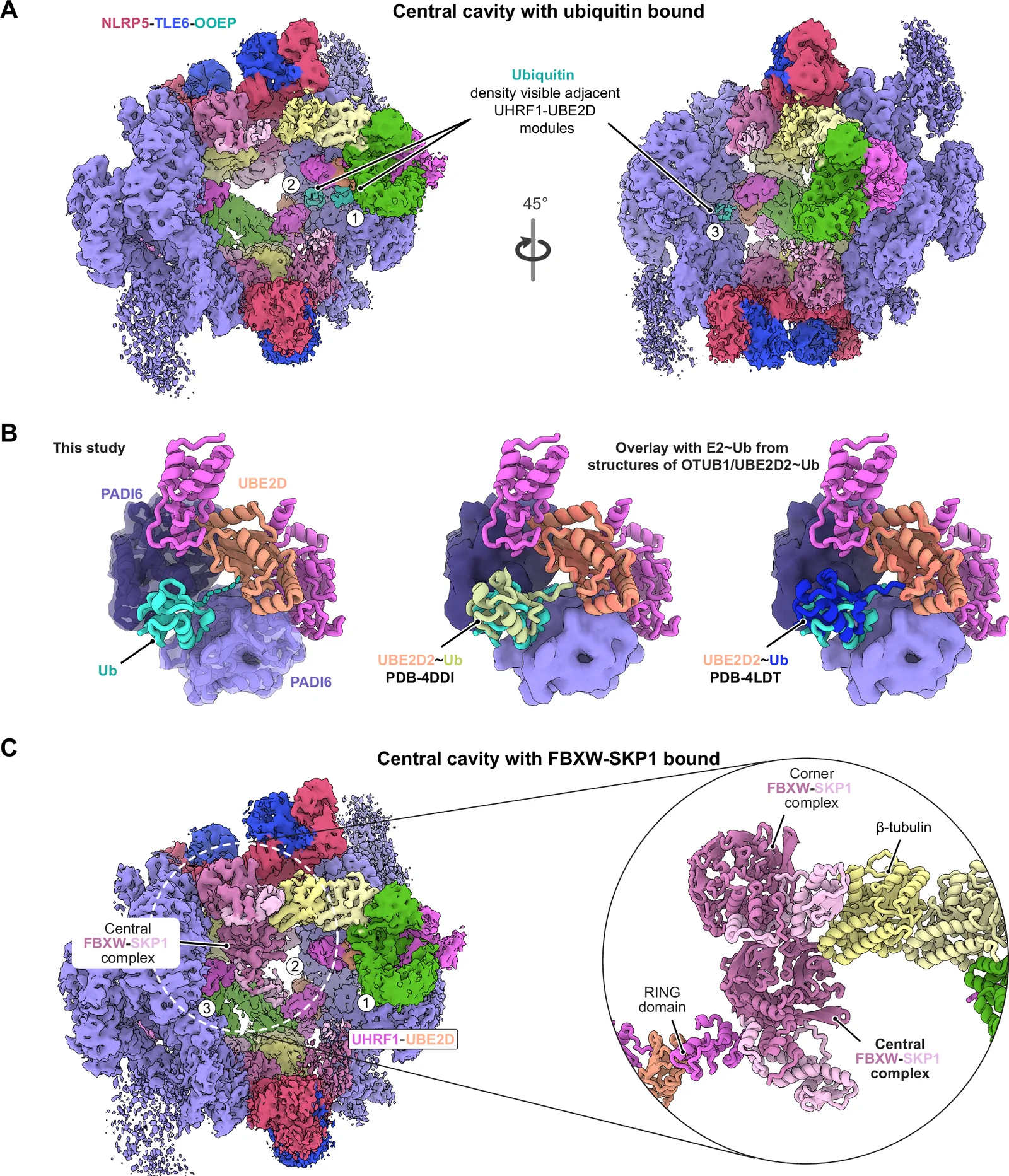
Structural states of the CPL core complex. (**A**) Subtomogram averaging density of the CPL repeat unit in ubiquitin-bound state showing ubiquitin density (teal) adjacent to the UHRF1-UBE2D modules within the central cavity, consistent with ubiquitin conjugated to UBE2D. (**B**) Comparison of the open E2~Ub state within CPLs with those observed in OTUB1-bound E2~Ub structures (PDB-4DDI (Juang et al, 2012) and PDB-4LDT (Wiener et al, 2013)). The E2~Ub within CPLs adopts an open conformation closely resembling that within the OTUB1-bound E2~Ub structures. (**C**) Density of the CPL repeat unit in the state containing an additional central FBXW–SKP1 complex and lacking detectable ubiquitin density. The inset shows a close-up of the central FBXW–SKP1 complex, which contacts β-tubulin of the back-face complex and lies adjacent to the RING domain of UHRF1.

Using 3D classification, we resolved two distinct structural states of this cavity, accounting for 37.8% and 44.4% of particles, respectively; the remaining 17.8% fell into poorly resolved classes and could not be assigned to either state (Figs. 7A–D and EV3 and see “Methods”). In the first state, density consistent with ubiquitin (Ub) is observed adjacent to UBE2D molecules (Figs. 7B and EV10A). The distance between the ubiquitin and the UBE2D catalytic cysteine is consistent with a covalently linked E2~Ub conjugate (Baek et al, 2020; Branigan et al, 2015; Dou et al, 2012). The ubiquitin moiety is lodged between the α/β-propeller domains of two PADI6 dimers (Fig. 7E), revealing an unexpected role for PADI6 in stabilizing ubiquitin bound to an E2 enzyme. In this configuration, the E2~Ub is held in an open conformation similar to that observed in structures of an E2~Ub bound to OTUB1 (Juang et al, 2012; Wiener et al, 2013) (Fig. EV10B). This conformation is associated with low activity for ubiquitin transfer (Pruneda et al, 2012). It contrasts with the catalytically active closed E2~Ub state in which ubiquitin engages both the E2 and the RING domain, holding the thioester bond in a conformation competent for transfer (Baek et al, 2020; Branigan et al, 2015; Dou et al, 2012) (Fig. 7F).

In the second state, the cavity contains an additional centrally positioned FBXW–SKP1 complex, which is bound to a corner FBXW–SKP1 complex and to the β-tubulin of the back face UHRF1-UBE2D module (Figs. 7C, EV7B, and EV10C). Strikingly, this state has no stable ubiquitin density present adjacent to the UBE2D subunits (Fig. 7D). Structural comparison with the state in which ubiquitin density is present adjacent to the UBE2D molecules shows that the presence of the central FBXW–SKP1 complex is accompanied by rearrangements within the CPL core complex. The back UHRF1-UBE2D module moves inwards so that its tubulin contacts the central FBXW–SKP1. This movement is coupled to a rearrangement of the PADI6 helical array, leading to an opening of the ubiquitin-binding pocket (Fig. 7E; Movie EV2).

Together, our data reveal that the CPL core complex can adopt two distinct states. In one, the ubiquitin moiety is stabilized by PADI6 in an open E2~Ub conformation, which is expected to restrain engagement of ubiquitin with the UHRF1 RING domain. In the second state, the shift of the PADI6 dimer opens the ubiquitin-binding pocket. This could allow ubiquitin to become more flexible while remaining tethered to UBE2D, providing an explanation for why its density is no longer resolved. Alternatively, the absence of ubiquitin density could reflect loss of E2~Ub conjugate or transfer of ubiquitin to an unknown acceptor. Thus, while our structural data do not directly demonstrate ubiquitin transfer within CPLs, they show that CPLs can hold ubiquitin-charged UBE2D in a restrained open state and can also adopt a conformation in which this restraint is absent, allowing ubiquitin to sample conformations more compatible with transfer onto a substrate.

## Discussion

This study provides a strategy for in situ structural analysis of mammalian embryos at resolutions sufficient to identify proteins and visualize different states of protein complexes. By separating 6/8-cell stage mouse embryos into individual blastomeres before vitrification, we generated samples compatible with automated cryo-FIB milling and obtained 6.6 Å reconstructions using only 14 embryos. These low material requirements make the approach suited for scarce samples such as human embryos donated for research. Although demonstrated here at the 6/8-cell stage, the workflow is applicable to other stages of preimplantation development, allowing structural investigation of macromolecular assemblies in early mammalian embryos.

Using this approach, we resolved the structure of CPLs in situ and identified the components that form the repeating unit of CPL filaments and mediate their assembly into higher-order lattices. Our structure shows that each CPL repeat is built around a PADI6 and NLRP5–TLE6–OOEP scaffold, which recruits additional SCMC components, FBXW–SKP1 complexes, and UHRF1-UBE2D module complexes. Together, these components generate a pseudo-symmetric repeat unit containing a central cavity with three UHRF1-UBE2D modules.

During the revision of this manuscript, five single-particle cryo-EM studies reported structures of CPLs extracted from mouse oocytes (Chi et al, 2026; Kilic et al, 2026; Li et al, 2026b; Liu et al, 2026; Zhang et al, 2026). These studies identify the same broad set of components as constituents of CPL repeat units. However, there are many differences in the composition, symmetry, and organization of the reported structures. For example, Liu et al (Liu et al, 2026) do not resolve the back-face UHRF1-UBE2D module or FBXW–SKP1 subunits, whereas Kilic et al (Kilic et al, 2026) resolve the back-face FBXW but not the associated SKP1 or UHRF1-UBE2D module. These missing components may reflect partial disruption during CPL extraction or cryo-EM sample preparation. In contrast, Zhang et al and Li et al (Zhang et al, 2026; Li et al, 2026b) describe CPL repeats as fully symmetric, with the back face also containing two UHRF1-UBE2D modules. Our asymmetric 3D reconstructions instead show that native CPLs are pseudo-symmetric, suggesting that the higher copy number of the UHRF1-UBE2D modules in these two studies likely arises from imposing or assuming C2 symmetry. Consistent with our observations, (Chi et al, 2026) also report a pseudo-symmetric architecture in which only one UHRF1-UBE2D module is present on the back face. The single-particle studies nevertheless provide complementary information on the identity of the FBXW proteins. Whereas the resolution of most structures, including ours, was not sufficient to identify or discriminate between closely related FBXW family members based on the density maps, Chi et al and Li et al (Chi et al, 2026; Li et al, 2026b) had sufficient resolution to assign three FBXW–SKP1 complexes to specific isoforms: the front-face complexes as FBXW18, the back-face complexes as FBXW19, and the central cavity complex as FBXW21.

Our in situ structure reveals additional components and lattice-related features that are not resolved in the structures solved from extracted CPLs. We identify a four-helix bundle, which we assign to the ZBED3 C-terminal helix, that contributes to inter-repeat attachment within a filament (Fig. 4A). This indicates that four ZBED3 molecules are present per repeating unit, in contrast to the two copies resolved in other studies. We further resolve inter-filament attachments and show that these are mediated by an additional FBXW–SKP1 complex, which binds PADI6 on one filament and contacts UHRF1-NLRP14 on the back face of an adjacent filament (Fig. 6A). Given its distinct binding mode to PADI6, we speculate that this FBXW–SKP1 complex represents a fourth FBXW isoform, although its precise identity remains to be determined. We also find that the lattice arrangement of CPL filaments is driven by a heterogeneous mixture of inter-filament contacts, with each filament making between zero and three stable attachments (Fig. 6C). This contrasts with other studies, in which each filament is reported to contact four neighbors. That arrangement resembles the mixed, unclassified population we observed before 3D classification (Fig. EV8C), suggesting that the four-neighbor arrangement represents an average of multiple states rather than the native architecture. Most significantly, we observe that 37.8% of particles contain density consistent with UBE2D charged with ubiquitin in an open state (Fig. 7A,B). The ubiquitin-charged state was not observed in any of the CPLs extracted from oocytes, which may reflect disruption of this labile state during CPL extraction. Alternatively, it may indicate that ubiquitin charging of CPL-associated UBE2D is developmentally regulated and becomes more prominent in the embryo. Determining whether the ubiquitin-charged state is also present in oocytes, or whether it emerges specifically during embryonic development, will require future in situ structural analysis across defined developmental stages. Overall, while structures of extracted CPLs provide important insight into their composition, the differences among these reconstructions, together with the apparent loss of subunits and higher-order organization during CPL extraction, highlight the importance of analyzing CPLs in their native cellular context.

Despite occupying 10% of the cytoplasmic volume of early embryos (Jentoft et al, 2023), the function of CPLs is currently unclear. One proposed role is protein storage (Jentoft et al, 2023; PREPRINT: Williams et al, 2026). However, all density in our subtomogram averages can be accounted for by the assigned proteins, raising the question of whether and how the hundreds of maternal proteins proposed to be stored on CPLs associate with the filament. For some candidates, such as 14-3-3 proteins and the SCMC interactor NLRP2, binding within the lattice appears unlikely as their reported binding sites on the NLRP5–TLE6–OOEP complex (Ou et al, 2025) are occluded in the CPL structure (Appendix Fig. S1B,C). Other factors may be associated flexibly or sparsely and would therefore not be resolved in our averaged electron density. One potential site for such interaction is the disordered C-terminal region of KHDC3, which is known to bind SPIN1 (Xu et al, 2025). Another way in which CPLs could act as storage sites is by specifically sequestering tubulin and components of the ubiquitination machinery, namely UHRF1, UBE2D, and the FBXW–SKP1 complexes. Our structure shows that these proteins remain integral components of CPLs until the 6/8-cell stage, and proteomic data (Zhu et al, 2025) indicate that CPL components are stably present until the morula stage and remain abundant in blastocysts (Appendix Fig. S1D). Large-scale CPL disassembly is therefore likely to occur after the 8-cell stage, and potentially during the later stages of preimplantation development. Once released, tubulin could contribute to the microtubule cytoskeleton, and UHRF1 and UBE2D could function in DNA methylation (Bostick et al, 2007; Nishiyama et al, 2013) and ubiquitination (Parashar et al, 2025; Stewart et al, 2016), and CPL-associated FBXW–SKP1 could be involved in substrate recruitment to CRL ubiquitin ligases. Thus, while CPLs may act as storage sites, our structure suggests that only a limited and defined set of proteins is stably incorporated within these structures.

In contrast to a purely storage role, our structure raises the possibility that CPLs are directly involved in ubiquitination. The UBE2D molecules within a CPL repeat unit appear to be able to exist in a ubiquitin-charged state and are bound by the RING domains of UHRF1. This argues against previous proposals that UHRF1-UBE2D modules within CPLs are inactive due to the inability to accommodate a ubiquitin-charged E2 (Li et al, 2026a; Li et al, 2026b). A second argument for inactivity has been that the UBL domain of UHRF1, which normally binds the backside of the E2 (DaRosa et al, 2018; Foster et al, 2018), is instead bound to NLRP14 within CPLs (Chi et al, 2026; Li et al, 2026b; Liu et al, 2026; Zhang et al, 2026). Despite the UBL being unavailable, we find that UBE2D is still recruited by UHRF1 through the SRA domain, a helix that contacts the backside surface of the E2, and the RING domain (Fig. 7E). Comparable backside interactions with a helix promote monoubiquitination by other RING E3 ligases (Hibbert et al, 2011; Li et al, 2015) (Appendix Fig. S1E), so this binding mode remains compatible with monoubiquitination, the type of modification UHRF1 is known to catalyze (Nishiyama et al, 2020; Nishiyama et al, 2013). We further find that the potential UBE2D~Ub conjugates correspond to a catalytically unfavorable open conformation stabilized through interactions between ubiquitin and PADI6. They are thus limited in their ability to transfer ubiquitin onto non-specific targets. Strikingly, when the central FBXW–SKP1 complex is present, the ubiquitin-binding pocket made by PADI6 is open, and the ubiquitin density is no longer resolved. Without a stable binding pocket on PADI6, ubiquitin would be free to interact with the UHRF1 RING domain bound to UBE2D and could adopt the catalytically active closed E2~Ub conformation. Together, the architecture of CPLs indicates they possess the important features required for ubiquitin transfer: ubiquitin-charged E2 enzymes, a mechanism for regulating ubiquitin availability, and RING domains positioned to promote the catalytically active state.

How substrates would be recognized within CPLs is not immediately apparent from our structure. The chromatin-binding surfaces of the TTD, PHD, and SRA domains that UHRF1 uses to engage its canonical nuclear substrates (Avvakumov et al, 2008; Shikimachi et al, 2025) are occluded by NLRP14 or PADI6. The UBL domain, which contributes to substrate selectivity for histone H3 (DaRosa et al, 2018), is similarly unavailable through its interaction with NLRP14. The sequestration of these canonical recognition modules is consistent with the cytoplasmic localization of UHRF1 within CPLs (Maenohara et al, 2017; Uemura et al, 2023), where it would not encounter its conventional nuclear substrates. The loss of these canonical recognition modules might suggest that UHRF1 is simply inactive, as proposed by other studies (Chi et al, 2026; Li et al, 2026b; Liu et al, 2026; Zhang et al, 2026). However, as described above, the CPL architecture retains the features required for ubiquitin transfer, suggesting that UHRF1 may remain active. In that case, its substrates must be selected by other means. The unexpected role of the CPL scaffold in binding ubiquitin raises the possibility that the scaffold could participate in substrate recruitment through as-yet-unidentified interactions. We also find that the ubiquitin density is absent when the central FBXW–SKP1 complex is present, suggesting that this complex could serve as a substrate-recruiting component. Notably, the canonical substrate-binding surface on the FBXW WD40 domain (Rusnac and Zheng, 2020) is engaged with the CPL scaffold, so any substrate would need to be recognized on the side surface of the WD40, in a manner analogous to the recognition of D-box and ABBA motifs by the WD40 domains of the APC/C coactivators Cdc20 and Cdh1 (Di Fiore et al, 2015; He et al, 2013). Because ubiquitin-dependent regulation can influence protein activity, localization, and stability (Komander and Rape, 2012; Swatek and Komander, 2016), CPLs acting as sites of ubiquitination would also help explain the diverse defects observed upon CPL loss.

Together, our observations support a hypothesis that CPLs function as large E2-E3 ubiquitin ligase assemblies directly involved in ubiquitination, although this model remains to be directly tested. Future experiments will be required to determine whether ubiquitin transfer occurs within CPLs, establish whether this activity differs between oocytes and embryos, identify physiological substrates, and define how CPL-associated ubiquitination contributes to early developmental progression.

## Methods

**Table:** Reagents and tools table

Reagent/resource	Reference or source	Identifier or catalog number
**Experimental models**		
Mouse: CD-1 (timed-mated females, ~8–12 weeks old)	Charles River	
**Antibodies**		
Rat monoclonal anti-SOX2 (1:200)	Invitrogen	Cat #14-9811-82
Rabbit monoclonal anti-GATA3 (1:200)	Abcam	Cat #ab199428
Donkey anti-Rabbit IgG (H + L), Alexa Fluor 488 (1:400)	Invitrogen	Cat #A-21206
Donkey anti-Rat IgG (H + L), Alexa Fluor Plus 555 (1:400)	Invitrogen	Cat #A48270
**Chemicals, enzymes, and other reagents**		
Hyaluronidase	Sigma-Aldrich	Cat #H4272
EmbryoMAX Advanced KSOM medium	Millipore	Cat #MR-101-D
Acidic Tyrode’s solution	Sigma-Aldrich	Cat #T1788
Trypsin (2.5%)	Gibco	Cat #15090046
EDTA (0.5 M, pH 8.0)	Invitrogen	Cat #15575-038
Bovine serum albumin (BSA, 30%)	Sigma-Aldrich	Cat #A7284
Bovine serum albumin (BSA)	Fisher BioReagents	Cat #BP1605-100
Methanol-free formaldehyde (16%)	Thermo Scientific	Cat #28906
Triton X-100	Sigma-Aldrich	Cat #93443-100 ML
VECTASHIELD mounting medium with DAPI	Vector Laboratories	Cat #H-1200
Polyvinylpyrrolidone (PVP)	Sigma-Aldrich	Cat #P0930
Dimethyl sulfoxide (DMSO)	Sigma-Aldrich	Cat #D2653-5x5ML
Ethylene glycol	Sigma-Aldrich	Cat #324558-100 ML
Phosphate-buffered saline (PBS)	Sigma-Aldrich	Cat #D8537-500ML
Paraffin/mineral oil	SAGE	Cat #ART-4008-5P
**Software**		
RELION-5	https://relion.readthedocs.io/en/release-5.0/#	
MotionCor2	https://emcore.ucsf.edu/ucsf-software	
CTFFIND4.1	https://grigoriefflab.umassmed.edu/ctf_estimation_ctffind_ctftilt	
AreTomo2	https://github.com/czimaginginstitute/AreTomo2	
PyTOM	https://github.com/SBC-Utrecht/pytom-match-pick	
cryoCARE	https://github.com/juglab/cryoCARE_pip/releases	
MemBrain v2	https://github.com/CellArchLab/MemBrain-v2	
UCSF ChimeraX	https://www.cgl.ucsf.edu/chimerax/	
ArtiaX	https://github.com/FrangakisLab/ArtiaX	
ISOLDE	https://tristanic.github.io/isolde/	
Coot	https://www2.mrc-lmb.cam.ac.uk/personal/pemsley/coot/	
PHENIX	https://phenix-online.org/download	
AlphaFold3	https://alphafoldserver.com/	
Maps software	Thermo Fisher Scientific	
AutoTEM Cryo	Thermo Fisher Scientific	
Tomography 5 software	Thermo Fisher Scientific	
**Other**		
Aquilos 2 cryo-FIB-SEM	Thermo Fisher Scientific	
Titan Krios G3i TEM (300 kV)	Thermo Fisher Scientific	
Titan Krios G4 TEM (300 kV)	Thermo Fisher Scientific	
K3 direct electron detector + energy filter (20 eV slit)	Gatan	
Selectris X energy filter + Falcon 4i detector	Thermo Fisher Scientific	
EM GP2 plunge freezer	Leica	
SP8 confocal laser scanning microscope (40×1.10 NA water objective)	Leica	
EZ-Grip denudation pipettor	CooperSurgical	Cat #7-72-2802

### Mouse embryo collection

Zygotes were collected from the oviducts of ~8–12-week-old timed-mated CD-1 mice (Charles River), and granulosa cells were removed by brief (~5 s) treatment with hyaluronidase (Sigma-Aldrich; #H4272, at 1 mg/ml, stored at −20 °C). Embryos were then washed and cultured at 37 °C under 5% CO₂. All procedures were performed in EmbryoMAX® Advanced KSOM medium (Millipore; #MR-101-D). All mice were maintained at 21–23 °C, 45–65% humidity, under a 12 h light/dark cycle (6:30–18:30).

### Mouse embryo blastomere separation and reassociation

Zona pellucida of 6/8-cell mouse embryos was removed by brief (~ 10 s) exposure to acidic Tyrode’s solution (Sigma-Aldrich; T1788), followed by five washes (~ 3–5 s each) in EmbryoMAX® Advanced KSOM medium (Millipore; #MR-101-D). Embryos were subsequently incubated in 0.5% trypsin, 1 mM EDTA solution for up to 2 min at 37 °C (prepared fresh in PBS from 2.5% trypsin, Gibco 15090046, and 0.5 M EDTA pH 8.0, Invitrogen 15575-038), followed by five washes (~3–5 s each) in 4% BSA, 0.5 mM EDTA solution to inactivate trypsin (prepared fresh in PBS from 30% BSA, Sigma A7284, and 0.5 M EDTA pH 8.0, Invitrogen 15575-038). Mechanical dissociation of blastomeres was performed in the 4% BSA, 0.5 mM EDTA solution using a transfer pipette with a tip of 50 µm diameter attached to an EZ-Grip denudation pipettor (~10 s). Disaggregated blastomeres were then either used for grid preparation or for reassociation experiments.

For reassociation experiments, they were washed through five times (3–5 s each) and finally cultured in 5 µl droplets of pre-equilibrated EmbryoMAX® Advanced KSOM medium at 37 °C under 5% CO₂. Small indentations were created in 5 µl culture droplets using a custom-fabricated stainless-steel needle to facilitate blastomere reassociation.

### Immunofluorescence

Mouse embryos were fixed in 4% methanol-free formaldehyde (Thermo Scientific; #28906) in phosphate-buffered saline (PBS) for 20 min at room temperature. Fixed embryos were permeabilized in PBS containing 0.2% Triton X-100 for 10 min at room temperature, then blocked in 1% BSA (Fisher BioReagents; BP1605-100) in PBS overnight at 4 °C. Primary antibodies were incubated overnight at 4 °C. The following primary antibodies were used: anti-SOX2 (Invitrogen; #14-9811-82, 1:200) and anti-GATA3 (Abcam; ab199428, 1:200). Secondary antibodies and DAPI (Vector Laboratories; #H-1200) were applied for 1 h at room temperature. Secondary antibodies were Donkey anti-Rabbit IgG (H + L), Alexa Fluor 488 (Invitrogen; #A-21206), and Donkey anti-Rat IgG (H + L), Alexa Fluor Plus 555 (Invitrogen; #A48270), both used at 1:400 dilution in 1% BSA in PBS.

### Confocal imaging

Confocal imaging was performed in PBS with 3 mg/ml polyvinylpyrrolidone (Sigma-Aldrich; #P0930) under paraffin oil in a 35 mm dish with a #1.5 glass coverslip. Images were acquired with an SP8 confocal laser scanning microscope (Leica) using a plan apochromatic 40 × 1.10 NA water-immersion objective.

### Grid preparation

The 6/8-cell mouse embryos were sequentially incubated in EmbryoMAX® Advanced KSOM medium supplemented with increasing concentrations of DMSO and ethylene glycol to aid vitrification (Jentoft et al, 2023): 1.75% each for 3 min, 2.33% each for 3 min, 3.5% each for 6 min, and 7.5% each for 3 min. Separated blastomeres from 6/8-cell mouse embryos were incubated in a similar manner but only until the second step which contained 2.33% of each solution. The cryoprotectant concentration used for separated blastomeres was determined empirically. Although blastomeres are smaller than intact embryos, they remain relatively large cells (~35 µm in diameter) and required cryoprotectants for reliable vitrification, particularly where two cells were positioned close to one another on the grid. We tested 0%, 1.75%, 2.33%, and 7.5% DMSO and ethylene glycol each. Vitrification was incomplete in the absence of cryoprotectants and at 1.75% of each, whereas blastomeres were reliably vitrified at 2.33% of each. We therefore adopted 2.33% DMSO and 2.33% ethylene glycol, an approximately threefold reduction relative to the concentration required for intact embryos.

From the start of blastomere dissociation to plunge-freezing, separated blastomeres were processed within ~9–10 min. Intact embryos or separated blastomeres were then applied onto freshly glow-discharged Quantifoil R2/2 200-square-mesh gold grids (Quantifoil) along with ~2–3 μl of the final media solution. The grid was then placed in an EM GP2 plunge freezer (Leica) at 95% humidity and 25 °C, and blotted for 6 s before being plunged into liquid ethane.

### Cryo-FIB lamella preparation

Grids were clipped into Autogrids (Thermo Fisher Scientific) and subjected to automated lamella preparation using an Aquilos 2 FIB-SEM (Thermo Fisher Scientific). The grids were first sputter-coated with an inorganic platinum layer for 30 s, followed by a coating of organometallic platinum using a gas injection system (GIS) for 40 s and an additional sputter-coat of an inorganic platinum layer for 30 s. Next, grids were surveyed using Maps software (Thermo Fisher Scientific) for lamella site identification. For separated blastomeres, this was followed by automated lamella preparation using AutoTEM Cryo (Thermo Fisher Scientific). Lamella were milled at an angle of 12° with a 30 kV ion beam in 4 steps: (1) 1 nA, gap 3 μm with stress-relief cuts, (2) 0.5 nA, gap 2 μm, (3) 0.3 nA, gap 1.4 μm, (4) 0.1 nA, gap 0.7 μm. Lamellae were finally polished at 30-50 pA with a gap set to 100 nm. For intact embryos, lamellae were prepared manually. Material in front and back of the area of interest of ~30–40 μm were milled away at an angle of 90°. Lamella were then milled using a similar stepwise milling protocol as above, along with stress-relief notches or cuts to minimize lamella deformation.

### Cryo-ET data acquisition

For intact embryo samples, tilt-series data were collected using a Titan Krios G3i transmission electron microscope (Thermo Fisher Scientific) operating at 300 kV equipped with a K3 detector and an energy filter with a 20 eV slit size (Gatan) and 100 μm objective aperture. Dose-symmetric tilt series in 3° steps were acquired using Tomography 5 software (Thermo Fisher Scientific) with a tilt span of ±48° around the milling angle. The target focus for each tilt-series was set in the range of −2 µm to −4.5 µm. Movies for each tilt were acquired at a magnification of ×53,000 in tiff format with 6 frames at a pixel size of 1.63 Å and a nominal dose of 3.5 e-/Å^2^.

For separated blastomere samples, tilt-series data were collected using a Titan Krios G4 transmission electron microscope operating at 300 kV equipped with a Selectris X energy filter with a slit set to 10 eV, 100 μm objective aperture, and a Falcon 4i direct electron detector (Thermo Fisher Scientific). Dose-symmetric tilt series in 3° steps were acquired using Tomography 5 software (Thermo Fisher Scientific) with a tilt span of ±48° around the milling angle. The target focus for each tilt-series was set in the range of −2 µm to −4.5 µm. Movies for each tilt were acquired at a magnification of ×64,000 (180 movies at 1.91 Å/pix) for the 14 embryo dataset or ×105,000 (1153 movies at 1.185 Å/pix) for the larger 66 embryo dataset in tiff format with 6–8 frames at a nominal dose of 3.5 e-/Å^2^.

### Cryo-ET data processing and subtomogram averaging

All data processing was performed in RELION-5 (Burt et al, 2024) unless specified otherwise and is described in Fig. EV3. The processing for both the intact embryo samples and separated blastomeres was performed in a similar manner, and here we explain the processing steps in the context of the latter sample dataset collected at the pixel size of 1.185 Å/pix. Global motion correction and dose-weighting were performed in RELION using MotionCor2 (Zheng et al, 2017) with a B-factor of 150 and 5 × 5 patches. The power spectrum of aligned frames was used for contrast transfer function estimation by CTFFIND4.1 (Rohou and Grigorieff, 2015). Tilt series alignment was then performed using Aretomo2 (Zheng et al, 2022) followed by tomogram generation in RELION at a pixel size of 10 Å. CPL filaments were first picked using PyTOM (Chaillet et al, 2025) using EMDB-16458 (Jentoft et al, 2023) filtered to 40 Å as a reference to get an initial model, and then this initial model was used for particle picking using PyTOM. Particles were extracted with a box size of 128 pixels at a pixel size of 4.74 Å followed by 3D refinement with the initial model filtered to 60 Å as a reference. 3D classification without alignment was performed, and 134,387 particles that belonged to classes displaying clear features were selected, followed by another round of 3D refinement. This was followed by CTF refinement and Bayesian polishing at a pixel size of 2.37 Å and a box size of 256 pixels, resulting in a structure at 8.7 Å resolution. This was followed by two rounds of particle extraction with a box size of 256 pixels at a pixel size of 2.37 Å followed by 3D refinement (with C2 symmetry), CTF refinement, and Bayesian polishing. The particles were then re-extracted at a pixel size of 1.185 Å and box size of 420 pixels, followed by 3D refinement with C2 symmetry to obtain a 5.8 Å structure. Given the pseudo C2 symmetry of the CPL repeat unit, particles were symmetry-expanded in RELION. A subsequent 3D refinement in C1 symmetry, using a mask encompassing the asymmetric unit, improved the resolution to 4.7 Å. Focused 3D classifications (without alignment) and refinements were then performed on distinct structural regions.

To resolve the inter-repeat bridging proteins, symmetry-expanded particles were recentered on the ZBED3 tetrahelical bundle and extracted with a 180-pixel box at 2.37 Å/pixel. These were subjected to focused 3D classification and refinement using a mask that included the bridging proteins and the NLRP5–TLE6–OOEP dimers on opposing CPL repeats.

To examine the inter-filament interaction between lateral FBXW–SKP1 and NLRP14-UHRF1, symmetry-expanded particles were recentered on lateral FBXW–SKP1 and extracted with a 180-pixel box at 2.37 Å/pixel, followed by focused 3D classification and refinement.

To resolve the states with density consistent with ubiquitin-charged UBE2D and central FBXW–SKP1 complex, 3D classification was performed using a mask around the back-face UHRF1-UBE2D-NLRP14-Tubulin complex, and duplicate particles were removed from each resulting class. This separated the particles into two states that differ in the arrangement of the back-face complex and in the contents of the central cavity. In one state (50,845 particles; 37.8%), the central cavity contained density consistent with ubiquitin-charged UBE2D. In the other (59,672 particles; 44.4%), the central cavity contained a central FBXW–SKP1 complex, and clear ubiquitin density was not observed. The remaining 17.8% of particles were poorly resolved and were not classified into either state. For each state, the best-resolving particles were then selected through a further round of 3D classification without a mask and subjected to 3D refinement with C1 symmetry using a mask encompassing the full CPL repeat unit. This yielded a 7.7 Å structure of the ubiquitin-bound state (30,390 particles) and a 7.3 Å structure of the central FBXW–SKP1 state (34,451 particles).

For visualizing the landscape of inter-filament contacts, the particles from the dataset collected on blastomeres from 14 embryos were used, as it was at a larger pixel size, which helped with 3D classification, as it included more surrounding filaments than would be possible with the lower pixel size dataset. All particles contributing to the consensus structure were used and were re-extracted with a box size of 200 pixels at a pixel size of 7.64 Å followed by 3D classification without alignment and no mask.

A composite map for a CPL repeat unit was generated by merging maps from all the local refinements in UCSF ChimeraX (Meng et al, 2023) using the “Volume max” function.

### Model building and refinement

To identify the proteins within the subtomogram averaging density maps of CPLs, we used a candidate-based search that first focused on proteins whose genetic deletion has been reported to result in loss of CPLs. These included PADI6, NLRP5, TLE6, OOEP, KHDC3, ZBED3, NLRP4F, UHRF1 and NLRP14. For each candidate, experimentally determined structures were used where available; otherwise, AlphaFold3 predictions were used as starting models. Candidate models were searched within the subtomogram averaging maps in UCSF ChimeraX using the “fit in map” function, with the “search” parameter set to 2000. Searches were performed iteratively. Once a density was assigned to a candidate, that region of the map was excluded from subsequent searches, thereby reducing the search space for remaining components. The proteins were identified in the following order.

For PADI6, we generated an AlphaFold3 model of dimeric mouse PADI6 (Appendix Fig. S2A), as human PADI6 is known to form dimers (Ranaivoson et al, 2024; Williams et al, 2024). This model closely matched the available crystal structure of human PADI6 (PDB-9FMN; RMSD = 2.2 Å) and was readily identified in the map based on the characteristic shape of the PADI6 dimer. This assignment is also supported by co-localization of PADI6 with CPLs using immunofluorescence super-resolution microscopy (Jentoft et al, 2023) and immunogold EM (Wright et al, 2003), and by the absence of CPLs in PADI6 knockout oocytes (Esposito et al, 2007; Yurttas et al, 2008).

For the NLRP5–TLE6–OOEP complex, we used the previously determined structure of the mouse complex (PDB-8H93) as an initial model. The complex fits the density as a dimer of NLRP5–TLE6–OOEP assemblies, consistent with previous structural and biochemical observations that this complex can dimerize (Chi et al, 2024). This assignment is also supported by co-localization of these proteins with CPLs using immunofluorescence super-resolution microscopy (Jentoft et al, 2023) and immunogold EM (Tashiro et al, 2010). An AlphaFold3 prediction was used to model the helical region of OOEP (residues 115–144; Appendix Fig. S2B), which was absent from the initial model used.

For KHDC3, we used an AlphaFold3 prediction as the starting model (Appendix Fig. S2C). Its N-terminal residues 1–136 were identified adjacent to OOEP in one of the NLRP5–TLE6–OOEP complexes within the dimeric assembly. The remainder of KHDC3 was not visualized and is likely flexible or disordered. This assignment is consistent with previous binding data showing that the KHDC3 N-terminus interacts with the NLRP5–TLE6–OOEP complex (Chi et al, 2024) and with the loss of CPLs upon KHDC3 knockout (Li et al, 2008; Ohsugi et al, 2008).

For ZBED3, an AlphaFold3 prediction of the BED-type zinc-finger domain (Appendix Fig. S2D) fit density adjacent to the TLE6 WD40 domain and OOEP KH domain, consistent with a structure of an NLRP5–TLE6–OOEP–ZBED3 complex published (Ou et al, 2025) during preparation of this work. A second zinc-finger-like density was observed on the opposite side of the TLE6 WD40 domain, although this region was resolved at lower resolution. Adjacent to these zinc-finger densities, we observed a coiled-coil density forming a four-helix bundle, the length of which matched the predicted C-terminal helical region of ZBED3. An AlphaFold3 prediction of four ZBED3 molecules generated a high-confidence four-helix bundle formed by the C-terminal helices (Appendix Fig. S2E) that fit this density well and was compatible with the positions of the N-terminal zinc-finger domains. This assignment is further supported by evidence that ZBED3 interacts with NLRP5–TLE6–OOEP and that its knockout in mouse oocytes leads to loss of CPLs (Gao et al, 2018; Qin et al, 2019).

For NLRP4F, an AlphaFold3 prediction was used as the starting model (Appendix Fig. S2F). The model matched the density linking NLRP5–TLE6–OOEP-ZBED3/KHDC3 complexes of adjacent repeat units. Among NLRP proteins expressed in oocytes and early embryos (Harasimov et al, 2024; Zhu et al, 2025), NLRP4F provided the best match to the observed density, particularly in the length of its LRR region. Other candidate NLRP proteins were incompatible with the density: NLRP4A, NLRP4B, and NLRP9B have longer LRR regions than observed, whereas NLRP2 has a substantially shorter LRR region. This assignment is further supported by previous evidence that NLRP4F interacts with the NLRP5–TLE6–OOEP complex (Gao et al, 2018; Qin et al, 2019), and by proteomic data (Jentoft et al, 2023) showing that NLRP4F, KHDC3, and ZBED3, together with NLRP5 and OOEP, are significantly reduced in TLE6 knockout oocytes.

For UHRF1 and NLRP14, AlphaFold3 predictions were used as starting models (Appendix Fig. S3A,B). UHRF1 contains five domains: UBL, TTD, PHD, SRA, and RING. Each domain was fitted independently into the density. The SRA and RING domains were identified using the ‘fit in map‘ function in ChimeraX, whereas the remaining domains were placed using a combination of manual fitting and local ‘fit in map’ searches. The resulting domain positions were consistent with their order and connectivity in the primary sequence. NLRP14 fit the density associated with UHRF1, and the length of its LRR region matched the observed density. This assignment is consistent with data showing that NLRP14 and UHRF1 interact (Zhang et al, 2023) and colocalize with PADI6 (Jentoft et al, 2023). Furthermore, both proteins are required for CPL assembly (Uemura et al, 2023; Zhang et al, 2023).

Together, these assignments accounted for all nine candidate factors whose loss has been reported to result in the disappearance of CPLs. In each case, the fitted model was consistent with the subtomogram averaging density and with previously mapped interactions among CPL and SCMC components. After assigning these nine factors, the remaining densities were searched against additional proteins associated with CPLs or known to interact with the assigned components, focusing on factors whose abundance is reduced upon CPL loss in PADI6- or TLE6-deficient oocytes (Jentoft et al, 2023).

For UBE2D, an AlphaFold3 prediction of mouse UBE2D3 was used as the starting model (Appendix Fig. S3C). The density between the UHRF1 SRA and RING domains had the characteristic fold of an E2 ubiquitin-conjugating enzyme and fit the UBE2D3 model well. This assignment was supported by the known interaction of UHRF1 specifically with UBE2D-family E2 enzymes (DaRosa et al, 2018; Foster et al, 2018). Among E2 enzymes detected in oocyte proteomic data, UBE2D3 is the only one significantly reduced in PADI6 knockout oocytes (Jentoft et al, 2023), where UHRF1 is also among the most strongly reduced proteins. UBE2D3 is also the most abundant E2 enzyme in mouse oocytes and embryos (Harasimov et al, 2024; Zhu et al, 2025), consistent with other CPL constituents being highly abundant during early development. The map resolution was not sufficient to distinguish confidently between closely related UBE2D isoforms, which share an almost identical fold. We therefore modeled UBE2D3 in the density but annotated the assignment as UBE2D to reflect this ambiguity.

For ubiquitin, the ubiquitin moiety from PDB-6TTU was used as the starting model. Although the resolution was insufficient to resolve individual β-strands, the size and overall shape of the density were consistent with ubiquitin, and its position immediately adjacent to UBE2D supported this assignment. The density was located next to the UBE2D catalytic cysteine, at a distance consistent with a covalently linked E2~ubiquitin conjugate (Baek et al, 2020; Branigan et al, 2015; Dou et al, 2012), and its position and orientation relative to UBE2D resembled the open E2~ubiquitin conformation observed in previously determined structures (Juang et al, 2012; Wiener et al, 2013). The density was present only in a subset of particles consistent with a substoichiometric ubiquitin-charged state rather than a constitutive component of the CPL repeat.

For αβ-tubulin, the density was assigned based on the characteristic αβ-tubulin fold. The AlphaFold3 model of TUBA1C-TUBB4B dimer was used as the starting model (Appendix Fig. S3D), as these are among the abundant α- and β-tubulin isoforms in mouse oocyte and embryo transcriptomic and proteomic datasets (Boroviak et al, 2018; Harasimov et al, 2024; Zhu et al, 2025). However, because tubulin isoforms are highly similar, the identities of the α- and β-tubulin isoforms cannot be unambiguously distinguished at the current resolution. The density was nevertheless sufficient to distinguish α- from β-tubulin based on features including the S9–S10 loop (Fig. EV4B).

Finally, density containing an eight-bladed WD40 β-propeller with a β-hairpin insert was assigned to an FBXW–SKP1 complex, and an AlphaFold3 prediction of an FBXW15-SKP1 complex was used as a starting model for model building (Appendix Fig. S3E). A survey of WD40-domain-containing proteins in the proteomic and transcriptomic data showed that mouse orthologues of human FBXW12 contain an eight-bladed WD40 β-propeller with a β-hairpin insert matching the density. The adjacent unassigned density matched SKP1, the canonical partner of FBXW proteins. Because many closely related mouse orthologues of human FBXW12 exist (FBXW13-28), the exact identity could not be determined at our resolution; we tentatively built FBXW15, the mouse orthologue annotated as most abundant in proteomic data from early embryos and oocytes (Harasimov et al, 2024; Zhu et al, 2025). Furthermore, SKP1 is known to colocalize with PADI6 in mouse oocytes using immunofluorescence super-resolution microscopy (Jentoft et al, 2023).

After assignment of individual proteins and complexes, the model was refined through multiple cycles of restrained flexible fitting in ISOLDE (Croll, 2018), with distance restraints of 3–8 Å imposed on starting models, followed by user-guided refinement in COOT (Emsley et al, 2010). Regions that were not supported by density were omitted from the final model, and side chains were truncated where they could not be confidently modeled. Final model refinement and validation were performed in PHENIX (Afonine et al, 2018). The starting models used for each protein, the residues built, and copy numbers are summarized in Appendix Table S2. Refinement statistics are reported in Appendix Table S1.

### Data visualization

Tomograms were denoised using cryoCARE (Buchholz et al, 2019). Membranes were segmented using MemBrain v2 (PREPRINT: Lamm et al, 2025). Ribosomes and microtubules were picked using PyTOM for tomogram segmentation. ArtiaX plugin (Ermel et al, 2022) within UCSF ChimeraX was used for mapping back structures to their coordinates in the original tomogram, as displayed in Fig. 1B. All structural figures were rendered in UCSF ChimeraX.

## Supplementary information

Peer Review File

Appendix

Movie EV1

Movie EV2

Source data Fig. 1

Appendix Figure Source Data

Expanded View Figures

## Data Availability

Atomic coordinates and cryo-EM maps have been deposited in the Protein Data Bank or Electron Microscopy Data Bank, respectively, under accession codes PDB 9T63 and EMD-55603 (Asymmetric unit of a CPL repeat), PDB 9T64 and EMD-55604 (CPL repeat unit with the central FBXW–SKP1 complex), PDB 9T67 and EMD-55607 (CPL repeat unit with ubiquitin bound), PDB 9T66 and EMD-55606 (Complex linking two CPL filament repeat units), PDB 9T65 and EMD-55605 (Complex linking two CPL filaments), PDB 9T68 and EMD-55609 (Composite structure of a CPL repeat unit) and EMD-55608 (Subtomogram averaging map of CPL repeat unit structure from intact mouse embryos). The source data of this paper are collected in the following database record: biostudies:S-SCDT-10_1038-S44318-026-00895-w.
