# Supplementary material for: In-situ cryo-ET of mouse embryos reveals cytoplasmic lattices contain ubiquitin-charged E2-E3 ligase assemblies
Source: EMBO J. 2026 Aug 10;45(21):7405–37. doi: 10.1038/s44318-026-00895-w (PMC13728194; doi:10.1038/s44318-026-00895-w)
Supplement: Supplementary file 1 [file 44318_2026_895_MOESM1_ESM.pdf]

THE EMBO JOURNAL

# In-situ cryo-ET of mouse embryos reveals cytoplasmic lattices contain ubiquitin-charged E2-E3 ligase assemblies

Kashish Singh, Katarina Harasimov, Kathy Niakan, and Andrew Carter

*Corresponding authors: Kashish Singh (ksingh@mrc-lmb.cam.ac.uk) , Andrew Carter (cartera@mrc-lmb.cam.ac.uk)*

**Review Timeline:**

| | |
|---|---|
| Submission Date: | 23rd Mar 26 |
| Editorial Decision: | 20th Apr 26 |
| Revision Received: | 17th Jul 26 |
| Accepted: | 24th Jul 26 |

*Editor: Ieva Gailite*

## Transaction Report:

(Note: With the exception of the correction of typographical or spelling errors that could be a source of ambiguity, letters and reports are not edited. Depending on transfer agreements, referee reports obtained elsewhere may or may not be included in this compilation. Referee reports are anonymous unless the Referee chooses to sign their reports.)

Dear Dr. Singh,

Thank you for submitting your manuscript for consideration by the EMBO Journal. We have now received comments from two reviewers, which are included below for your information.

As you will see, both reviewers appreciate the contribution of the study to the research field and recommend publication of the article after textual revisions as outlined in their comments.

Based on these generally positive assessments and the interest expressed in the reports, I would like to invite you to submit a revised manuscript in response to the reviewers' comments. Please also see the attached instructions for formatting guidelines on preparation of the revised manuscript.

We generally allow three months as standard revision time, which can be extended to six months in the case of major revisions. Should you foresee a problem in meeting this deadline, please let us know in advance to discuss an extension.

As a matter of policy, competing manuscripts published during this period will not negatively impact on our assessment of the conceptual advance presented by your study. However, please contact me as soon as possible upon publication of any related work to discuss the appropriate course of action.

When preparing your letter of response to the referees' comments, please bear in mind that this will form part of the Review Process File and will therefore be available online to the community. For more details on our Transparent Editorial Process, please visit our website: https://www.embopress.org/page/journal/14602075/authorguide#transparentprocess.

Please feel free to contact me if you have any further questions regarding the revision. Thank you for the opportunity to consider your work for publication. I look forward to receiving the revised version!

With best wishes,

Ieva

---

Ieva Gailite, PhD
Senior Scientific Editor
The EMBO Journal
Meyerhofstrasse 1
D-69117 Heidelberg
Tel: +4962218891309
i.gailite@embojournal.org

Instructions for preparing your revised manuscript:

Read our guidance for manuscript revisions and related editorial policies: https://link.springer.com/journal/44318/submission-guidelines#cms-Revised-submissions

Please also check that the title and abstract of the manuscript are brief, yet explicit, even to non-specialists.

When assembling figures, please refer to our figure preparation guideline in order to ensure proper formatting and readability in print as well as on screen:
https://media.springernature.com/original/springer-cms/rest/v1/content/27825798/data/v1

IMPORTANT: When you send the revision we will require
- a point-by-point response to the referees' comments, with a detailed description of the changes made (as a word file).
- a word file of the manuscript text.
- individual production quality figure files (one file per figure)
- a complete author checklist
- Expanded View files (replacing Supplementary Information)
- a Reagents and Tools Table as part of the Methods section

Please remember: Digital image enhancement is acceptable practice, as long as it accurately represents the original data and conforms to community standards. If a figure has been subjected to significant electronic manipulation, this must be noted in the

figure legend or in the 'Methods' section. The editors reserve the right to request original versions of figures and the original images that were used to assemble the figure.

We realize that it is difficult to revise to a specific deadline. In the interest of protecting the conceptual advance provided by the work, we recommend a revision within 3 months (19th Jul 2026). Please discuss the revision progress ahead of this time with the editor if you require more time to complete the revisions.

------------------------------------------------

Referee #1:

Singh et al. resolve the in situ structure of cytoplasmic lattices (CPLs) in early mouse embryos. Rather than thinning intact embryos at the 6-8-cell stage, the authors isolate individual blastomeres, thereby reducing (while unfortunately not completely avoiding) the need for cryoprotectants and enabling automated FIB-milling for scalable lamella preparation. Using this approach, they determine the structure of the large multiprotein CPL assemblies. They structurally identifyt the position and stoichiometry of 14 distinct proteins, which they assign to an essential CPL scaffold, linker components connecting repeating units, and a CPL core complex. The latter accommodates ubiquitin-charged UBE2D molecules in potentially both active and inactive conformations, suggesting a mechanism for regulated ubiquitin transfer. The work is thorough and innovative, and the manuscript describes it very clearly, providing a detailed structural description of the CPLs architecture with implications for their functional states. The topic is clearly of outstanding scientific interest. I fully support publication in EMBO Journal as soon as possible. The authors are encouraged to address the comments below that are mainly aimed at better positioning this work, with its highlight of being performed in situ, in light of recent publications. In particular, the author's work on early-stage embryo samples (vs unfertilized oocytes) open an opportunity for thorough comparison with these studies, in particular with relation to potential changes in ubiquitination requirements at different developmental stage.

Major concerns:

1. There is substantial overlap with recently published studies (Nature, s41586-026-10442-6 and s41586-026-10360-7), in which CPL assemblies were purified from oocytes and their architectures were resolved by cryo-EM. The first study identified the same 14 proteins as CPL components. In addition, it resolved three FBXW homologs (FBXW18/19/21), which could not be distinguished in the present work due to resolution limitations. Notably, the reported stoichiometry and details of the architectural arrangement differ between the two studies. This discrepancy should be explicitly addressed and discussed.
2. The authors do not sufficiently mention recently published related work (PMIDs: 41845018, 41772195). A discussion of how these studies corroborate or differ from the present findings would significantly strengthen the manuscript. In particular, differences in organism (human vs. mouse) and developmental stage (unfertilized oocytes vs. early embryos) may account for structural and functional divergences and should be considered.
3. The authors describe two distinct structural states of the CPL core complex: one in which ubiquitin is bound and the E2~ubiquitin conjugate adopts an open, catalytically less active conformation, and a second state lacking resolved ubiquitin but featuring an additional central FBXW-SKP1 complex, accompanied by rearrangements suggestive of a catalytically competent closed conformation. To my knowledge, this latter state has not been reported previously. The authors should expand their discussion of this finding, particularly in the context of developmental stage-specific requirements (e.g., potential need for ubiquitination in early embryos versus oocytes). This proposed active state would benefit from validation by orthogonal approaches, but it is also understandable if this is beyond the scope of the current work. I recommend that the authors explicitly acknowledge the need for further validation of their functional hypotheses presented in the discussion.

Minor concerns:

- Introduction starts with description of human embryonic development, which seems somewhat odd given the work's focuses on mouse embryos. The authors should consider rephrasing to mammalian embryonic development instead.
- Description of the construction of the structural model could be made clearer, beyond the current "...adopted a candidate-based approach focussing 132 on proteins implicated with CPLs". The authors nicely provide a supplementary table, but it would be useful to include some description in the main text on where they used structure prediction, fitting into the maps, or use of existing structures of complexes. Alternatively, the authors are encouraged to expand this section in the methods. It is exceptionally minimal in the current version.
- Confidence scores and different metrics of the structure predictions and fits to the maps are completely missing.
- The relative prevalence of the ubiquitin-bound versus FBXW-SKP1-associated states is unclear and should be quantified or discussed.
- Figure 1: please include scale bars on all light and electron microscopy images.
- Figure 2B: ZBED3 appears to be missing from the CPL component list. It would also be useful to indicate the colors with which the proteins are depicted in the maps in A.
- It would be useful to add to the methods the duration of the mouse embryo blastomere separation procedure, and time from

isolation to plunge freezing.
- All sample vitrified samples were in the presence of DMSO and ethylene glycol. Was this really necessary for the isolated blastomeres? Is there data validating that the CPLs are similar at light microscopy level (for example with immunofluorescence) with and without the addition of DMSO and ethylene glycol.

Referee #2:

Singh et al. describe the in situ analysis of cytoplasmic lattices by cryo-electron tomography. Cytoplasmic lattices (CPL) are a protein storage assembly unit crucial for embryonal development. The precise molecular structure of CPLs was previously unknown. Using a new method to dissociate mouse embryos into individual blastomeres the authors could determine the 5.8 Å structure of a cytoplasmic lattice. The authors can conclusively identify the protein components of CPLs and establish the molecular architecture of how individual components assemble into a ~4.5 MDa asymmetric unit and further oligomerization and filament formation. Intriguingly, the in situ structure shows the presence of various ubiquitylation components such as a ubiquitin-loaded UBE2D complex. As whole this work clearly establishes the molecular architecture of cytoplasmic lattices in situ and highlights how CPLs are tightly associated with the ubiquitylation machinery.

There are a handful of comments/issues that the authors need to address:

1. The works of several other groups (Zhang et al.; Li et al.; Liu et al.) also report the structure of CPLs obtained through single particle work-flows. The authors should ensure proper citation of these publication and preprints and include a comparison to these structures in their discussion where differences arise. Such discrepancies are the number of UHRF1/NLRP14 subunits per ASU or the visibility of additional ZBED3 densities connecting ASUs.

2. The authors currently do not assign the FBXW subunits to a specific member of the FBXW family. The authors should reference current works on the identification of CPL components (such as Williams et al. bioRxiv 2026) to indicate the possible FBXW proteins. As the FBXW subunits bind to different parts of the CPL assembly the authors should discuss whether these FBXWs are likely the same protein or different proteins of the same family. Additionally, the authors should discuss whether FBXW-SKP1 complexes are competent to assemble with their canonical binding partner CUL1.

3. The authors should add a short discussion on the tubulin components part of the CPL. What is the nucleotide state? How do these tubulins differ between assembled or free tubulins?

4. The author's observation of two related CPL states containing a ubiquitin-charged UBE2D complex or a rearranged CPL with no visible UB is an exciting observation. Considering the labile nature of E2~UB complexes it is incredible to visualize such a transient intermediate in situ. We kindly suggest the authors to cite the works of Juang et al. 2012 (PMID: 22325355) and Wiener et al. 2013 (PMID: 23955022) which observed an almost identical E2-UB conformation. The author's claim that the rearrangements indicate a form of regulated ubiquitin transfer are not supported by their data. Such an activation mechanism cannot be drawn from purely structural data. It is also wholly unclear how the binding of an additional FBXW-SKP1 complex relates to the rearrangement of the CPL to seemingly "activate" the UBE2D-UB complex. In the absence of any functional data that would indicate ubiquitin transfer occurring as part of a CPL the authors should rephrase their conclusions.

Minor:
1. Instead of writing that UHRF1 is bound to "its known E2 ubiquitin-conjugating enzyme UBE2D". The authors should indicate that UHRF1 is bound to a member of the UBE2D-family as the resolution will now allow conclusive differentiation between members of the family.

2. It would be of interest to the reader to include a short discussion of how the current structural work might inform the mechanisms of CPL disassembly.

3. In Figure 5a, the domain diagram for FBXW should be generalized by removing the residue numbers. As the authors cannot assign their FBXW densities to a specific FBXW protein the domain diagram should reflect this ambiguity.

Referee #1:

Singh et al. resolve the in situ structure of cytoplasmic lattices (CPLs) in early mouse embryos. Rather than thinning intact embryos at the 6-8-cell stage, the authors isolate individual blastomeres, thereby reducing (while unfortunately not completely avoiding) the need for cryoprotectants and enabling automated FIB-milling for scalable lamella preparation. Using this approach, they determine the structure of the large multiprotein CPL assemblies. They structurally identifyt the position and stoichiometry of 14 distinct proteins, which they assign to an essential CPL scaffold, linker components connecting repeating units, and a CPL core complex. The latter accommodates ubiquitin-charged UBE2D molecules in potentially both active and inactive conformations, suggesting a mechanism for regulated ubiquitin transfer. The work is thorough and innovative, and the manuscript describes it very clearly, providing a detailed structural description of the CPLs architecture with implications for their functional states. The topic is clearly of outstanding scientific interest. I fully support publication in EMBO Journal as soon as possible. The authors are encouraged to address the comments below that are mainly aimed at better positioning this work, with its highlight of being performed in situ, in light of recent publications. In particular, the author's work on early-stage embryo samples (vs unfertilized oocytes) open an opportunity for thorough comparison with these studies, in particular with relation to potential changes in ubiquitination requirements at different developmental stage.

Major concerns:

1. There is substantial overlap with recently published studies (Nature, s41586-026-10442-6 and s41586-026-10360-7), in which CPL assemblies were purified from oocytes and their architectures were resolved by cryo-EM. The first study identified the same 14 proteins as CPL components. In addition, it resolved three FBXW homologs (FBXW18/19/21), which could not be distinguished in the present work due to resolution limitations. Notably, the reported stoichiometry and details of the architectural arrangement differ between the two studies. This discrepancy should be explicitly addressed and discussed.

We thank both referees for raising this point, which is also raised by Referee #2 (Major concern 1). These studies appeared during the revision of our manuscript, and we have added a dedicated section to the Discussion comparing our in situ structure with the recently reported single-particle cryo-EM structures of CPLs extracted from mouse oocytes (Chi et al, 2026; Kilic et al, 2026; Li et al, 2026; Liu et al, 2026; Zhang et al, 2026) (lines 350-394).

Briefly, these studies identify the same broad set of components. However, they differ from our in situ structure and, notably, from one another, despite all being purified from mouse oocytes. We now discuss these differences in composition, stoichiometry and architecture.

Two of the isolated CPL structures (Liu et al, 2026; Kilic et al, 2026) lack many subunits that we and other studies resolve (Chi et al, 2026; Li et al, 2026; Zhang et al, 2026), possibly reflecting partial disruption during CPL extraction. Two of the studies assume C2 symmetry (Li et al, 2026; Zhang et al, 2026), which increases the apparent copy number of certain components, such as the UHRF1-UBE2D-NLRP14-Tubulin subunits. Our asymmetric reconstructions instead show that the CPL repeat unit is pseudo-symmetric, in agreement with the CPL structure reported by Chi et al. (2026). Our

structure additionally resolves components that are not reported in any of the structures of CPLs from other studies, including four rather than two ZBED3 molecules per repeat and the proteins mediating inter-filament interactions. Additionally, we find that the attachment of a CPL filament to four neighbours, described in all of the other studies, corresponds to the unclassified population in our data, which represents a mixture of states prior to 3D classification. After classification, we instead find that CPL filaments make zero to three stable inter-filament contacts providing the structural basis for CPL lattice formation. Together, these observations account for the differences in composition and stoichiometry among the reported structures.

We also note that the single-particle studies provide complementary information on FBXW identity. Although our resolution did not allow us to discriminate between closely related FBXW family members, we now cite the assignment of the front-face, back-face and central-cavity complexes to FBXW18, FBXW19 and FBXW21, respectively, by Chi et al (2026) and Li et al (2026). The identity of the fourth FBXW-SKP1 complex, which we resolve at the inter-filament contacts, remains unclear; however, its distinct binding interface with PADI6 suggests that it could be a different isoform.

2. The authors do not sufficiently mention recently published related work (PMIDs: 41845018, 41772195). A discussion of how these studies corroborate or differ from the present findings would significantly strengthen the manuscript. In particular, differences in organism (human vs. mouse) and developmental stage (unfertilized oocytes vs. early embryos) may account for structural and functional divergences and should be considered.

As described in our response to Major concern 1, we have now added a dedicated section to the Discussion in which we cite and compare all of the recently reported CPL structures, including the studies noted by the referee (lines 350-394).

Regarding developmental stage, this is directly relevant to the most striking difference between our structure and the oocyte-derived structures (Chi et al, 2026; Kilic et al, 2026; Li et al, 2026; Liu et al, 2026; Zhang et al, 2026). Beyond resolving additional subunits and lattice-related features, we observe states that are consistent with UBE2D being charged with ubiquitin, which were not resolved in any of the structures from oocytes. We now discuss two possible explanations for this in the Discussion (lines 385-391). The ubiquitin-charged state may be labile and lost during CPL extraction. Alternatively, ubiquitin charging of CPL-associated UBE2D may be developmentally regulated and become more prominent after fertilisation. Distinguishing between these possibilities will require in situ structural analysis across defined developmental stages, comparing oocytes with early embryos, which our workflow is well placed to enable.

Regarding organism, all of the reported CPL structures, including ours, are derived from mouse. The only human structure currently available is of a reconstituted PADI6-UHRF1-UBE2D complex (PMID: 41772195). This complex lacks the other CPL components and adopts a different architecture: UHRF1 is bound to a single PADI6 dimer rather than to the helical PADI6 array, and the assembly lacks NLRP14 and the other factors that engage UHRF1 or are in close proximity within CPLs. It is therefore unlikely to represent the architecture or the activity of CPLs in human oocytes and embryos, and a direct cross-species comparison will require structural analysis of human CPLs.

We do, however, discuss the proposal, based on the reconstituted human complex and on the mouse oocyte CPL structures, that UHRF1 is inactive within CPLs. In the Discussion (lines 418-463), we explain why we think the current evidence does not establish whether UHRF1 is active or inactive in this context, and note that resolving this will require further biochemical investigation.

3. The authors describe two distinct structural states of the CPL core complex: one in which ubiquitin is bound and the E2~ubiquitin conjugate adopts an open, catalytically less active conformation, and a second state lacking resolved ubiquitin but featuring an additional central FBXW-SKP1 complex, accompanied by rearrangements suggestive of a catalytically competent closed conformation. To my knowledge, this latter state has not been reported previously. The authors should expand their discussion of this finding, particularly in the context of developmental stage-specific requirements (e.g., potential need for ubiquitination in early embryos versus oocytes). This proposed active state would benefit from validation by orthogonal approaches, but it is also understandable if this is beyond the scope of the current work. I recommend that the authors explicitly acknowledge the need for further validation of their functional hypotheses presented in the discussion.

We thank the referee for these suggestions, which we have implemented as follows:

First, we have expanded our discussion of the ubiquitin-bound state, which was not resolved in any of the structures of CPLs extracted from mouse oocytes (Chi *et al*, 2026; Kilic *et al*, 2026; Li *et al*, 2026b; Liu *et al*, 2026; Zhang *et al*, 2026). We discuss two possible explanations for this difference (lines 385-391). The ubiquitin-charged state may be labile and lost during CPL purification. Alternatively, and as the referee suggests, ubiquitin charging of CPL-associated UBE2D may be developmentally regulated, becoming more prominent after fertilisation, when early embryos undergo extensive changes in protein abundance and localisation that could increase the demand for ubiquitination.

Second, we have changed our text throughout the manuscript to avoid implying that our structure directly demonstrates ubiquitination within CPLs, and instead to convey that it shows CPLs possess the features required to accomplish ubiquitin transfer (lines 418-440). Further, we state explicitly that the proposal that CPLs act as sites of ubiquitination is a hypothesis raised by our structural data, which remains to be directly tested (lines 464-469). We agree with the referee that such validation is beyond the scope of the present work, and the Discussion now states that future studies will be required to determine whether ubiquitin transfer occurs within CPLs.

Minor concerns:

- Introduction starts with description of human embryonic development, which seems somewhat odd given the work's focuses on mouse embryos. The authors should consider rephrasing to mammalian embryonic development instead.

We have changed the introduction as suggested. Briefly, we have removed the part about human development being inefficient and error prone. Instead, now it is focussed on maternal factors being important for early mammalian development and critical nature of CPLs during this phase.

- Description of the construction of the structural model could be made clearer, beyond the current

"...adopted a candidate-based approach focussing 132 on proteins implicated with CPLs". The authors nicely provide a supplementary table, but it would be useful to include some description in the main text on where they used structure prediction, fitting into the maps, or use of existing structures of complexes. Alternatively, the authors are encouraged to expand this section in the methods. It is exceptionally minimal in the current version.

We have substantially expanded the description of how the molecular model was constructed. The Methods now include a detailed "Model building and protein identification" section (lines 613-730) describing our candidate-based approach. Where experimentally determined structures were available, these were used as starting models; otherwise we generated AlphaFold3 predictions. Each candidate model was then fitted into the subtomogram averaging maps using the UCSF ChimeraX "fit in map" function, and this was done iteratively, with every assigned density excluded from subsequent rounds to progressively reduce the space that remained to be searched. We also describe the order in which the individual components were identified. For each component, the section now details the specific evidence supporting its assignment, which was previously presented separately in Appendix Table S2. The table now summarizes the starting model used, the residues built and the copy number for each component within the CPL repeat unit.

- Confidence scores and different metrics of the structure predictions and fits to the maps are completely missing.

We agree that these metrics are important for assessing the reliability of our assignments, and we have now added them at three levels.

First, the AlphaFold3 predictions used as starting models throughout the manuscript are now presented in Appendix Figure S2 and S3, with each model coloured by pLDDT and accompanied by its predicted aligned error (PAE) plot. This allows the confidence of each prediction, including the interfaces within multi-chain predictions, to be assessed directly.

Second, the agreement between the refined models and the subtomogram averaging density is shown for each component in Figure EV4A, where the fitted atomic models are displayed within their corresponding densities.

Third, we have added the cross-correlation values of the built models against the maps to Appendix Table S1, alongside the final refinement and validation statistics.

Together, these additions provide quantitative confidence estimates for both the structure predictions and their fit to the experimental density.

- The relative prevalence of the ubiquitin-bound versus FBXW-SKP1-associated states is unclear and should be quantified or discussed.

The relative abundance of the two states is now indicated in the subtomogram averaging processing workflow (Figure EV3), explicitly stated in the Results (lines 301-303) and better described the image processing for classification in the Methods (lines 591-603).

To summarize, the two states occur at comparable frequencies. The ubiquitin-bound state accounts for 37.8% of CPL repeat units and the central FBXW-SKP1-associated state for 44.4%. The remaining 17.8% of particles were poorly resolved and were not classified into either state.

- Figure 1: please include scale bars on all light and electron microscopy images.

We have added scale bars to all light and electron microscopy images in Figure 1.

- Figure 2B: ZBED3 appears to be missing from the CPL component list. It would also be useful to indicate the colors with which the proteins are depicted in the maps in A.

Thank you for pointing this out. We have added ZBED3 to the CPL component list in Figure 2B. We have also updated the panel so that all components are colored according to the scheme used in Figure 2A.

- It would be useful to add to the methods the duration of the mouse embryo blastomere separation procedure, and time from isolation to plunge freezing.

We have added this information to the Methods. The duration of the blastomere separation procedure is now described in the "Mouse embryo blastomere separation and reassociation" section, and the total time from blastomere isolation to plunge-freezing is provided in the "Grid preparation" section (lines 523-524).

- All sample vitrified samples were in the presence of DMSO and ethylene glycol. Was this really necessary for the isolated blastomeres? Is there data validating that the CPLs are similar at light microscopy level (for example with immunofluorescence) with and without the addition of DMSO and ethylene glycol.

During optimization of the blastomere workflow, we tested a range of cryoprotectant concentrations: 0%, 1.75%, 2.33% and 7.5% DMSO and ethylene glycol (each). In the absence of cryoprotectants, and at 1.75% of each, vitrification was incomplete. Blastomeres were reliably vitrified at 2.33% DMSO and 2.33% ethylene glycol, and we therefore adopted this concentration to ensure efficient vitrification, avoid preparing lamellae from poorly vitrified cells, and maximize the proportion of samples suitable for data collection. This rationale has now been added to the "Grid preparation" section of the Methods (lines 514-522). This requirement is consistent with the relatively large size of 6/8-cell-stage blastomeres (~35 µm in diameter). Even cryo-ET studies of HeLa cells, which at ~15-25 µm are smaller than blastomeres, frequently use cryoprotectants such as 10% glycerol to improve vitrification (for example, PMID-36746945, PMID-41618010, PMID-40097852).

We have not performed a direct light-microscopy comparison of CPL organization in the presence and absence of DMSO and ethylene glycol. However, three independent lines of evidence indicate that the low cryoprotectant concentration used here does not detectably alter CPL structure.

First, the CPL structures obtained from separated blastomeres and from intact embryos are highly similar, despite the substantially higher cryoprotectant concentration required to vitrify intact embryos. A three-fold difference in cryoprotectant concentration therefore produces no detectable change in the CPL architecture resolved by cryo-ET.

Second, our in situ structure resolves the full set of components reported in single-particle cryo-EM studies of CPLs purified from mouse oocytes, together with additional lattice-associated components not resolved in those studies. This indicates that our vitrification conditions preserve CPL composition and higher-order organization.

Third, DMSO and ethylene glycol are widely used for the cryopreservation of mammalian oocytes and embryos. In mice, cryopreservation with 10% DMSO and 10% ethylene glycol, four-fold higher than the concentration used here, has been reported not to affect fertilization or birth rates (PMID: 40981676), indicating that these reagents are compatible with subsequent developmental progression under appropriate conditions.

Referee #2:

Singh et al. describe the in situ analysis of cytoplasmic lattices by cryo-electron tomography. Cytoplasmic lattices (CPL) are a protein storage assembly unit crucial for embryonal development. The precise molecular structure of CPLs was previously unknown. Using a new method to dissociate mouse embryos into individual blastomeres the authors could determine the 5.8 Å structure of a cytoplasmic lattice. The authors can conclusively identify the protein components of CPLs and establish the molecular architecture of how individual components assemble into a ~4.5 MDa asymmetric unit and further oligomerization and filament formation. Intriguingly, the in situ structure shows the presence of various ubiquitylation components such as a ubiquitin-loaded UBE2D complex. As whole this work clearly establishes the molecular architecture of cytoplasmic lattices in situ and highlights how CPLs are tightly associated with the ubiquitylation machinery.

There are a handful of comments/issues that the authors need to address:

1. The works of several other groups (Zhang et al.; Li et al.; Liu et al.) also report the structure of CPLs obtained through single particle work-flows. The authors should ensure proper citation of these publication and preprints and include a comparison to these structures in their discussion where differences arise. Such discrepancies are the number of UHRF1/NLRP14 subunits per ASU or the visibility of additional ZBED3 densities connecting ASUs.

This comment is similar to Major concern 1 raised by Reviewer 1 and is answered in detail there. Briefly, we have now cited all of the related publications and preprints (Chi et al., 2026; Kilic et al., 2026; Li et al., 2026; Liu et al., 2026; Zhang et al., 2026) and added a comparison to the Discussion (lines 350-394). This comparison addresses not only the differences between our in situ structure and those solved from CPLs extracted from mouse oocytes, but also the substantial differences that exist among the CPL structures extracted from oocytes themselves.

2. The authors currently do not assign the FBXW subunits to a specific member of the FBXW family. The authors should reference current works on the identification of CPL components (such as Williams et al. bioRxiv 2026) to indicate the possible FBXW proteins. As the FBXW subunits bind to different parts of the CPL assembly the authors should discuss whether these FBXWs are likely the same protein or different proteins of the same family. Additionally, the authors should discuss whether FBXW-SKP1 complexes are competent to assemble with their canonical binding partner CUL1.

The first part of this comment, regarding the identity of the FBXW subunits, is similar to Major concern 1 raised by Reviewer 1 and is answered in detail there. Briefly, we have now cited the recently reported CPL structures that define the identity of the FBXW proteins (Chi et al (2026) and Li et al (2026)) and discussed the identity of the FBXW proteins in the Discussion (lines 364-369).

The second part of the comment, regarding the ability of the FBXW-SKP1 complexes to assemble with CUL1, is now addressed in the Results (lines 252-260; Appendix Figure S1A). The binding modes of the FBXW-SKP1 complexes within CPLs appear incompatible with this function. In addition to the canonical substrate-binding face of the F-box proteins being engaged with the CPL scaffold, the CUL1-binding surface of SKP1 is either occluded or positioned such that CUL1 binding would clash with the surrounding CPL scaffold, as shown in Appendix Figure S1A. Consistent with this, we do not observe density corresponding to CUL1. We therefore conclude that CPL-scaffold-associated FBXW-SKP1 complexes are unlikely to recruit CUL1-containing Cullin-RING ligases, and instead appear to mediate interactions with other CPL components and contribute to the higher-order assembly of CPL filaments.

3. The authors should add a short discussion on the tubulin components part of the CPL. What is the nucleotide state? How do these tubulins differ between assembled or free tubulins?

We have now added this information to the Results section (lines 167-174; Figure EV4B,C). Specifically, we show that structural comparison with soluble tubulin and microtubule-incorporated tubulin dimers shows that CPL-bound αβ-tubulin adopts a conformation resembling soluble tubulin rather than tubulin within the microtubule lattice (Fig. EV4C). We also now indicate that the density is consistent with GTP bound at both canonical nucleotide-binding sites: the non-exchangeable N-site in α-tubulin, located at the α/β intradimer interface, and the exchangeable E-site in β-tubulin (Fig. EV4B). Finally, we clarify that, although the density was sufficient to distinguish α- from β-tubulin, the resolution was not sufficient to determine whether CPLs contain a single αβ-tubulin isoform pair or a mixture of different α- and β-tubulin isoforms. Overall, these additions indicate that CPLs incorporate a soluble-like, GTP-bound αβ-tubulin heterodimer as a stable architectural component of the repeat.

4. The author's observation of two related CPL states containing a ubiquitin-charged UBE2D complex or a rearranged CPL with no visible UB is an exciting observation. Considering the labile nature of E2~UB complexes it is incredible to visualize such a transient intermediate in situ. We kindly suggest the authors to cite the works of Juang et al. 2012 (PMID: 22325355) and Wiener et al. 2013 (PMID: 23955022) which observed an almost identical E2-UB conformation. The author's claim that the rearrangements indicate a form of regulated ubiquitin transfer are not supported by their data. Such

an activation mechanism cannot be drawn from purely structural data. It is also wholly unclear how the binding of an additional FBXW-SKP1 complex relates to the rearrangement of the CPL to seemingly "activate" the UBE2D-UB complex. In the absence of any functional data that would indicate ubiquitin transfer occurring as part of a CPL the authors should rephrase their conclusions.

We have added citations to Juang et al. (2012) and Wiener et al. (2013), noting that the open E2~ubiquitin conformation we observe closely resembles the conformation stabilised by OTUB1 described in those studies (Results, lines 308-310 and Fig. EV10B).

We fully agree that an activation mechanism cannot be inferred from structural data alone and have therefore rephrased our wording throughout the manuscript to address this concern.

In the Results, we now state explicitly that our structural data do not directly demonstrate ubiquitin transfer within CPLs (lines 330-334). We describe the two states of the CPL core complex, one containing ubiquitin and the other containing a central FBXW-SKP1 complex. What we do report is what we observe, namely that the CPL core complex can hold ubiquitin-charged UBE2D in a restrained open state, and can also adopt a conformation in which this restraint is absent. We do not imply that the central FBXW-SKP1 complex activates the UBE2D~Ub conjugate, and we now set out the alternative interpretations of the absence of ubiquitin density in this central FBXW-SKP1 state without asserting which applies: it could reflect increased flexibility of a still-tethered conjugate, loss of the conjugate, or transfer to an unknown acceptor.

In the Discussion, the proposal that CPLs act as ubiquitin ligase assemblies is now framed explicitly as a hypothesis. Our intended point, which we have made more carefully, is that CPLs possess the structural features required for ubiquitin transfer, namely ubiquitin-charged E2 enzymes, a mechanism for regulating ubiquitin availability, and RING domains positioned to promote the catalytically active state, but that whether ubiquitin transfer actually occurs remains an open question. The Discussion also now states that this model remains to be directly tested (lines 464-469).

We have also softened the Abstract accordingly, which now describes the rearrangements as compatible with ubiquitin becoming available for transfer rather than as evidence of regulated transfer.

Minor:

1. Instead of writing that UHRF1 is bound to "its known E2 ubiquitin-conjugating enzyme UBE2D". The authors should indicate that UHRF1 is bound to a member of the UBE2D-family as the resolution will now allow conclusive differentiation between members of the family.

We agree and have revised the sentence accordingly (see lines 155-156).

2. It would be of interest to the reader to include a short discussion of how the current structural work might inform the mechanisms of CPL disassembly.

We agree that the mechanisms underlying CPL disassembly are an interesting and important question. At present, however, the developmental stage at which CPLs disassemble remains unclear.

Our structure shows that CPLs are present in 8-cell-stage embryos and that the FBXW-SKP1 and UHRF1-UBE2D-NLRP14-Tubulin complexes remain integral components of the repeat unit at this stage. Any large-scale disassembly must therefore occur later in preimplantation development. Consistent with this, published proteomic data indicate that CPL components persist until the morula stage and remain abundant in blastocysts (Zhu et al., 2025; Appendix Figure S1D).

We have added this information to the Discussion of the revised manuscript (lines 407-411). Because our structure is derived from a single developmental stage, we have not speculated further on the disassembly mechanism. Defining this will require in situ structural analysis across later preimplantation stages, which our workflow is now well placed to enable.

3. In Figure 5a, the domain diagram for FBXW should be generalized by removing the residue numbers. As the authors cannot assign their FBXW densities to a specific FBXW protein the domain diagram should reflect this ambiguity.

We have removed the residue numbers from the FBXW domain diagram in Figure 5A to reflect the ambiguity in the precise FBXW identity.

## 1st Revision - Editorial Decision 24th Jul 2026

Dear Dr. Singh,

Thank you for submitting a revised version of your manuscript. I have now gone through your responses to the reviewers' comments and find them generally reasonable. Therefore, I am now pleased to inform you that your manuscript has been accepted for publication. Congratulations on a great study!

Before we forward your manuscript to the publishers, I still need from you a brief synopsis text consisting of a short (1-2 sentences) summary paragraph of the findings and their significance, followed by 3-4 bullet points highlighting key results. We also need a synopsis image that is 550x300 pixels large (width x height, jpeg or png format), which can show either a model of the findings or key data.

Your manuscript will be processed for publication by EMBO Press. It will be copy edited and you will receive page proofs prior to publication. Please note that you will be contacted by Springer Nature Author Services to complete licensing and payment information.

You may qualify for financial assistance for your publication charges - either via a Springer Nature fully open access agreement or an EMBO initiative. Check your eligibility: https://link.springer.com/journal/44318/how-to-publish-with-us

Should you be planning a Press Release on your article, please notify the production team via this form in order to coordinate publication and release dates: https://forms.cloud.microsoft/e/eH4A8JVP6g

I look forward to receiving the synopsis files from you soon. If you have any further questions, please do not hesitate to contact the Editorial Office or me directly. Thank you for this impressive contribution to The EMBO Journal!

Best wishes,

Ieva

---

Ieva Gailite, PhD
Senior Scientific Editor
The EMBO Journal
Meyerhofstrasse 1
D-69117 Heidelberg
Tel: +4962218891309
i.gailite@embojournal.org

*****

Please note that it is The EMBO Journal policy for the transcript of the editorial process (containing referee reports and your response letters) to be published as an online supplement to each paper. If you should prefer removal of any referee-only figures included in the point-by-point response(s), e.g. because they may still be used for future publication or because they have been reproduced from published work by others, please do let us know immediately via response email.
More information is available here: https://link.springer.com/partners/embo-press/editorial-policies#Peer%20review

## EMBO Press Author Checklist


| | |
|---|---|
| Corresponding Author Name: Kashish Singh | |
| Journal Submitted to: EMBO Journal | |
| Manuscript Number: EMBOJ-2026-124203 | |


**USEFUL LINKS FOR COMPLETING THIS FORM**
The EMBO Journal - Author Guidelines
EMBO Reports - Author Guidelines
Molecular Systems Biology - Author Guidelines
EMBO Molecular Medicine - Author Guidelines

**Reporting Checklist for Life Science Articles (updated January**
This checklist is adapted from Materials Design Analysis Reporting (MDAR) Checklist for Authors. MDAR establishes a minimum set of requirements in transparent reporting in the life sciences (see Statement of Task: 10.31222/osf.io/9sm4x). Please follow the journal's guidelines in preparing your manuscript.
**Please note that a copy of this checklist will be published alongside your article.**

**Abridged guidelines for figures**
**1. Data**
The data shown in figures should satisfy the following conditions:
- the data were obtained and processed according to the field's best practice and are presented to reflect the results of the experiments in an accurate and unbiased manner.
- ideally, figure panels should include only measurements that are directly comparable to each other and obtained with the same assay.
- plots include clearly labeled error bars for independent experiments and sample sizes. Unless justified, error bars should not be shown for technical
- if n<5, the individual data points from each experiment should be plotted. Any statistical test employed should be justified.
- Source Data should be included to report the data underlying figures according to the guidelines set out in the authorship guidelines on Data

**2. Captions**
Each figure caption should contain the following information, for each panel where they are relevant:
- a specification of the experimental system investigated (eg cell line, species name).
- the assay(s) and method(s) used to carry out the reported observations and measurements.
- an explicit mention of the biological and chemical entity(ies) that are being measured.
- an explicit mention of the biological and chemical entity(ies) that are altered/varied/perturbed in a controlled manner.
- the exact sample size (n) for each experimental group/condition, given as a number, not a range;
- a description of the sample collection allowing the reader to understand whether the samples represent technical or biological replicates (including how many animals, litters, cultures, etc.).
- a statement of how many times the experiment shown was independently replicated in the laboratory.
- definitions of statistical methods and measures:
  - common tests, such as t-test (please specify whether paired vs. unpaired), simple χ2 tests, Wilcoxon and Mann-Whitney tests, can be unambiguously identified by name only, but more complex techniques should be described in the methods section;
  - are tests one-sided or two-sided?
  - are there adjustments for multiple comparisons?
  - exact statistical test results, e.g., P values = x but not P values < x;
  - definition of 'center values' as median or average;
  - definition of error bars as s.d. or s.e.m.

**Please complete ALL of the questions below.**
**Select "Not Applicable" only when the requested information is not relevant for your study.**

### Materials

| Newly Created Materials | Information included in the manuscript? | In which section is the information available? (Reagents and Tools Table, Materials and Methods, Figures, Data Availability Section) |
|---|---|---|
| New materials and reagents need to be available; do any restrictions apply? | Not Applicable | |

| Antibodies | Information included in the manuscript? | In which section is the information available? (Reagents and Tools Table, Materials and Methods, Figures, Data Availability Section) |
|---|---|---|
| For **antibodies** provide the following information:<br>- Commercial antibodies: RRID (if possible) or supplier name, catalogue number and or/clone number<br>- Non-commercial: RRID or citation | Yes | Reagents and Tools Table , Materials and Methods sections |

| DNA and RNA sequences | Information included in the manuscript? | In which section is the information available? (Reagents and Tools Table, Materials and Methods, Figures, Data Availability Section) |
|---|---|---|
| **Short novel DNA or RNA including primers, probes**: provide the sequences. | Not Applicable | |

| Cell materials | Information included in the manuscript? | In which section is the information available? (Reagents and Tools Table, Materials and Methods, Figures, Data Availability Section) |
|---|---|---|
| **Cell lines:** Provide species information, strain. Provide accession number in repository **OR** supplier name, catalog number, clone number, and/**OR** RRID. | Not Applicable | |
| **Primary cultures:** Provide species, strain, sex of origin, genetic modification status. | Not Applicable | |
| Report if the cell lines were recently **authenticated** (e.g., by STR profiling) and tested for mycoplasma contamination. | Not Applicable | |

| Experimental animals | Information included in the manuscript? | In which section is the information available? (Reagents and Tools Table, Materials and Methods, Figures, Data Availability Section) |
|---|---|---|
| **Laboratory animals or Model organisms:** Provide species, strain, sex, age, genetic modification status. Provide accession number in repository **OR** supplier name, catalog number, clone number, **OR** RRID. | Yes | Reagents and Tools Table , Materials and Methods sections |
| **Animal observed in or captured from the field:** Provide species, sex, and age where possible. | Not Applicable | |
| Please detail **housing and husbandry conditions**. | Yes | Methods |

| Plants and microbes | Information included in the manuscript? | In which section is the information available? (Reagents and Tools Table, Materials and Methods, Figures, Data Availability Section) |
|---|---|---|
| **Plants:** provide species and strain, ecotype and cultivar where relevant, unique accession number if available, and source (including location for collected wild specimens). | Not Applicable | |
| **Microbes:** provide species and strain, unique accession number if available, and source. | Not Applicable | |

| Human research participants | Information included in the manuscript? | In which section is the information available? (Reagents and Tools Table, Materials and Methods, Figures, Data Availability Section) |
|---|---|---|
| If collected and within the bounds of privacy constraints report on age, sex and gender or ethnicity for all study participants. | Not Applicable | |

| Core facilities | Information included in the manuscript? | In which section is the information available? (Reagents and Tools Table, Materials and Methods, Figures, Data Availability Section) |
|---|---|---|
| If your work benefited from core facilities, was their service mentioned in the acknowledgments section? | Yes | Acknowledgments section |

### Design

| Study protocol | Information included in the manuscript? | In which section is the information available? (Reagents and Tools Table, Materials and Methods, Figures, Data Availability Section) |
|---|---|---|
| If study protocol has been **pre-registered, provide DOI in the manuscript**. For clinical trials, provide the trial registration number **OR** cite DOI. | Not Applicable | |
| Report the **clinical trial registration number** (at ClinicalTrials.gov or equivalent), where applicable. | Not Applicable | |

| Laboratory protocol | Information included in the manuscript? | In which section is the information available? (Reagents and Tools Table, Materials and Methods, Figures, Data Availability Section) |
|---|---|---|

| | | |
|---|---|---|
| Provide DOI OR other citation details if **external detailed step-by-step protocols** are available. | Not Applicable | |

| Experimental study design and statistics | Information included in the manuscript? | In which section is the information available? (Reagents and Tools Table, Materials and Methods, Figures, Data Availability Section) |
|---|---|---|
| Include a statement about **sample size** estimate even if no statistical methods were used. | Yes | Cryo-electron tomography data: no groups of conditions were compared to each other, and therefore, no sample-size calculation was performed. Figures 1 and EV2: No statistical methods were used to predetermine sample size. The analyses were descriptive, and no statistical comparisons between groups or conditions were performed. Sample size was dictated by the number of fertilized embryos obtained from natural timed matings of CD-1 (outbred) mice. |
| Were any steps taken to minimize the effects of subjective bias when allocating animals/samples to treatment (e.g. **randomization procedure**)? If yes, have they been described? | Yes | Cryo-electron tomography experiments did not have different treatments or conditions that could be randomized. Figures 1 and EV2: Mouse embryos were collected from multiple animals and then pooled. Embryos were then randomly assigned to different experimental groups. |
| Include a statement about **blinding** even if no blinding was done. | Yes | Cryo-electron tomography experiments: no groups of conditions were compared to each other, and therefore, these experiments did not require blinding. Figure 1 and EV2: Investigators were not blinded to allocation during experiments and outcome assessment. |
| Describe **inclusion/exclusion criteria** if samples or animals were excluded from the analysis. Were the criteria pre-established?<br><br>If sample or data points were omitted from analysis, report if this was due to attrition or intentional exclusion and provide justification. | Yes | Figure 1 and EV2: No data were excluded from the analysis. |
| For every figure, are **statistical tests** justified as appropriate? Do the data meet the assumptions of the tests (e.g., normal distribution)? Describe any methods used to assess it. Is there an estimate of variation within each group of data? Is the variance similar between the groups that are being statistically compared? | Not Applicable | |

| Sample definition and in-laboratory replication | Information included in the manuscript? | In which section is the information available? (Reagents and Tools Table, Materials and Methods, Figures, Data Availability Section) |
|---|---|---|
| In the figure legends: state number of times the experiment was **replicated** in laboratory. | Yes | Figure legends |
| In the figure legends: define whether data describe **technical or biological replicates**. | Yes | Independent replicates refer to biological replicates. Each replicate consisted of embryos pooled from several naturally mated outbred mice, and experiments were performed independently on different days using distinct embryo collections. |

## Ethics

| Ethics | Information included in the manuscript? | In which section is the information available? (Reagents and Tools Table, Materials and Methods, Figures, Data Availability Section) |
|---|---|---|
| Studies involving **human participants**: State details of **authority granting ethics approval** (IRB or equivalent committee(s), provide reference number for approval. | Not Applicable | |
| Studies involving **human participants**: Include a statement confirming that **informed consent** was obtained from all subjects and that the experiments conformed to the principles set out in the WMA Declaration of Helsinki and the Department of Health and Human Services Belmont Report. | Not Applicable | |
| Studies involving **human participants:** For publication of **patient photos**, include a statement confirming that consent to publish was obtained. | Not Applicable | |
| Studies involving experimental **animals**: State details of **authority granting ethics approval** (IRB or equivalent committee(s), provide reference number for approval. Include a statement of compliance with ethical regulations. | Yes | Animal work was carried out in accordance with UK legislation. Naturally mated CD-1 female mice were maintained by the institutional animal facility and were humanely culled by trained personnel using an approved Schedule 1 method under the Animals (Scientific Procedures) Act 1986. Preimplantation embryos were collected post mortem for analysis. No regulated procedures were performed on live animals as part of this study, and therefore no UK Home Office project licence was required for the embryo collection described here. |
| Studies involving **specimen and field samples:** State if relevant **permits** obtained, provide details of authority approving study; if none were required, explain why. | Not Applicable | |

| Dual Use Research of Concern (DURC) | Information included in the manuscript? | In which section is the information available? (Reagents and Tools Table, Materials and Methods, Figures, Data Availability Section) |
|---|---|---|
| Could your study fall under dual use research restrictions? Please check biosecurity documents and list of **select agents and toxins** (CDC): https://www.selectagents.gov/sat/list.htm | Not Applicable | |
| If you used a select agent, is the security level of the lab appropriate and reported in the manuscript? | Not Applicable | |
| If a study is subject to dual use research of concern regulations, is the name of the **authority granting approval and reference number** for the regulatory approval provided in the manuscript? | Not Applicable | |

## Reporting

The MDAR framework recommends adoption of discipline-specific guidelines, established and endorsed through community initiatives. Journals have their own policy about requiring specific guidelines and recommendations to complement MDAR.

| Adherence to community standards | Information included in the manuscript? | In which section is the information available? (Reagents and Tools Table, Materials and Methods, Figures, Data Availability Section) |
|---|---|---|
| State if relevant guidelines or checklists (e.g., **ICMJE, MIBBI, ARRIVE, PRISMA**) have been followed or provided. | Not Applicable | Animal use was limited to the post-mortem collection of preimplantation embryos following Schedule 1 culling, with no regulated procedures on live animals. The applicable ARRIVE reporting items (species, strain, sex, source and husbandry) are reported in the Methods section. Items relating to experimental group design (randomisation, blinding, sample-size determination, group allocation) are not applicable to this study. |
| For **tumor marker prognostic studies,** we recommend that you follow the **REMARK** reporting guidelines (see link list at top right). See author guidelines, under 'Reporting Guidelines'. Please confirm you have followed these guidelines. | Not Applicable | |
| For **phase II and III randomized controlled trials**, please refer to the **CONSORT** flow diagram (see link list at top right) and submit the CONSORT checklist (see link list at top right) with your submission. See author guidelines, under 'Reporting Guidelines'. Please confirm you have submitted this list. | Not Applicable | |

## Data Availability

| Data availability | Information included in the manuscript? | In which section is the information available? (Reagents and Tools Table, Materials and Methods, Figures, Data Availability Section) |
|---|---|---|
| Have **primary datasets** been deposited according to the journal's guidelines (see 'Data Deposition' section) and the respective accession numbers provided in the Data Availability Section? | Yes | Data and materials availability section |
| Were **human clinical and genomic datasets** deposited in a public access-controlled repository in accordance to ethical obligations to the patients and to the applicable consent agreement? | Not Applicable | |
| Are **computational models** that are central and integral to a study available without restrictions in a machine-readable form? Were the relevant accession numbers or links provided? | Not Applicable | |
| If publicly available data were reused, provide the respective **data citations in the reference list**. | Yes | Data from the supplementary table of Zhu et al (2025) were reanalysed and the study has been cited accordingly in the discussion section. |
