# Supplementary material for: In-situ cryo-ET of mouse embryos reveals cytoplasmic lattices contain ubiquitin-charged E2-E3 ligase assemblies
Source: EMBO J. 2026 Aug 10;45(21):7405–37. doi: 10.1038/s44318-026-00895-w (PMC13728194; doi:10.1038/s44318-026-00895-w)
Supplement: Supplementary file 2 [file 44318_2026_895_MOESM2_ESM.pdf]

Appendix for

In-situ cryo-ET of mouse embryos reveals cytoplasmic lattices contain ubiquitin-charged E2-E3 ligase assemblies

Table of Contents

Appendix Fig. S1. Comparisons with other structures. ....……………………………………………p. 2-3

Appendix Fig. S2. AlphaFold3 predictions used as starting models for PADI6 and SCMC components……
…………………………………………………………………………………….……………………...p. 4

Appendix Fig. S3. AlphaFold3 predictions used as starting models for UHRF1-UBE2D-NLRP14-Tubulin complex and FBXW-SKP1 complex...p. 5

Appendix Table S1. Cryo-ET data collection and model refinement statistics.. p. 6

Appendix Table S2. Summary of model building for CPL components……………………………..........p. 7

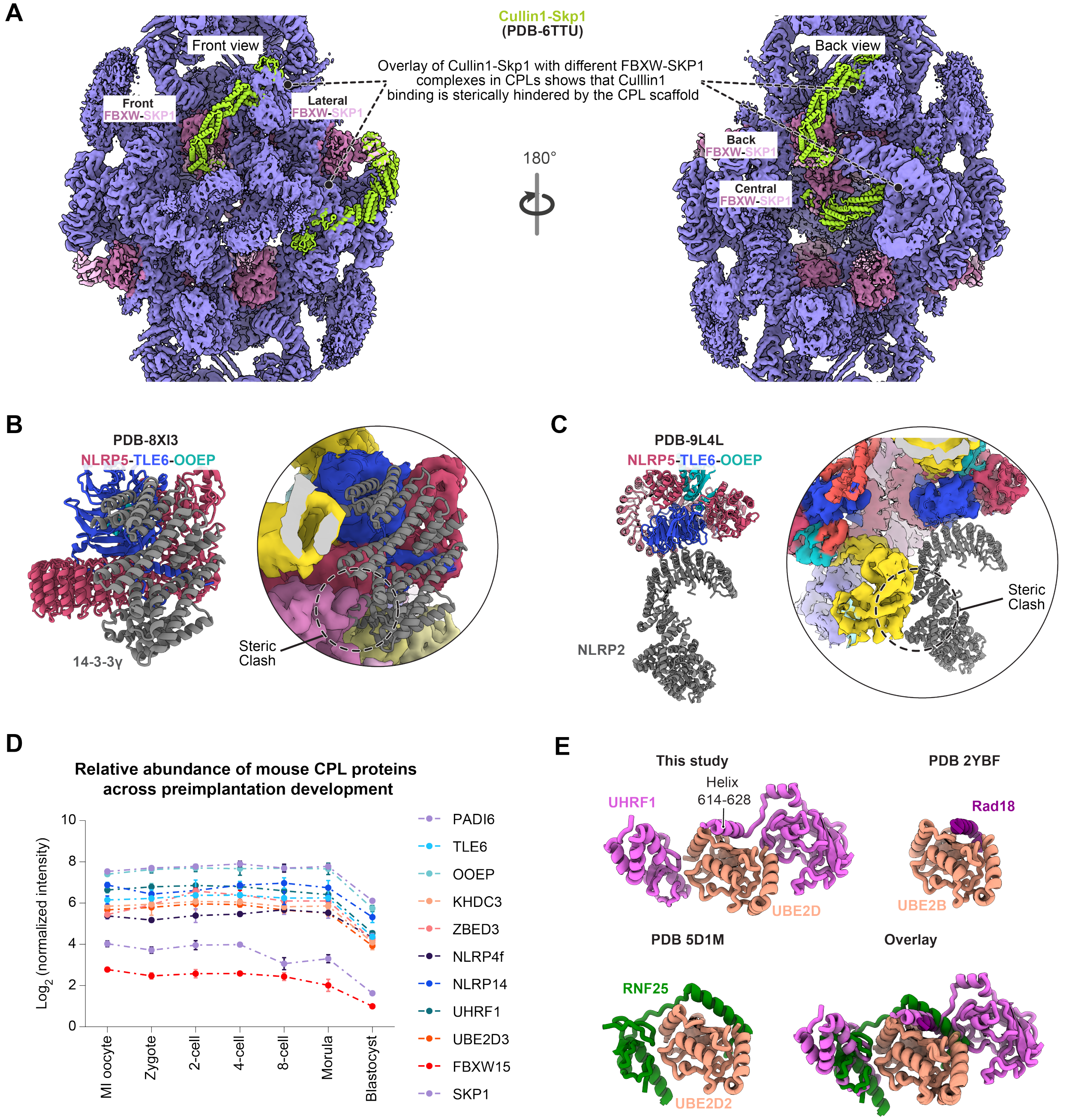

Appendix Figure S1: Comparisons with other structures. (A) Overlay of the CUL1-SKP1 complex (green, PDB-6TTU) onto the FBXW-SKP1 complexes within the CPL repeat unit, generated by aligning the SKP1 molecules. Front-face (left) and back-face (right) views are shown. At all four types of FBXW-SKP1 binding sites, CUL1 binding is sterically hindered by the CPL scaffold, indicating that CPL-associated FBXW-SKP1 complexes are unlikely to assemble into canonical SCF Cullin-RING ligases. **(B,C)** Structural comparison of previously reported complexes containing the NLRP5-TLE6-OOEP complex bound to 14-3-3 protein or NLRP2 with the CPL structure. Superposition shows that incorporation of these factors into the CPL repeat would result in steric clashes, indicating that these interactions are incompatible with the assembled CPL architecture. **(D)** Relative abundance of CPL-associated proteins in mouse oocytes and preimplantation embryos from the metaphase I (MI) oocyte to blastocyst stage. Values are shown as log_2-normalized protein intensity. Data were replotted from (Zhu et al, 2025). **(E)** Comparison of backside

interactions between E2 and E3 ligases. In the CPL structure, UHRF1 residues 614-628 form a helix that engages the backside surface of UBE2D. Similar helical backside interactions are observed in other E2-E3 complexes (PDB-2YBF (Hibbert et al, 2011) and PDB-5D1M (Li et al, 2015)) and are associated with promoting monoubiquitination or short ubiquitin chain formation.

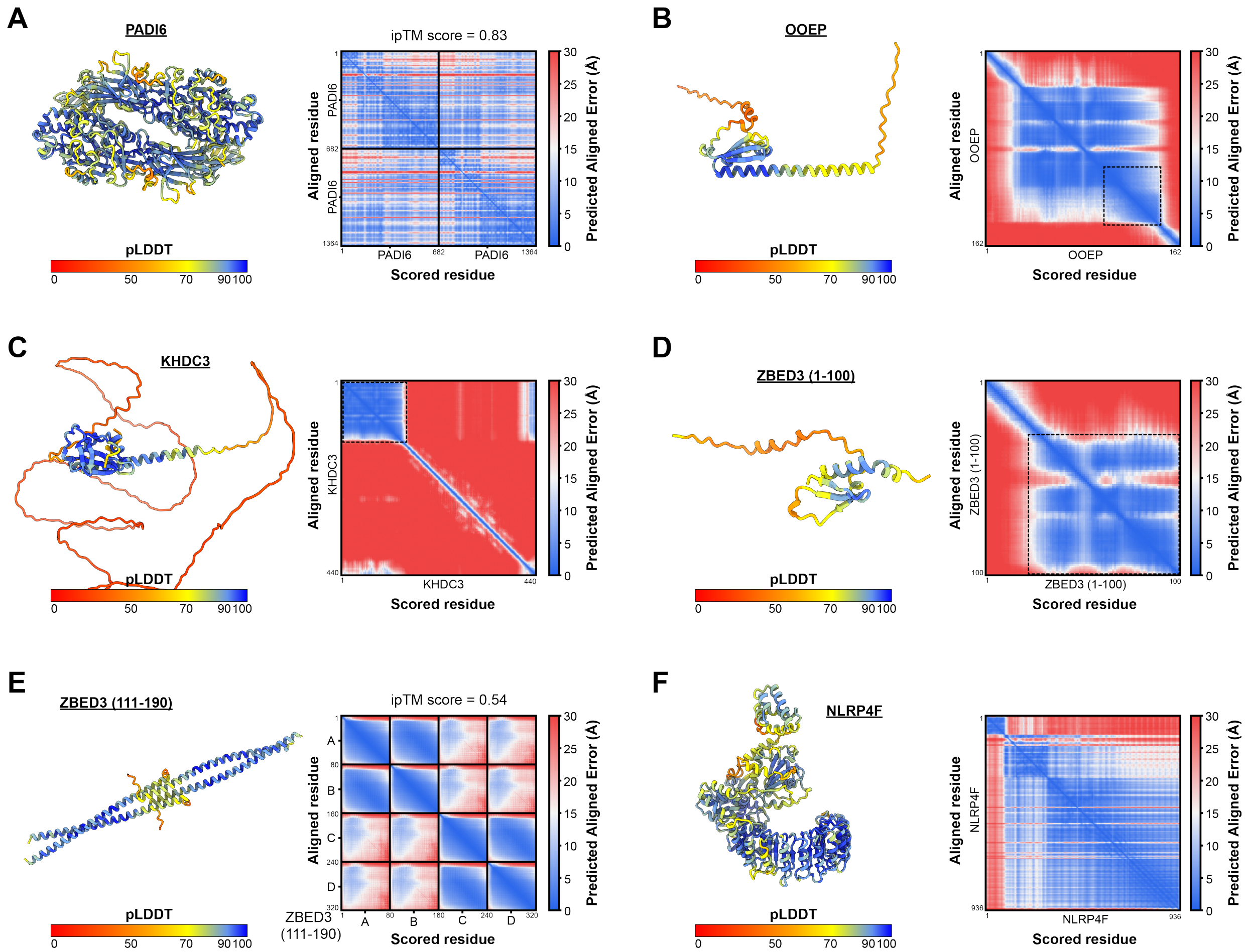

Appendix Figure S2: AlphaFold3 predictions used as starting models for PADI6 and SCMC components. (A) PADI6 dimer **(B)** OOEP **(C)** KHDC3 **(D)** ZBED3 N-terminal zinc-finger domain (residues 1-100) **(E)** ZBED3 C-terminal region (residues 111-190) predicted as a tetramer **(F)** NLRP4F. For each component, the predicted model is shown coloured by pLDDT (left) alongside its predicted aligned error (PAE) plot (right). pLDDT is coloured from red (low confidence) to blue (high confidence), and PAE from blue (low error) to red (high error), according to the scales shown. For multi-chain predictions, the ipTM score is indicated above the PAE plot and chain boundaries are marked. Where only a subregion of a protein was used as a starting model, the residue range is boxed on the PAE plot (dashed outline).

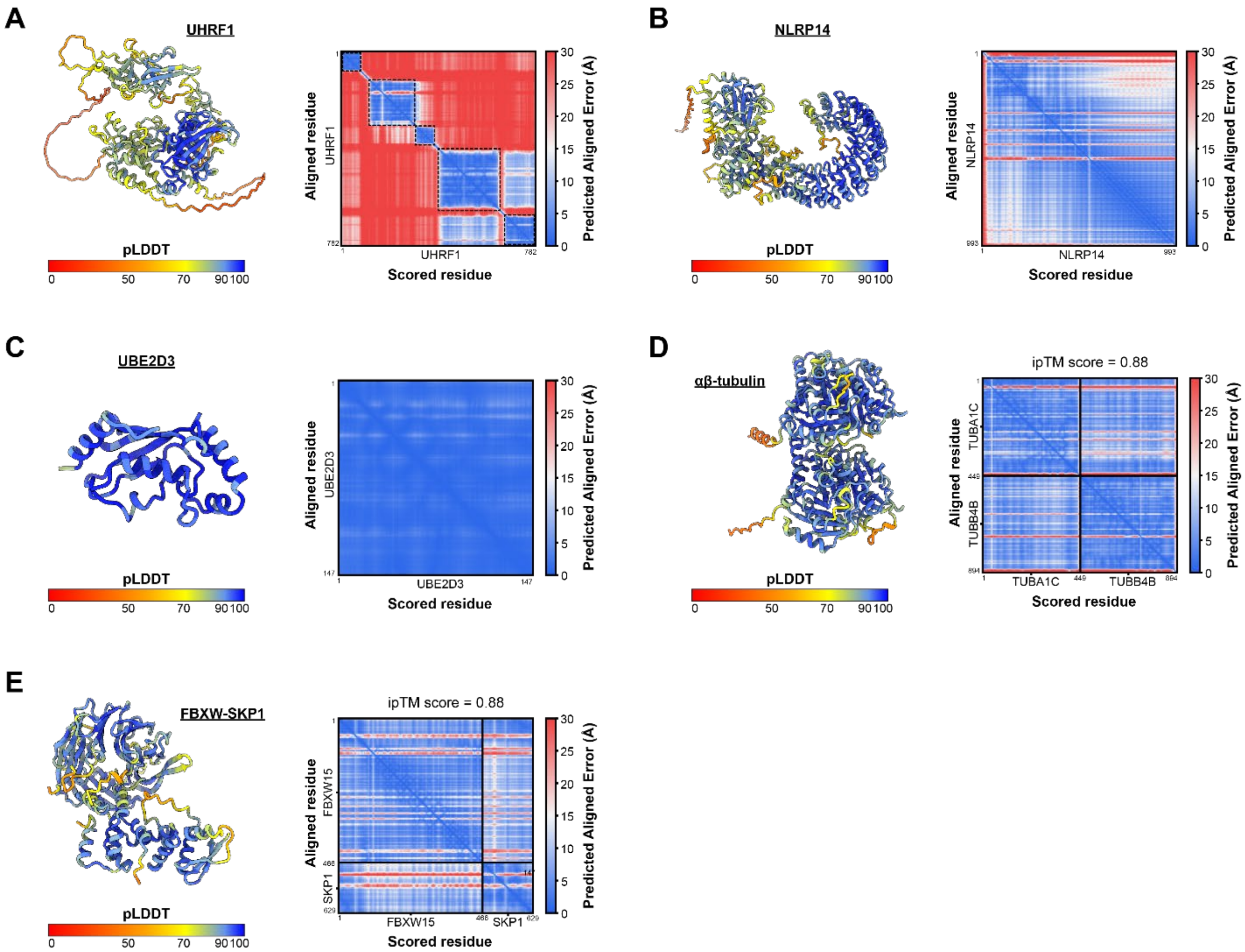

Appendix Figure S3: AlphaFold3 predictions used as starting models for UHRF1-UBE2D-NLRP14-Tubulin complex and FBXW-SKP1 complex. (A) UHRF1 **(B)** NLRP14 **(C)** UBE2D3 **(D)** TUBA1C-TUBB4B complex **(E)** FBXW15-SKP1 complex. For each component, the predicted model is shown coloured by pLDDT (left) alongside its predicted aligned error (PAE) plot (right). pLDDT is coloured from red (low confidence) to blue (high confidence), and PAE from blue (low error) to red (high error), according to the scales shown. For multi-chain predictions, the ipTM score is indicated above the PAE plot and chain boundaries are marked. Where only a subregion of a protein was used as a starting model, the residue range is boxed on the PAE plot (dashed outline).

Sample	Asymmetric unit of a CPL repeat	CPL repeat unit with central FBXW-SKP1	CPL repeat unit with ubiquitin	Inter-repeat-link	Inter-filament -link	Composite model of a CPL repeat unit	CPL repeat unit from intact embryos
**EMDB ID**	55603	55604	55607	55606	55605	55609	55608
**PDB ID**	9T63	9T64	9T67	9T66	9T65	9T68	-
**Data collection and processing**							
No. of Tomograms	1,153						36
Magnification	105000X						53000x
Voltage	300						300
Pixel size (Å)	1.185						1.63
Exposure (e^-/ $Å^2$) per tilt (total tilts)	3.5 (33)						3.5 (33)
Frames collected	8						6
Defocus range (μm)	2-4.5						2.5-4.5
Final particle no.	268774	34451	30490	184196	86696	-	7595
Symmetry imposed	C1	C1	C1	C1	C1	C1	C1
Map Resolution (Å)	4.7	7.3	7.7	5.3	5.6	-	10
FSC threshold	0.143	0.143	0.143	0.143	0.143	-	0.143
Sharpening B factor ($Å^2$)	-180	-200	-200	-150	-100	-	-200
**Model Refinement**							
Model Resolution (Å)	6.0	7.8	8.2	6.3	6.5	-	-
FSC threshold	0.5	0.5	0.5	0.5	0.5	-	-
**Fit to map**							
CC (entire box)	0.67	0.76	0.76	0.76	0.76	0.86	-
CC (around atoms)	0.77	0.71	0.70	0.81	0.8	0.67	-
**Model composition**							
Non-hydrogen atoms	76879	183153	181130	89612	35990	270782	-
Protein residues	15532	37008	36601	18104	7271	54715	-
**Mean B factors ($Å^2$)**							
Protein	206	239	293	264	257	169	-
**RMSD deviations**							
Bond lengths (Å)	0.004	0.005	0.004	0.005	0.005	0.004	-
Bond angles (°)	1.131	1.137	1.119	1.135	1.132	1.142	-
**Validation**							
Clashscore	1.17	3.66	2.57	1.73	2.24	1.53	-
MolProbity score	1.27	1.66	1.52	1.41	1.46	1.36	-
**Ramachandran plot**							
Favored (%)	93.21	91.39	92.36	92.2	92.78	92.64	-
Allowed (%)	6.65	8.47	7.51	7.6	7.07	7.17	-
Outliers (%)	0.14	0.14	0.13	0.2	0.15	0.19	-

Appendix Table S1: Cryo-ET data collection and model refinement statistics.

Protein	Uniprot ID	Length	Molecular weight (kDa)	Modelled residues	Copies per repeat	Starting model
PADI6	Q8K3V4	682	76.78	1-682	24	AlphaFold3 prediction of PADI6 dimer
TLE6	Q9WVB3	581	65.116	146-177, 247-581	4	PDB-8H93
OOEP	Q9CWE6	164	18.46	26-144	4	PDB-8H93; AlphaFold3 prediction was used to model the helical region after the KH domain.
NLRP5	Q9R1M5	1163	131.32	97-470,485-1059	4	PDB-8H93
KHDC3	Q9CWU5	440	47.99	1-136	2	AlphaFold3
ZBED3	Q9D0L1	228	25.6	33-95,111-190	4	AlphaFold3 prediction of ZBED3 tetramer
NLRP4F	L7N1W9	937	107.87	1-90,93-937	2	AlphaFold3
NLRP14	Q6B966	993	113.39	28-993	3	AlphaFold3
UHRF1	Q8VDF2	782	88.3	1-76,123-305,309-372,416-629, 666-782	3	AlphaFold3
UBE2D3	P61079	147	16.69	1-147	3	AlphaFold3
α-tubulin	P68373 (Tuba1c)	449	49.9	1-443	3	AlphaFold3
β-tubulin	P68372 (Tubb4b)	445	49.8	1-440	3	AlphaFold3
FBXW	-	~466	~53.59	1-466	6-7	AlphaFold3 prediction of FBXW15-SKP1 complex
SKP1	Q9WTX5	163	18.67	1-163	6-7	AlphaFold3 prediction of FBXW15-SKP1 complex
Ubiquitin	P0CG50	76	8.64	1-74	0-3	PDB-6TTU

Appendix Table S2: Summary of model building for CPL components.
