# Supplementary material for: In-situ cryo-ET of mouse embryos reveals cytoplasmic lattices contain ubiquitin-charged E2-E3 ligase assemblies
Source: EMBO J. 2026 Aug 10;45(21):7405–37. doi: 10.1038/s44318-026-00895-w (PMC13728194; doi:10.1038/s44318-026-00895-w)
Supplement: Supplementary file 3 [file 44318_2026_895_MOESM7_ESM.pdf]

Expanded View Figures

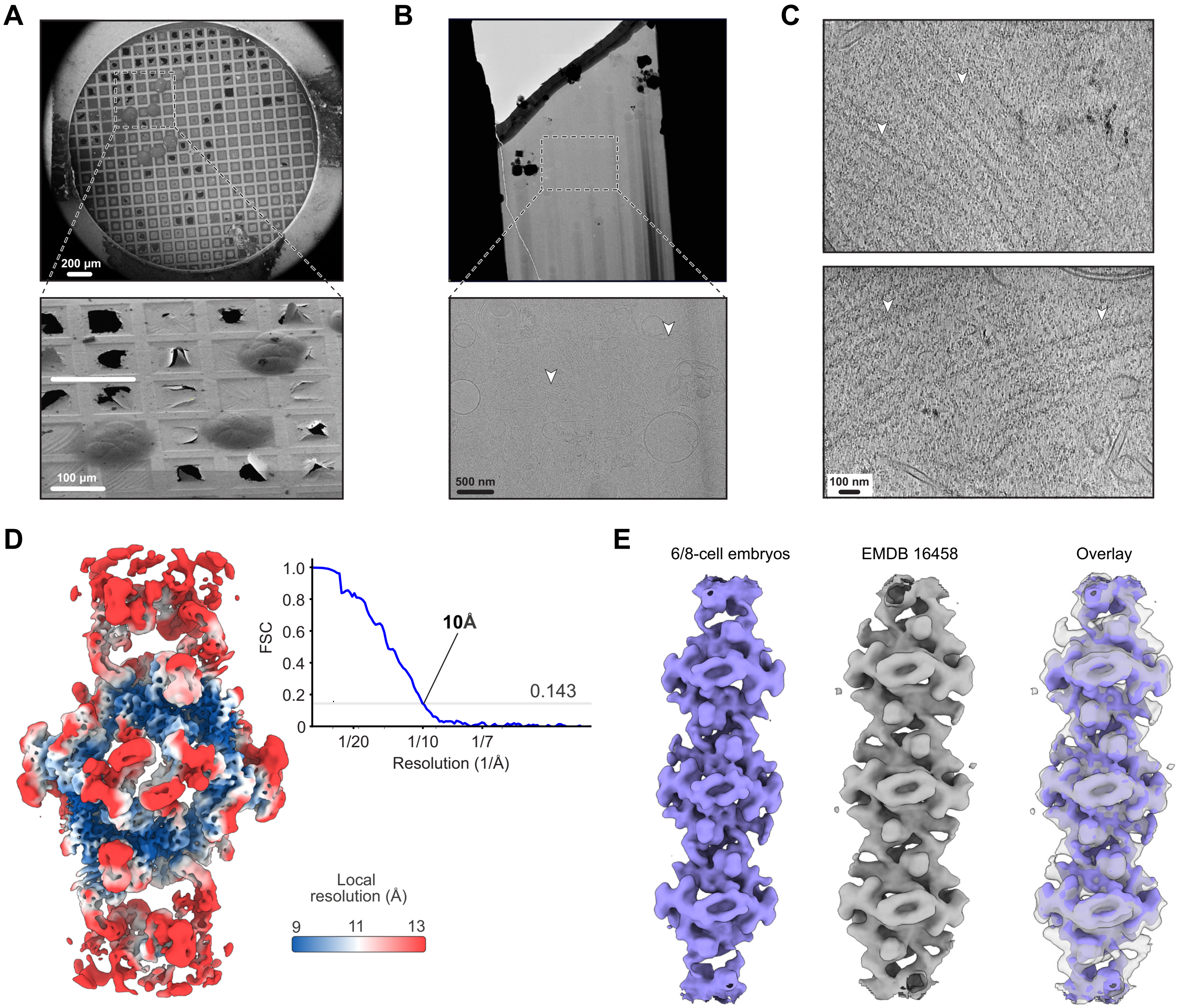

Figure EV1. Structure of CPL filaments in 6/8-cell mouse embryos.

(**A**) Scanning electron microscope (SEM) image of vitrified 6/8-cell mouse embryos on EM grids (top), and a FIB image showing three embryos on the grid (bottom). (**B**) Low-magnification transmission electron microscope (TEM) image of a representative lamella (top) and a zoomed-in view highlighting a few examples of CPLs with white arrowheads (bottom). (**C**) Representative denoised tomographic slices showing CPLs with a few examples highlighted with white arrowheads. (**D**) Subtomogram averaging map of the CPL repeat unit from cryo-ET data acquired on a lamella of intact mouse embryos, colored by local resolution. The corresponding Fourier shell correlation (FSC) curve is shown. (**E**) Comparison of CPL filament structure obtained using subtomogram averaging in early mouse embryos (left; this study) and from oocytes (center; EMDB-16458 (Jentoft et al, 2023)). The two maps are overlaid (right), with EMDB-16458 rendered semi-transparent.

 © The Author(s)

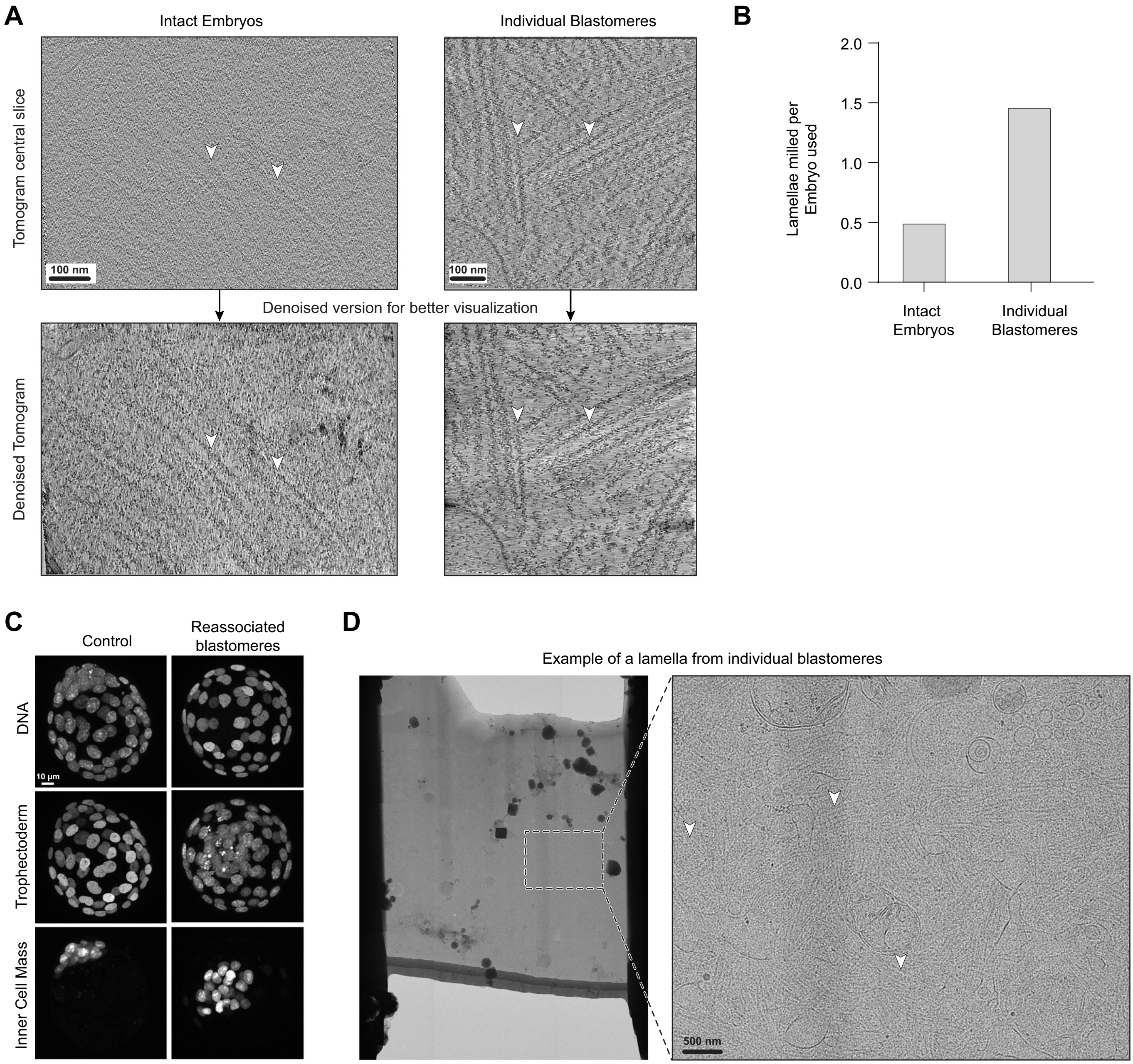

Figure EV2. Cryo-ET of 6/8-cell embryo blastomeres.

(**A**) Central slices of tomograms without and with denoising showing the improvement in contrast in data collected from individual blastomeres (right) as compared to that from intact embryos (left). Both tomograms were collected at a defocus of 4 µm and a lamella thickness of 130–140 nm. (**B**) Graphs comparing the total number of lamellae obtained relative to the number of embryos used for vitrification on EM grids, for intact embryos (left) and embryos separated into individual blastomeres (right). Not all vitrified embryos/blastomeres yielded lamellae, as a fraction were lost during vitrification or located in regions of the grid unsuitable for cryo-FIB milling.
(**C**) Immunofluorescence images showing the individual channels corresponding to the merged image in Fig. 1D. (**D**) Low-magnification TEM image of a representative lamella prepared from a blastomere (left) and a zoomed-in view highlighting few examples of CPLs with white arrowheads (right).

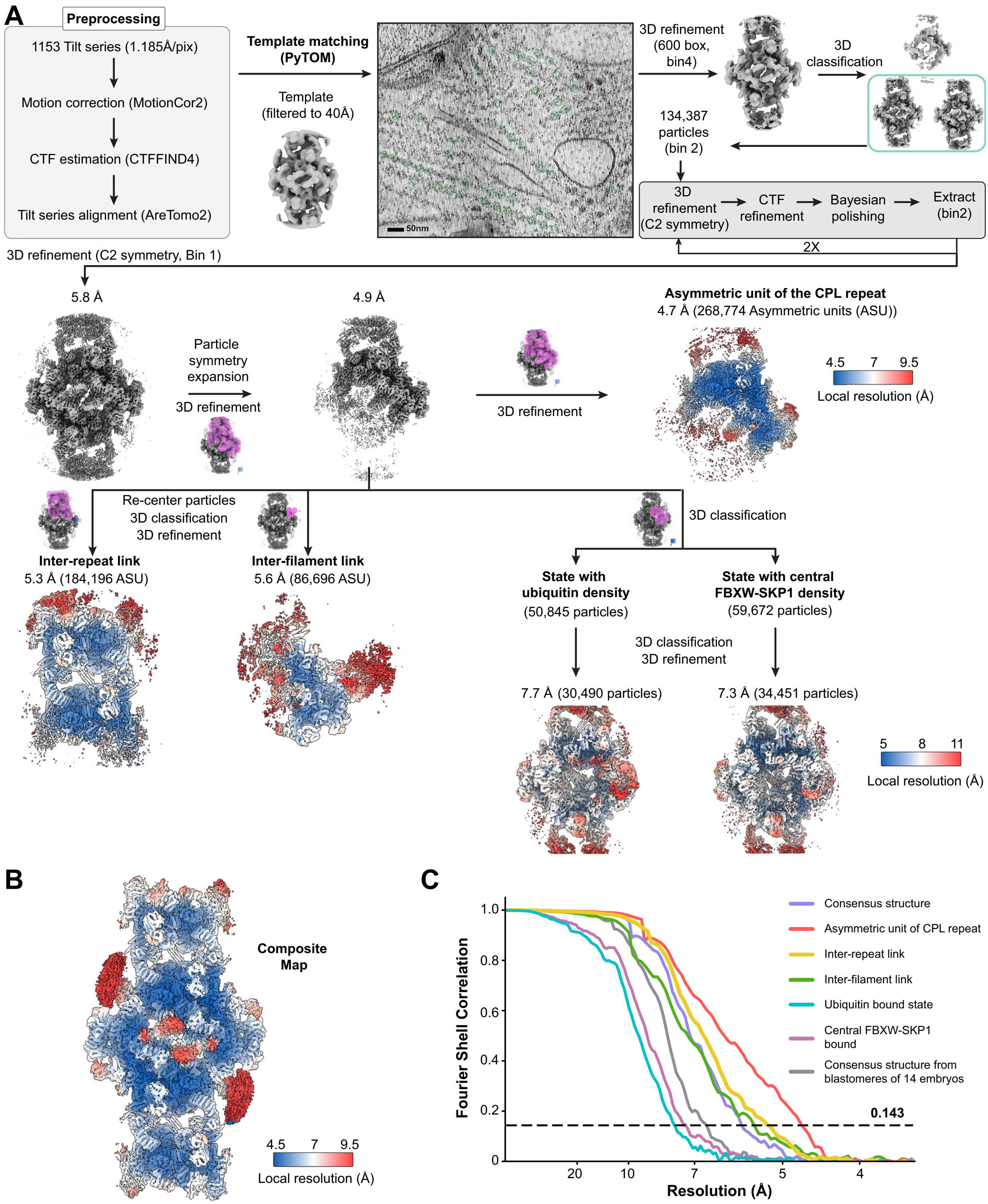
A
Preprocessing
1153 Tilt series (1.185Å/pix)
Motion correction (MotionCor2)
CTF estimation (CTFFIND4)
Tilt series alignment (AreTomo2)
Template matching (PyTOM)
Template (filtered to 40Å)
50nm
3D refinement (600 box, bin4)
3D classification
134,387 particles (bin 2)
3D refinement (C2 symmetry)
CTF refinement
Bayesian polishing
Extract (bin2)
2X
3D refinement (C2 symmetry, Bin 1)
5.8 Å
Particle symmetry expansion
3D refinement
4.9 Å
3D refinement
Asymmetric unit of the CPL repeat
4.7 Å (268,774 Asymmetric units (ASU))
4.5 7 9.5
Local resolution (Å)
Re-center particles
3D classification
3D refinement
Inter-repeat link
5.3 Å (184,196 ASU)
Inter-filament link
5.6 Å (86,696 ASU)
3D classification
State with ubiquitin density
(50,845 particles)
State with central FBXW-SKP1 density
(59,672 particles)
3D classification
3D refinement
7.7 Å (30,490 particles)
7.3 Å (34,451 particles)
5 8 11
Local resolution (Å)
B
Composite Map
4.5 7 9.5
Local resolution (Å)
C
Fourier Shell Correlation
1.0
0.8
0.6
0.4
0.2
0
0.143
20 10 7 5 4
Resolution (Å)
Consensus structure
Asymmetric unit of CPL repeat
Inter-repeat link
Inter-filament link
Ubiquitin bound state
Central FBXW-SKP1 bound
Consensus structure from blastomeres of 14 embryos

 © The Author(s)

◀ **Figure EV3. Subtomogram averaging processing pipeline.**

(**A**) The workflow for resolving the structure of the CPL repeat is shown. (**B**) The composite map generated by combining maps obtained after focused 3D refinements is depicted and colored based on local resolution. (**C**) Plot shows the gold-standard Fourier Shell Correlation of the different subtomogram averaging maps shown in (**A**). The dotted horizontal line shows the 0.143 cut-off.

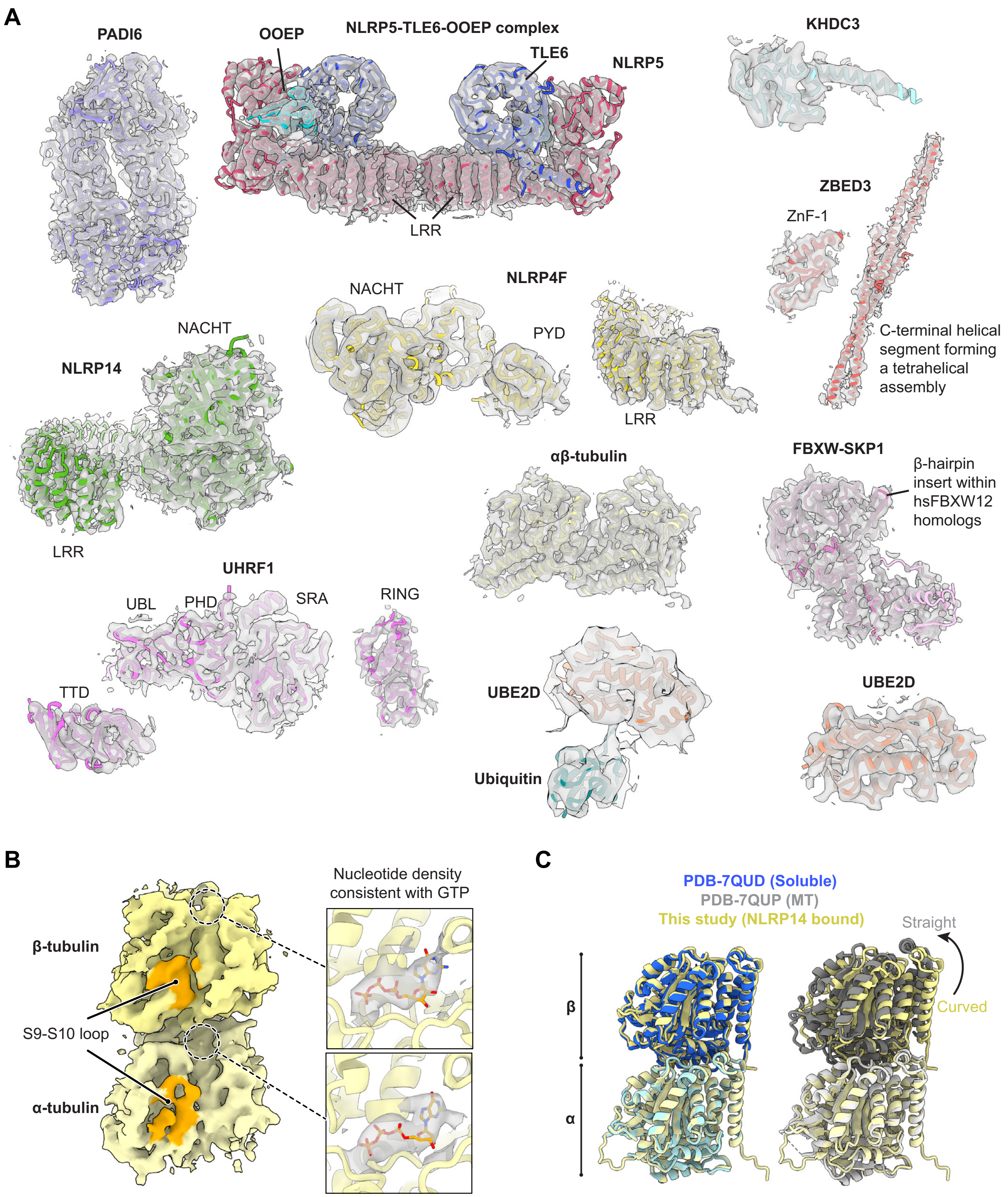
A
PADI6
OOEP
NLRP5-TLE6-OOEP complex
TLE6
NLRP5
LRR
KHDC3
ZBED3
ZnF-1
C-terminal helical segment forming a tetrahelical assembly
NACHT
NLRP4F
PYD
LRR
NACHT
NLRP14
LRR
αβ-tubulin
FBXW-SKP1
β-hairpin insert within hsFBXW12 homologs
UHRF1
UBL
PHD
SRA
RING
TTD
UBE2D
Ubiquitin
UBE2D
B
Nucleotide density consistent with GTP
β-tubulin
S9-S10 loop
α-tubulin
C
PDB-7QUD (Soluble)
PDB-7QUP (MT)
This study (NLRP14 bound)
Straight
Curved
β
α

© The Author(s)

Figure EV4. Densities of proteins identified within CPLs.

(**A**) Gallery showing individual proteins with their atomic models fitted into the corresponding subtomogram averaging density maps. (**B**) Density of the αβ-tubulin showing the S9–S10 loop in orange. The loop is shorter in the density assigned to β-tubulin, as expected. Insets show the modeled GTP in the corresponding density at the N-site (α/β intradimer interface; bottom) and the E-site (β-tubulin; top). (**C**) Structural comparison of αβ-tubulin from the CPL repeat with soluble (PDB 7QUD (Wagstaff et al, 2023)) and microtubule-incorporated tubulin dimers (PDB 7QUP (Wagstaff et al, 2023)), showing that CPL-bound αβ-tubulin adopts a conformation resembling the soluble form.

© The Author(s)

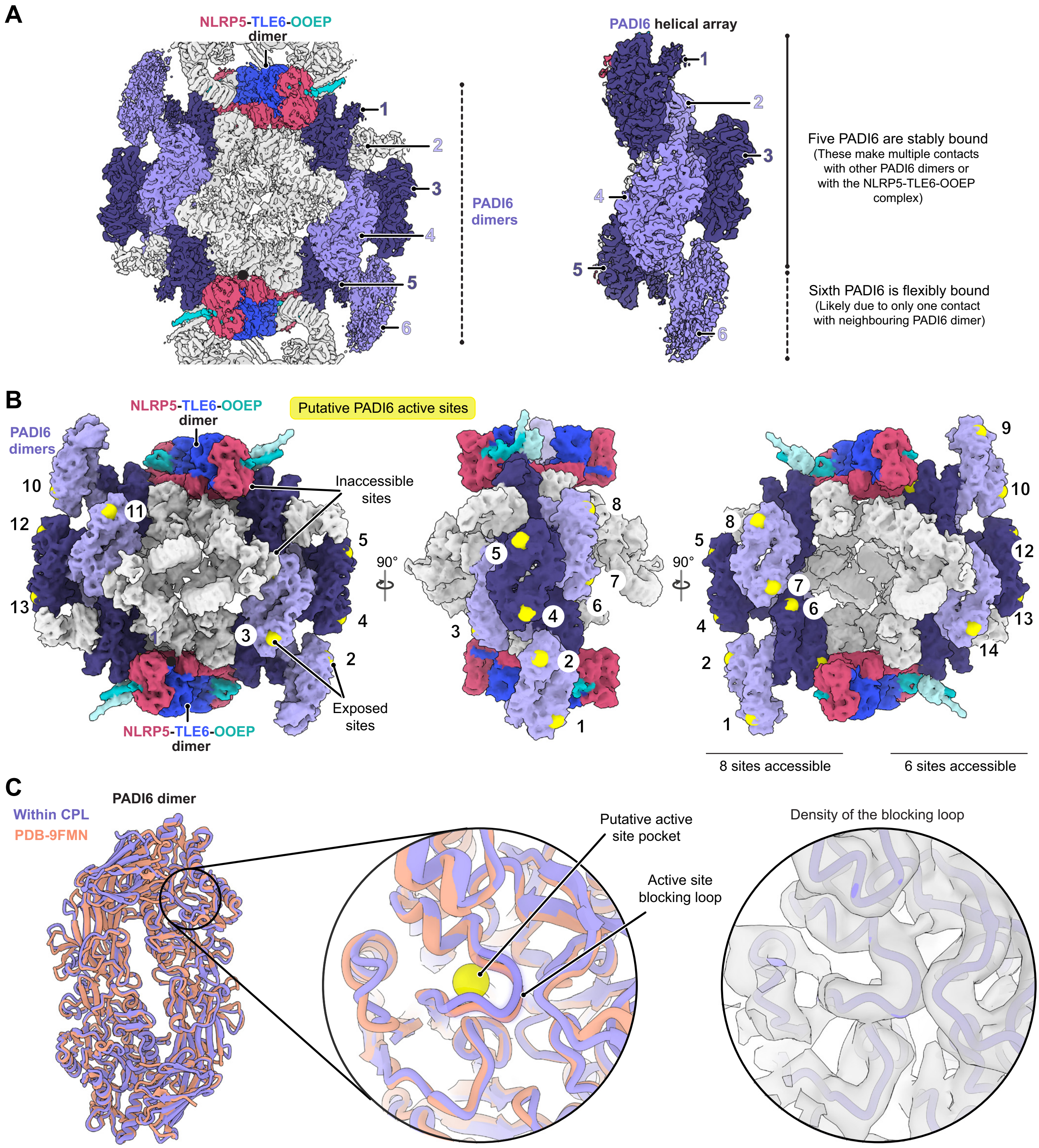

Figure EV5. Arrangement of putative catalytic sites of PADI6 within CPLs.

(**A**) Subtomogram averaging density of the PADI6 helical array is shown. Five PADI6 dimers (1–5) are stably bound, whereas the sixth PADI6 dimer is more flexibly attached as its corresponding density is resolved at a lower resolution. (**B**) Putative catalytic sites of PADI6 within a CPL repeat unit are depicted as yellow spheres. A total of 14 sites are exposed, whereas 10 sites are occluded by the interactions of PADI6 with the NLRP5–TLE6–OOEP complex or with the proteins bound to the central scaffold (shown in light gray). (**C**) The PADI6 dimer within the CPL helical array is compared with the structure of the isolated PADI6 dimer (PDB-9FMN) (Williams et al, 2024). In both cases, the putative catalytic pocket is occluded. The subtomogram averaging density corresponding to the blocking loop is shown on the right.

 © The Author(s)

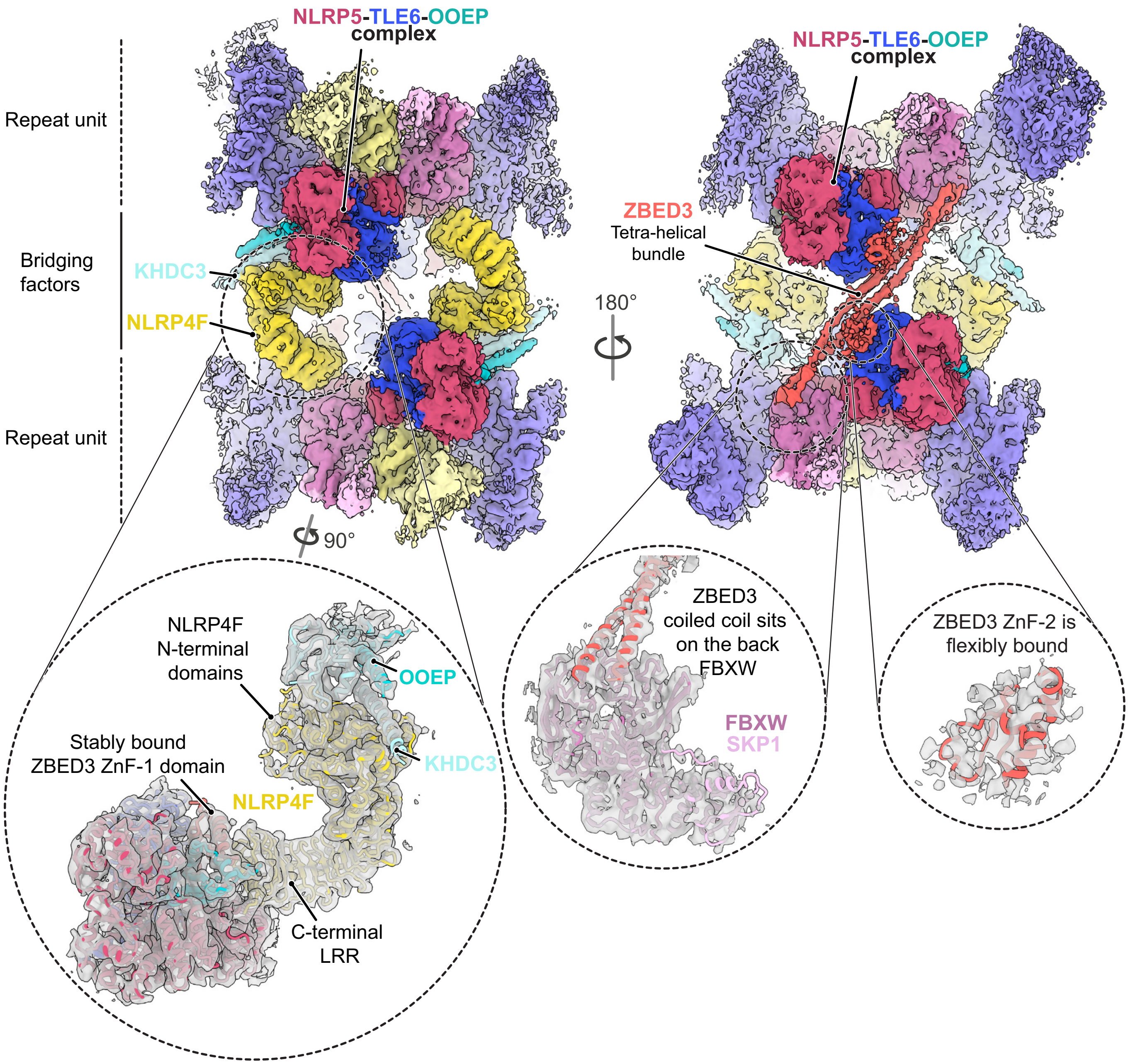

Figure EV6. Subtomogram averaging density linking two CPL repeat units.

Proteins mediating inter-repeat connections are labeled. Insets highlight NLRP4F, the interaction of the ZBED3 coiled-coil with a corner FBXW–SKP1 complex on the back face of the filament, and density corresponding to the flexibly attached ZBED3 zinc-finger (ZnF-2) domain bound to TLE6.

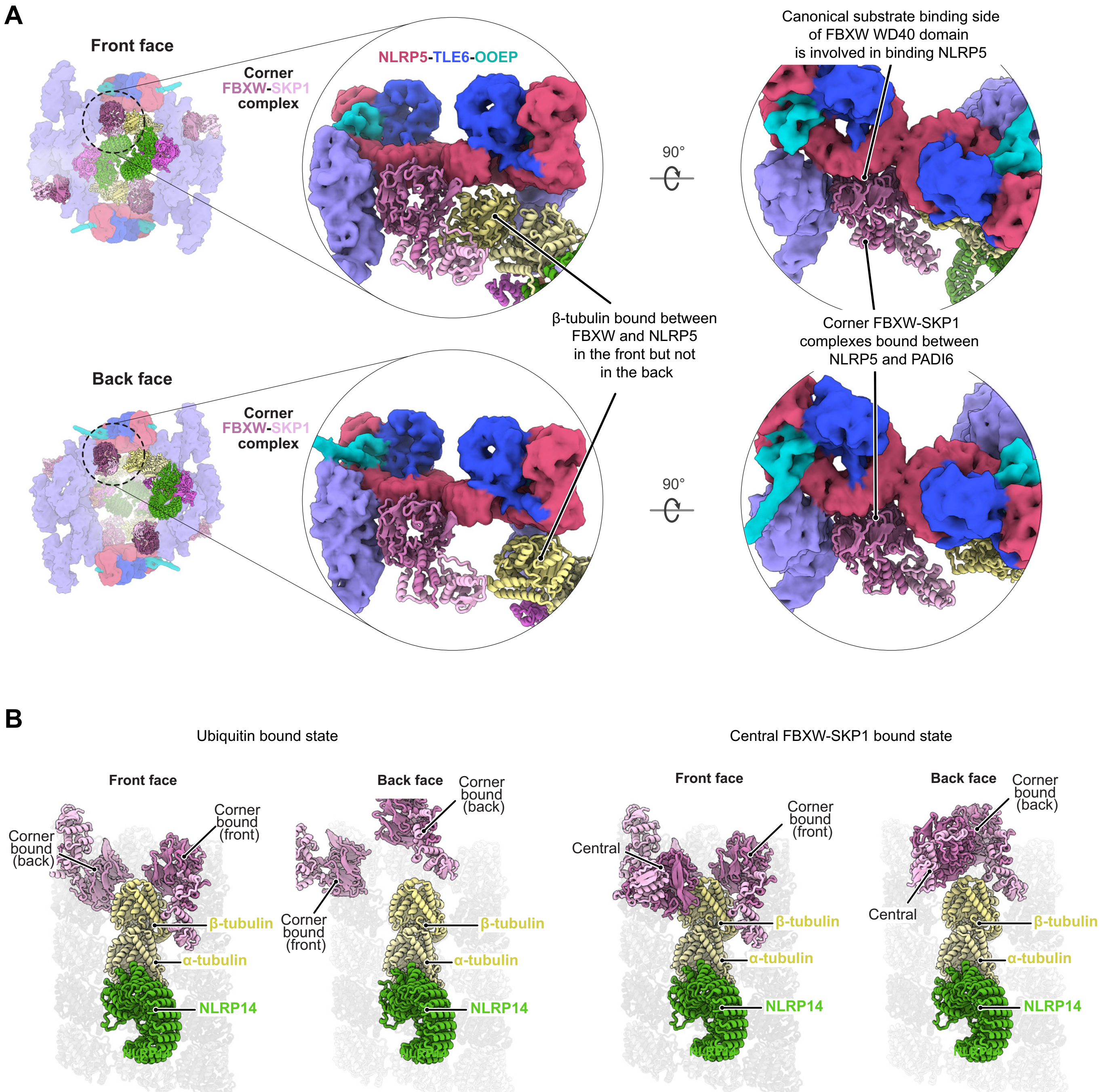

Figure EV7. Interaction of FBXW-SKP1 complexes with UHRF1-UBE2D modules within the CPL repeat.

(**A**) Models showing the interaction of corner FBXW-SKP1 complexes with the UHRF1-UBE2D modules on the front and back faces of the CPL repeat. On the front face, β-tubulin is positioned between FBXW-SKP1 and NLRP5, forming a compact interface that stabilizes the interaction with the UHRF1-UBE2D module. In contrast, on the back face, the interface is more open, and β-tubulin does not contact either component. Notably, the WD40 surface of FBXW-SKP1 that mediates substrate recognition in other F-box proteins is occluded through its interaction with NLRP5. (**B**) Interactions between αβ-tubulin-NLRP14 complexes on the front and back faces of the CPL repeat unit and FBXW-SKP1 complexes. The ubiquitin-bound state is shown on the left, and the state containing the central FBXW-SKP1 complex is shown on the right. To place these interactions in the structural context of a microtubule, the tubulin heterodimer is overlaid onto a microtubule lattice.

 © The Author(s)

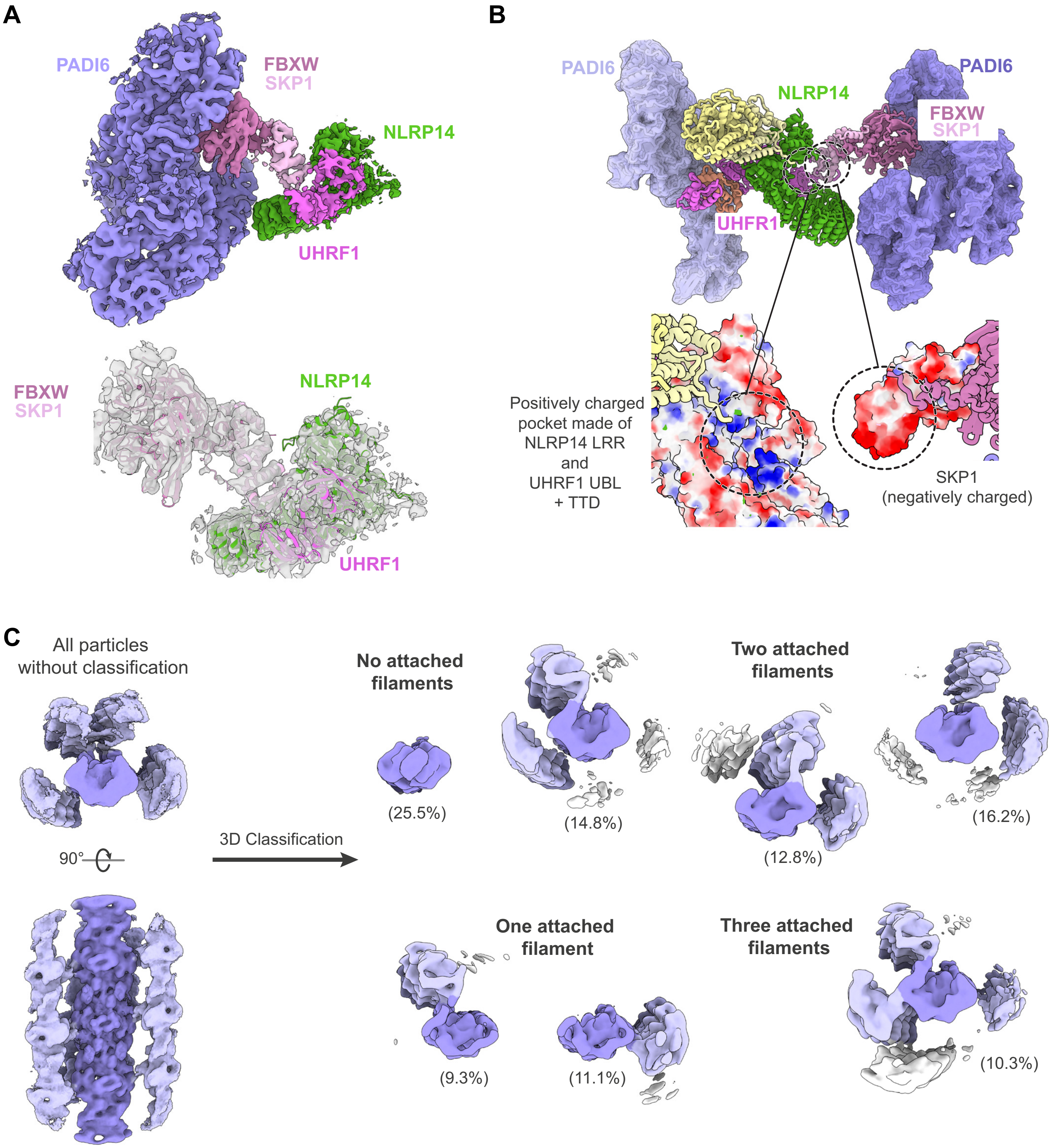

Figure EV8. Inter-filament contacts within CPLs.

(**A**) Subtomogram averaging density of the proteins linking two CPL filaments (top) with the corresponding atomic model of FBXW–SKP1, NLRP14, and UHRF1 fitted into the density (bottom). (**B**) View rotated 180° relative to Fig. 6A showing that SKP1 engages the neighboring CPL filament through a negatively charged patch (red) that binds a positively charged pocket (blue) formed by the NLRP14 LRR and the UHRF1 UBL and TTD domains. White color denotes neutral charge. (**C**) Reconstruction of all particles corresponding to CPL repeats at a box size of 1528 Å prior to 3D classification (left) and the resulting 3D classes after classification, with the percentage of particles assigned to each class indicated in brackets.

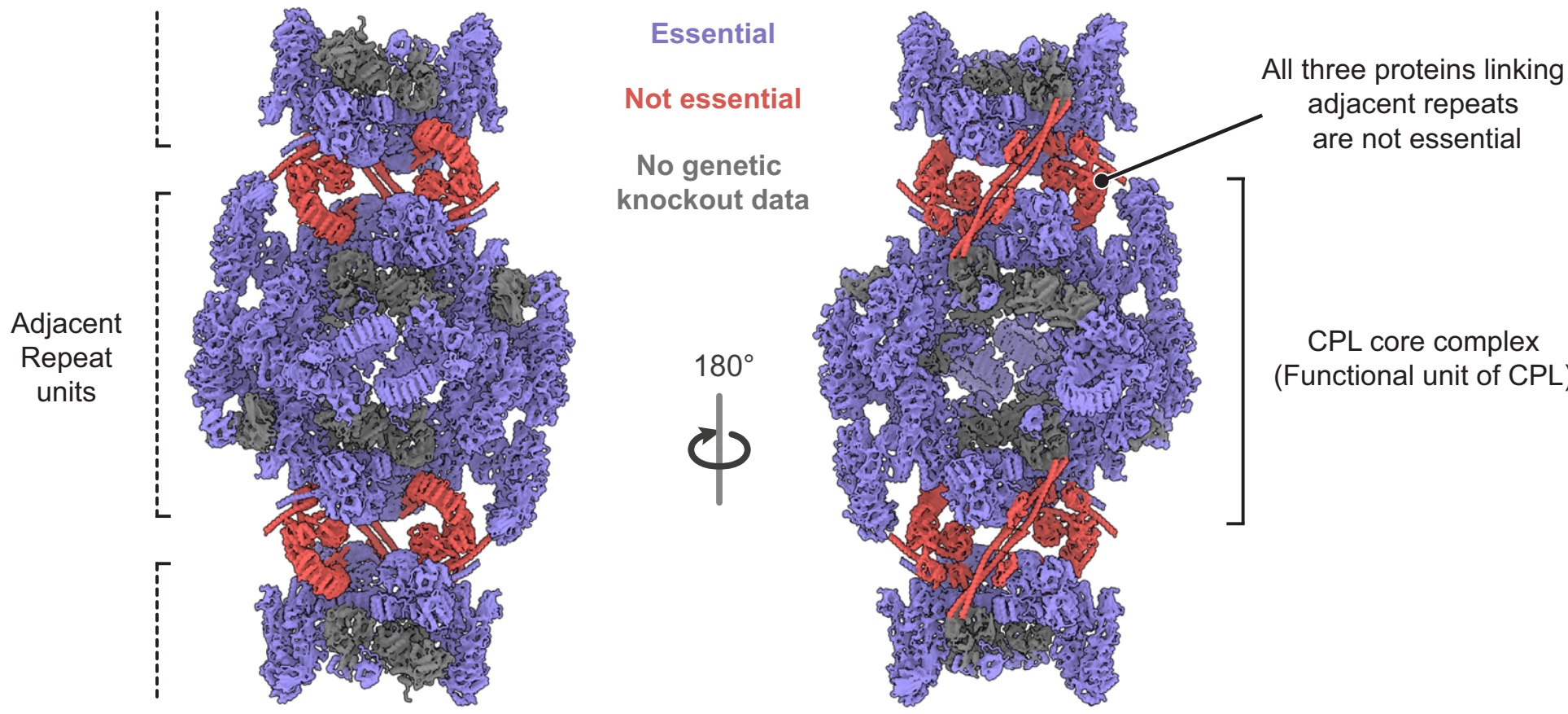

Figure EV9. Mapping of genetic loss-of-function phenotypes onto the CPL structure.

Proteins identified in the CPL repeat are colored according to the developmental phenotypes reported upon gene disruption in mice. Proteins whose loss results in embryonic lethality or infertility are shown in lavender, proteins whose deletion permits viable progeny are shown in orange, and proteins for which genetic data are currently unavailable are shown in gray. This mapping highlights that factors essential for development form the CPL scaffold or reside within the CPL core complex, whereas proteins required for higher-order filament assembly are dispensable for viability.

 © The Author(s)

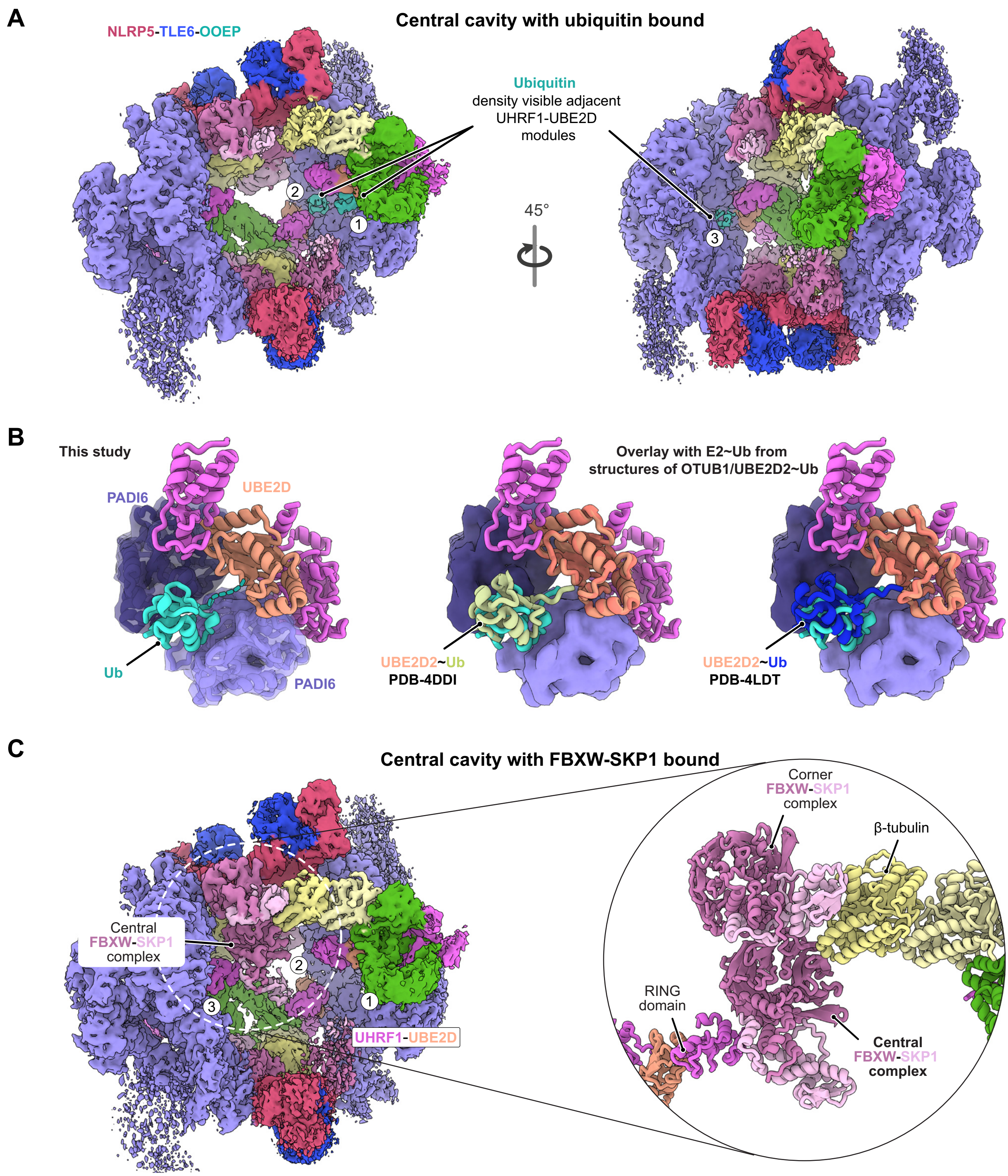

Figure EV10. Structural states of the CPL core complex.

(**A**) Subtomogram averaging density of the CPL repeat unit in ubiquitin-bound state showing ubiquitin density (teal) adjacent to the UHRF1-UBE2D modules within the central cavity, consistent with ubiquitin conjugated to UBE2D. (**B**) Comparison of the open E2-Ub state within CPLs with those observed in OTUB1-bound E2-Ub structures (PDB-4DDI (Juang et al, 2012) and PDB-4LDT (Wiener et al, 2013)). The E2-Ub within CPLs adopts an open conformation closely resembling that within the OTUB1-bound E2-Ub structures. (**C**) Density of the CPL repeat unit in the state containing an additional central FBXW–SKP1 complex and lacking detectable ubiquitin density. The inset shows a close-up of the central FBXW–SKP1 complex, which contacts β-tubulin of the back-face complex and lies adjacent to the RING domain of UHRF1.

© The Author(s)
